# Screen for signatures of positive selection in the core genome of Staphylococcus epidermidis

**DOI:** 10.1099/mgen.0.001692

**Published:** 2026-04-20

**Authors:** Callum O. Rimmer, Jonathan C. Thomas

**Affiliations:** 1 School of Science and Technology, Nottingham Trent University, Nottingham, UK

**Keywords:** antimicrobial resistance, core gene, NirB, nitrite reductase, positive selection, siroheme

## Abstract

*Staphylococcus epidermidis* is a commensal skin bacterium and the leading cause of medical-device-associated infections. Although previous studies have investigated the phylogenetic diversity of the species, the level of positive selection on the core genome has yet to be explored in *S. epidermidis*. Here, we present a comprehensive genome-wide screen for signatures of positive selection in this species. A curated dataset of 1,003 whole-genome sequences was created, which represented the global diversity of *S. epidermidis*, including all previously identified clades and genetic clusters. A 100-strain subset, which retained the diversity of the collection, was created by pruning the species-level tree with treemmer; core genes present in all genomes were extracted with Roary and used for positive selection analysis (*n*=826). Site-level analysis was performed using PAML with omegaMap for confirmation. Selection along branches separating clades A and B was investigated using PAML branch-site models and HyPhy. PAML site analysis revealed 16 genes under selection, including 6 hypothetical genes, most of which were linked to metabolism or transport. Several genes were associated with antimicrobial resistance, including *ileS*, which confers resistance to mupirocin. Nearly a third of the genes under selection were components of interconnected pathways that collectively provide essential cofactors for the NirB enzyme subunit. No genes showed evidence of positive selection in both the PAML and HyPhy branch-site analyses. Our analysis reveals the extent to which positive selection is operating on the core genome of *S. epidermidis*, driving clade-associated mutations in multiple core genes, and identifies candidate genes that may have important roles in the fitness of the species.

**Impact Statement**

*Staphylococcus epidermidis* is a coagulase-negative staphylococcal species and a ubiquitous colonizer of human skin. While it is a leading cause of medical-device-associated infections, little is understood of the role positive selection has in the evolution of this species. Utilizing various bioinformatic approaches for detecting signatures of selection, our analysis shows positive selection is driving clade-associated mutations in multiple core genes, several of which are associated with antibiotic resistance.

## Data Availability

All data for in-house sequenced isolates are available under BioProject accession number PRJNA1159912 and BioSample accession numbers SAMN43587831–SAMN43587842. Illumina raw read data and Nanopore basecalled data were submitted to SRA and are available via accession numbers SRR30669856–SRR30669867 and SRR30669822–SRR30669833, respectively. Genome assemblies were deposited to GenBank; see Table S1 for accession numbers. Excel versions of supplementary tables, multiple sequence alignments, Newick files for both phylogenetic trees and custom scripts are available on FigShare (https://doi.org/10.6084/m9.figshare.31664251).

## Introduction

*Staphylococcus epidermidis* is a Gram-positive opportunistic pathogen and member of the coagulase-negative group of staphylococci. A ubiquitous colonizer of the human skin, it is a frequent source of both contamination and infection, especially amongst hospital patients with indwelling medical devices, such as catheters or central venous lines [1]. The population structure of *S. epidermidis* has been extensively investigated [2,8], revealing a highly structured phylogeny composed of three major clades (A–C) based on the core genome [9,11], while multilocus sequence types (STs) cluster into six different genetic clusters (GCs) [7,12]. GCs one, five and six have been linked to pathogenic or generalist lifestyles and mostly correspond to clade A. STs belonging to GCs two and four (clade B) have been isolated from more niche environments, including sources such as rice grains and wild mice, and often lack the genomic features linked to more pathogenic lifestyles. GC three (clade C) has been identified as a genetic sink with a large degree of admixture from the other clusters and appears highly recombinant [7,12]. More recently, an alternative approach has been used whereby a score is attached to the collective sum of either accessory genes or SNPs in shared genes for a given strain and has yielded improved accuracy for predicting isolation source [13]. A recent pan-GWAS analysis supported previous findings that the two major clades of *S. epidermidis* are enriched for different genes. However, it was also shown that these clades are associated with different body sites; isolates from clade B were enriched for foot sites, whereas those from clade A showed no significant association with any particular location [14].

Positive selection is a process whereby mutations with a fitness advantage increase in frequency in the population, driven by selective pressures resulting in either directional, diversifying or balancing selection [15]. Analysis of patterns of positive selection across the core genome may identify genes that play an important role in adapting to the environment. Since next-generation sequencing technologies have become more widely accessible, the number of publicly available genomes has grown exponentially. This has given more power to studies analysing selection as a larger sample of genomes, more representative of the overall population, can be screened. When analysing selection, most tools focus on the ratio of non-synonymous to synonymous mutations (*ω*) within genes, with the premise that a *ω* value of 1 represents neutral evolution, *ω*<1 represents purifying selection and *ω*>1 represents positive selection, particularly diversifying selection. While *ω*=1 is regarded as the threshold for selection, it is rarely true for a gene to evolve strictly neutrally across the entire length of the gene, as evolution is typically constrained at functionally important sites within a gene. Hence, the development of site models that allow the *ω* ratio to vary across a gene has been beneficial, as genes can now be flagged as under selection where previously the signal of selection would have been diluted, since previous methods relied on an average *ω* value across the whole gene. It is well understood that the success of *S. epidermidis* as an opportunistic pathogen is due to its extensive genome plasticity, driven by extensive horizontal gene transfer between divergent clones [11]. However, while many studies have examined the role of accessory genome elements such as SCC*mec*, *ica* or IS*256*, in adaptation to specific niches, comparatively little work has investigated signatures of positive selection in the core genome and their potential contribution to *S. epidermidis*’ fitness and persistence across diverse environments.

Identifying genes under selection using *in silico* approaches is well-established in multiple bacterial species [16,20]. In *S. epidermidis*, balancing selection has previously been investigated [21]. Here, we perform a genome-wide scan for signatures of positive selection. In this study, we identify genes under positive selection across the core genome of a phylogenetically representative subset of the global *S. epidermidis* population. Specifically, we identify two sets of genes: (1) those where selection acted in any lineage throughout the species and (2) those that are under selection between the two major clades of *S. epidermidis* (clades A and B).

## Methods

### Bacterial strains

We acquired 12 *S*. *epidermidis* strains from other laboratories whose STs belonged to the underrepresented GC2 (listed as in-house strains in Table S1, available in the online Supplementary Material). Strains were cultured at 37 °C overnight on trypticase soy agar (Oxoid, UK). Single colonies were transferred to 10 ml trypticase soy broth (TSB) (Oxoid, UK) and incubated overnight at 37 °C. Strains were stored at −80 °C in a mixture of TSB and 15% glycerol (v/v). Genomic DNA was extracted with a QIAGEN DNeasy kit according to the manufacturer’s instructions [using 10 µl lysostaphin (1 mg ml^−1^) for cell lysis].

### DNA sequencing and hybrid assembly

Illumina and Nanopore sequencing of the 12 *S*. *epidermidis* isolates was performed as described previously [22]. All software was run according to default parameters unless otherwise noted. Fast5 sequencing reads were basecalled with the high-accuracy model of Guppy v3.6.1 (Oxford Nanopore, UK). Sequence adapters were filtered using Porechop v0.2.4 [23] with middle and end thresholds of 85% and 95%, respectively. Reads were filtered based on quality and length using Filtlong v0.2.1 [24]. Canu v2.2 [25] was used to assemble overlapping reads into one contiguous sequence. The assembly was polished with four iterations of Racon v1.4.20 [26], followed by Medaka (-m r941_min_high_g360) v1.6 [27] and Nanopolish v0.13.2 [28]. Trimmomatic v0.39 [29] was used to ensure all adapter sequences were removed from Illumina data. The output from Nanopolish [28] was error-corrected with Illumina data using Racon and Pilon v1.24 [26,30]. The assemblies were manually trimmed and re-orientated to *dnaA* using Circlator v1.5.5 [31]. Assembly quality was determined using CheckM v1.1.3 [32] and average nucleotide identity (ANI) was compared to the *S. epidermidis* type strain NCTC 11047^T^ using FastANI v1.1 [33]. All assembly metrics are available in Table S1.

### Public whole-genome sequences

In October 2020, 1,272 whole-genome sequences (WGSs) of *S. epidermidis* were downloaded from online repositories: 862 from NCBI, 72 from PubMLST [34], 97 from Dryad (DOI: 10.5061/dryad.82jq4; 10.5061/dryad.br0h8) and 241 from figshare (DOI: 10.6084 /m9.figshare.7058543.v1). In May 2022, an additional 351 WGSs were downloaded from the National Center for Biotechnology Information (NCBI) and 19 from PubMLST [34]. This resulted in a collection of 1,642 WGSs before filtering. As described previously, ANI was calculated using FastANI with *S. epidermidis* type strain NCTC 11047^T^ as a reference. Assembly quality was determined by CheckM. Any genome with an ANI value of <95% compared to the type strain was removed. Genomes with a size of less than 2.325 Mbp or greater than 2.9 Mbp, N50 less than 50 kbp, more than 180 contigs, containing any ambiguous bases, contamination of more than 2.5% or completeness of less than 95% were removed. In total, 651 genomes were removed, leaving 991 WGSs for analysis. The 12 hybrid assemblies sequenced in-house were added to the public dataset, leaving 1,003 genomes to carry forward for analysis.

### Sequence typing and genetic cluster assignments

STs were assigned using *mlst* [35] with the *S. epidermidis* scheme [6]. Novel loci were submitted to the PubMLST *S. epidermidis* database. STs were assigned to GCs with BAPS v6 [36] using a codon linkage model. Allelic profiles and their alleles were downloaded for all 1,158 STs in PubMLST’s *S. epidermidis* database as of 24 June 2022. Allele sequences were trimmed and reverse-complemented where necessary to maintain the +1 reading frame, before being concatenated into a single sequence. BAPS was run independently five times, with the maximum number of clusters set from 11 to 20. All ST and GC information are available in Table S1.

### Determining the core genome and phylogenetic analysis

All 1,003 genome assemblies were re-annotated with Prokka v1.14.6 [37] to ensure standardized gene annotations across the dataset. Roary v3.13 [38] was used to determine the core genome using gff files from Prokka. Flags ‘-e’ and ‘-n’ were used to create core gene alignments with MAFFT and flags ‘-cd 100’ and ‘-z’, defining core genes as those present in 100% of the dataset and keeping intermediate files, respectively. Roary identified 533 genes as core; however, 4 were multi-copy genes and removed, leaving 529 core genes. The multiple sequence alignments (MSAs) for the 529 core genes were scanned for recombination using RDP5 v5.5 [39] using four recombination tests: RDP, GENECONV, Chimaera and MaxChi. The highest acceptable *P*-value was set to 0.05 and Bonferroni correction was applied. Genes where recombination events were detected by three or more tests were removed, leaving 467 recombination-free core gene MSAs for further analysis. All 467 MSAs were concatenated into a single alignment for all 1,003 strains using FASconCAT v1.11. A maximum-likelihood tree was produced using RAxML-NG v1.1.0 [40] with tree model GTR+G+I and 100 bootstrap replicates, midpoint rooted and visualized with iTOL v5 [41].

### Creating a reduced dataset for positive selection analysis

Positive selection analysis on gene alignments of 1,003 strains was not computationally feasible; it was necessary to reduce the size of the dataset but still reflect the phylogenetic structure of the original tree. Treemmer v0.3 [42] was used to prune the 1,003-strain phylogenetic tree down to 100 strains using ‘-X 100’ based on metadata containing the GC assignments, complete assemblies and strains sequenced in-house (-lm). The list of strains in the pruned tree was used as the reduced dataset. A new core genome was created with Roary as described above; both PRANK [43] and MAFFT were used for sequence alignments. In this reduced dataset, 1,387 genes were identified as core. Core gene MSAs were checked for recombination with RDP5 as previously described; 381 genes were removed due to recombination, as high levels of recombination can result in a high rate of false positives [44]. Alignments were scanned for gaps and stop codons with custom scripts; any genes with frameshift mutations in the MAFFT alignments were removed from the dataset, and terminal stop codons were removed using AliView [45]. After curation, 826 genes were carried forward for analysis. Gene trees were generated using RAxML-NG with tree model GTR+G+I from the individual PRANK alignments.

### Positive selection analysis

For site-based analysis, the CODEML package within PAML v4.9 [46] was used to determine if any genes were under positive selection. Each gene was tested, using individual gene trees and the PRANK alignments, against two sets of site models: M1a vs. M2a and M7 vs. M8 [47,48]. Each pair consists of a test model, which allows selection and a null model where no selection is allowed. The log-likelihood scores (ℓ) for each were then compared using the likelihood-ratio test (LRT), calculated as 2Δℓ.

LRT values were converted to *P-*values using the chi2 program within PAML (df=2), and the false discovery rate [49] was used for multiple correction testing. Three replicates were performed for each model, and genes were classed as under positive selection if the LRT was significant for each replicate. Positively selected sites were identified using the Bayes empirical Bayes (BEB) test [50] within PAML. The relative position of each locus in the *S. epidermidis* strain RP62A genome was investigated to identify potential selection hotspots. In addition to visual inspection, a permutation test framework implemented with the R package regioneR was used [51]. For regioneR, the dataset was randomized using the resampleRegions function, and genes under selection were compared against the remaining genes in the collection based on the mean distance to the closest locus over 100,000 permutations.

omegaMap was used as an independent confirmatory test for genes identified as under positive selection by the PAML site models [52]. omegaMap is a phylogeny-free approach and only requires gene alignments. Variation in *ω* for each gene’s MSA was determined using the variable model, where each sequence was split into blocks of three codons; sites within each block share the same *ω*. This relatively small window was chosen to ensure any signals of selection were not overly diluted. Codon frequencies calculated by PAML were used for omegaMap. An inverse distribution of ω was used, with minimum and maximum values of 0.01 and 50, respectively. The Monte Carlo Markov chain was run for 250,000 iterations and 10 orderings.

**Fig. 1.**
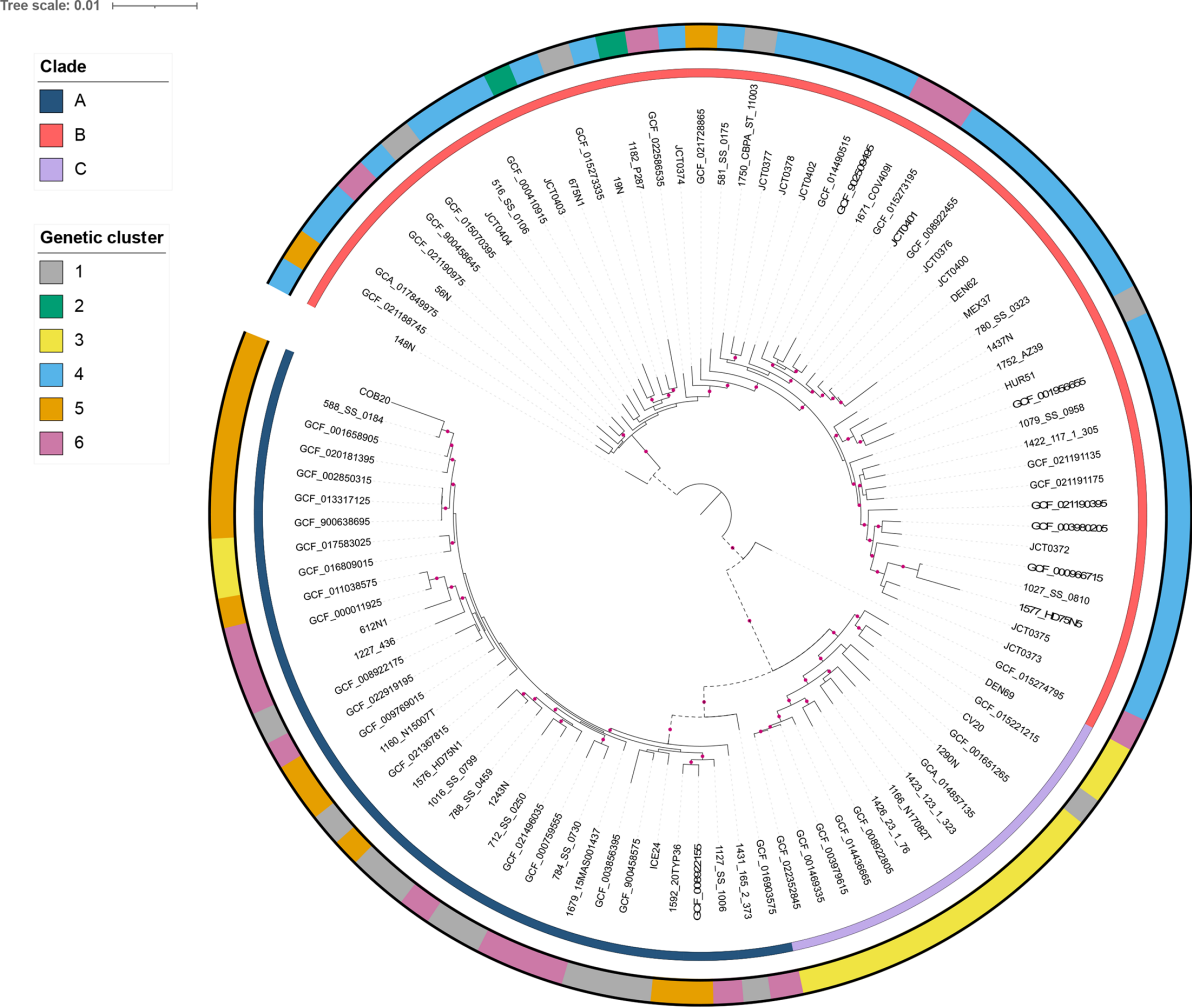
Maximum-likelihood tree for the 100-strain subset, based on the concatenation of 826 core genes. Purple circles at nodes represent bootstrap support of 75%. Inner and outer rings show clade and genetic cluster, respectively. Branches with dashed lines were chosen for branch-site analysis with PAML and HyPhy.

The 100-strain subset core gene MSAs from PRANK were also concatenated into a single sequence and a species tree was generated with RAxML-NG with tree model GTR+G+I and 1,000 bootstrap replicates; the generated tree was midpoint rooted and visualized with iTOL. To identify whether genes are under selection along the four branches between clades A and B in the species tree (Fig. 1), PAML branch-site models A and A_null_ were used [53,54]. Five replicates were performed for each model using different initial values of *ω* (0.4–1.5). The highest log-likelihood score of the five replicates for each model was used to perform the LRT. LRT values were converted to *P-*values using the chi2 program from PAML (df=1; false discovery rate (FDR) corrected). Given that omegaMap is a phylogeny-free approach, it could not be used as the independent test for selection on specific branches. Instead, MEME, BUSTED and aBSREL from the HyPhy package were used as alternative independent tests to identify genes under selection along the four branches [55,58]. MEME was run with a *P*-value cut-off of 0.05 and 500 bootstraps. BUSTED and aBSREL were run with synonymous-rate variation. All three programmes were run with ‘--kill-zero-lengths No’. FDR correction was applied to the branch-site data. As the computing time for HyPhy is considerably less than PAML, we also ran HyPhy on all core genes. Clusters of orthologous genes (COG) categories for all genes under selection were assigned using COGclassifier [59].

The PAML analysis was performed in batches of 100 genes using our departmental high-performance computing cluster (HPC). The average runtime per gene per replicate was ~4 h; therefore, across eight sets of genes, this resulted in an approximate total runtime of 32 h for a single replicate per model. For all PAML models, this equates to roughly 16 days of continuous runtime.

### Protein structures

Haplotypes for each gene, defined only by sites under selection, were ranked according to a BLOSUM62 matrix using the R package Biostrings v2.76 [60]. Protein structures were predicted using ColabFold v1.5.5 [61] for the most common haplotype sequence for each gene, as well as haplotypes with a BLOSUM score of less than zero. ChimeraX v1.10 was used for structural visualization and alignment [62]. The effect of mutations on protein stability was assessed by estimating changes in folding free energy (ΔΔG) using FoldX v5.1 [63].

## Results

### Curated database of WGSs

We created a final curated database of 1,003 WGSs. The average genome size was 2.52 Mbp, and 16 genomes were comprised of a single contig. Excluding complete or nearly complete genomes (contigs <10), the average N50 was 144 kbp, while the average number of contigs was 72. For the reduced dataset of 100 genomes, the average N50 was 427 kbp, while the average number of contigs was 64.

### MLST and genetic clusters

The program BAPS (Bayesian Analysis of Population Structure) has previously been used to classify *S. epidermidis* STs into distinct genetic clusters. Since 2015, the *S. epidermidis* MLST database has expanded from 588 STs [12] to 1,158 STs, downloaded on 13 July 2022. Based on five independent runs of the software using the updated ST database, BAPS identified seven genetic clusters. Twenty-six STs that were previously assigned a GC [12] were assigned a different GC with the new dataset, although 24 of these involved GC3, a known genetic sink for the other clusters and subject to a large amount of admixture [7,12]. Each replicate produced identical results; however, support for GC7 was limited to only seven STs. There was a diverse array of STs in our full curated dataset, with 278 unique ST profiles. All genetic clusters except for GC7 were represented in the reduced dataset; GC1, GC3, GC5 and GC6 each had 15 strains. GC2 had two strains and GC4 had 38 strains. This corresponded to 35 strains in clade A, 51 in clade B and 14 in clade C.

### Phylogenetic analysis

Fig. S1 shows the *S. epidermidis* phylogeny using the curated dataset of 1,003 WGSs from globally distributed strains. Our tree shows consistent clade structure compared to previous studies [9,10], with the majority of isolates clustering into clades A and B. Clade A mostly comprised strains from GC1, GC5 and GC6, clade B mostly contained strains from GC4 and clade C predominantly corresponded to isolates from GC3. Strains belonging to GC2 also clustered with clade B as previously shown [10]. No WGSs from GC seven were present in the dataset. While clade A was diverse with most strains belonging to three different GCs, there was clear separation of GC5 compared to GC1 and GC6. This is unsurprising considering strains from GC5 appear to be adapted to a hospital environment, while the majority of staphylococcal sequencing studies are focussed on clinical isolates [7,12]. Fig. 1 shows the phylogeny produced using the reduced dataset of 100 WGSs; this tree shares the same clade structure as the complete 1,003-strain dataset (Fig. S1) and shows that the strains used for positive selection analysis are representative of the global population of *S. epidermidis*.

### Positive selection analysis

Nested site models from PAML identified 16 genes under positive selection (1.94% of core genes). All 16 were significant under the relaxed model set M7M8, while 10 were significant under the more stringent model set M1M2 (Table 1). Six hypothetical genes were under selection, five of which were significant with both model sets. omegaMap was also used to ensure that the list of genes under selection could be confirmed via an alternative independent test. Data from omegaMap agreed with 15 of the 16 genes flagged as under selection by PAML, with posterior probabilities of >0.9 for each of the sites identified by the BEB test. omegaMap did not provide support for positive selection in the gene group_10327, as no sites showed a posterior probability of >0.4. In contrast, the BEB analysis implemented in PAML identified two sites (positions 335 and 341) as being under positive selection. group_10327 did not appear to be poorly aligned or contain significant signals of recombination from RDP5, which could have explained the disagreement between the two methods. While no COG categories were enriched for genes under selection, these genes belonged to a diverse set of classes, with 10 different COG categories across the 16 genes (9 COG categories for the 15 genes confirmed by omegaMap). Positions of genes under selection were determined in the *S. epidermidis* strain RP62A and plotted to visualize. No putative hotspots for selection were identified, and permutation tests showed no significant difference (*P*=0.054) in the mean distance between loci for the genes under selection and the rest of the dataset (Fig. S2).

**Table 1.** Genes under site-level selection based on PAML models M1M2 and M7M8 The Bayes empirical Bayes test from PAML model 8 was used to predict specific sites under selection. Hypothetical genes were denoted with the ‘group_’ prefix by Roary.

Gene	M1M2 *P*-value	Model 8 sites	M7M8* P*-value	RP62A locus tag	COG category	COG definition
*csd*	<0.001	321 S†	<0.001	SERP_RS02565	E	Amino acid transport and metabolism
group_1172	<0.001	66 G*; 160 R*; 202 S†	<0.001	SERP_RS11475	H	Coenzyme transport and metabolism
group_1349	<0.001	183 Y*; 195 N†; 213 G†; 218 A*; 243 S†	<0.001	SERP_RS04470	V	Defence mechanisms
group_2443	<0.001	151 H†; 156 G†; 214 P*; 552 L†; 584 V*	<0.001	SERP_RS11155	J	Translation, ribosomal structure and biogenesis
group_3177	<0.001	463 S†	<0.001	SERP_RS02575	O	Post-translational modification, protein turnover, chaperones
group_10276	<0.001	107 K†; 167 D†	<0.001	SERP_RS10320	M	Cell wall/membrane biogenesis
*hisI*	<0.001	122 D†	<0.001	SERP_RS11315	E	Amino acid transport and metabolism
*ndhB*	<0.001	158 R*; 441 L†	<0.001	SERP_RS00545	C	Energy production and conversion
*sirC*	<0.001	54 A†; 89 G†	<0.001	SERP_RS10840	H	Coenzyme transport and metabolism
*yfcA*	<0.001	13 V†; 211 F†	<0.001	SERP_RS06320	P	Inorganic ion transport and metabolism
*cysG*	ns	32 S†; 242 I†; 270 R†	<0.001	SERP_RS09915	H	Coenzyme transport and metabolism
group_10327‡	ns	335 H†; 341 Q*	<0.001	SERP_RS12385	T	Signal transduction mechanisms
*ileS*	ns	588 V*	<0.001	SERP_RS03840	J	Translation, ribosomal structure and biogenesis
*nasD*	ns	27 Q†; 757 V*	<0.001	SERP_RS09925	C	Energy production and conversion
*rluD_3*	ns	77 V*; 174 A†; 179 L*; 201 K*	<0.001	SERP_RS03005	J	Translation, ribosomal structure and biogenesis
*yheS*	ns	59 H*; 346 R*; 352 V*	<0.001	SERP_RS08285	R	General function prediction only

*Posterior probability >95%.

†Posterior probability >99%.

‡Gene not supported by omegaMap.

ns, Not significant.

PAML branch-site models A/A_null_ identified three genes under selection (0.36% of core genes, Table 2) along the branches between clades A and B (highlighted as dashed branches in Fig. 1). The BEB test only identified sites under selection with a posterior probability of >0.95 in one gene, group_1315. No genes were shared between the analyses of PAML branch-site models and HyPhy (both aBSREL and BUSTED). While aBSREL and BUSTED did not identify selection in group_1315, MEME did support two of the sites highlighted by the BEB test. For HyPhy, aBSREL supported 13 of the 826 core genes as showing signatures of positive selection across at least 1 of the 4 branches separating clades A and B. MEME identified sites under selection for the three genes identified as significant by PAML’s branch-site model analysis, as well as 10 of the 13 genes supported by aBSREL.

**Table 2.** Genes with signatures of branch-site level selection based on PAML models A/A_null_ and HyPhy aBSREL indicates how many of the four branches chosen to analyse are under selection. MEME presents a list of sites under selection, similar to PAML.

Gene	PAML *P*-value	PAML BEB sites	aBSREL: branches under selection	MEME: sites under selection	RP62A locus tag	COG category	COG definition
group_1315	<0.001	40†, 62*, 76†	ns	16, 40, 76	SERP_RS0040	–	–
*gtfA_3*	<0.001	–	ns	2, 80, 167, 169, 258, 313, 315, 320, 323, 348	SERP_RS0123	M	Cell wall/membrane/envelope biogenesis
*recQ_2*	<0.001	–	ns	98, 163	SERP_RS0204	L	Replication, recombination and repair
*atpF*	ns	ns	1	ns	SERP_RS08575	C	Energy production and conversion
group_1158	ns	ns	1	68, 196, 296, 376	SERP_RS06800	U	Intracellular trafficking, secretion and vesicular transport
group_1429	ns	ns	1	ns	SERP_RS08475	–	–
group_3287	ns	ns	1	22, 31, 63, 78	SERP_RS08590	–	–
*metK*	ns	ns	1	162, 391	SERP_RS06670	H	Coenzyme transport and metabolism
*plsC*	ns	ns	1	68, 203	SERP_RS06380	I	Lipid transport and metabolism
*rhtC*	ns	ns	1	94	SERP_RS00415	E	Amino acid transport and metabolism
*ribE*	ns	ns	1	155, 202	SERP_RS06555	H	Coenzyme transport and metabolism
*rpsQ*	ns	ns	1	23	SERP_RS09145	J	Translation, ribosomal structure and biogenesis
*sigB*	ns	ns	1	30	SERP_RS08395	K	Transcription
*tal*	ns	ns	1	50, 52, 162	SERP_RS06595	G	Carbohydrate transport and metabolism
*yedJ*	ns	ns	1	43, 157	SERP_RS08490	J	Translation, ribosomal structure and biogenesis
*yghA*	ns	ns	1	ns	SERP_RS09595	I	Lipid transport and metabolism

ns, Not significant after FDR correction.

*Posterior probability >95%.

†Posterior probability >99%.

### Distribution of mutations between clades for sites under selection

**Fig. 2.**
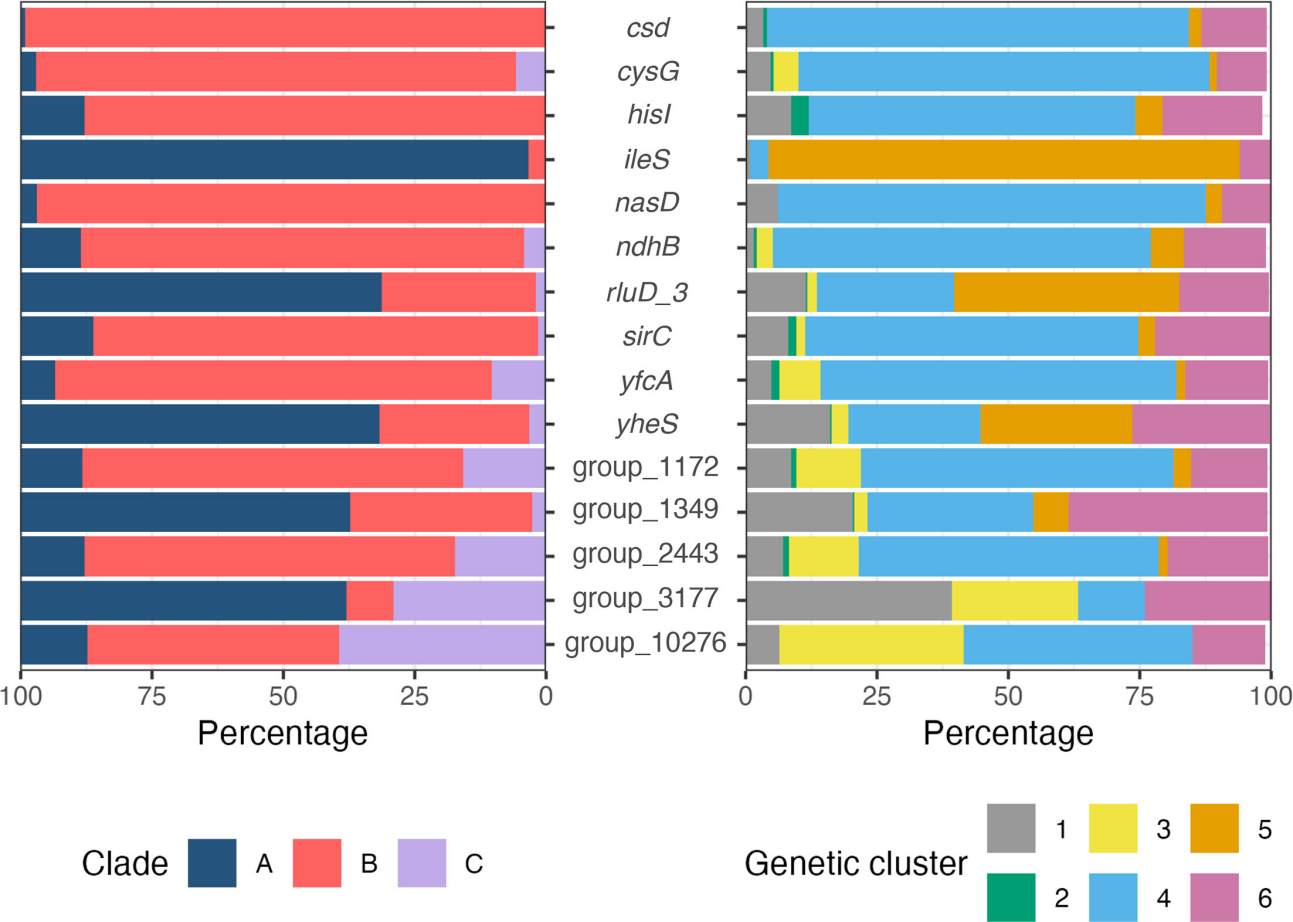
Distribution of minor alleles for each gene under site-level selection from PAML, split by clade (left) and genetic cluster (right). Analysis is based on the alignment data for all 1,003 strains. Minor alleles are defined as all alleles excluding the most frequently observed at a given site.

After identifying specific sites under selection, we analysed the frequency of alleles at these sites in the original alignments from our 1,003-strain dataset. For the 10 characterized genes under selection, most of the minor alleles (all alleles excluding the most frequently observed at a given site) in *ileS*, *rluD_3* and *yheS* could be attributed to clade A, at 97, 69 and 54% of all minor alleles, respectively (Fig. 2). While minor alleles were evenly distributed across genetic clusters for *rluD_3* and *yheS*, 90% of minor alleles at the selected site in *ileS* were from hospital-associated GC5 strains. Although only one site was under selection for *ileS*, for both *rluD_3* and *yheS*, at least three sites showed evidence of selection. Despite most minor alleles belonging to clade A strains for these three genes, this was not consistent across all sites. For codon 179 in *rluD_3*, 75% of strains encoding a leucine were clade B strains, while at codon 346 in *yheS*, 97% encoding arginine were clade B. For the remaining seven characterized genes, minor alleles predominantly belonged to clade B strains, ranging from 83 to 97% of all minor alleles. Only two selected sites had minor alleles that were primarily linked to strains belonging to clade C: strains encoding valine at codon 242 in *cysG* and threonine at codon 13 in *yfcA*. As GC1, GC5 and GC6 were well represented in our dataset as part of clade A, we wanted to investigate whether there were any differences in SNPs across these clusters. At codons 77 and 179 for *rluD_3*, over 80% of GC1 and GC6 strains encoded the major allele, while ~78% of GC5 strains encoded the minor allele. This pattern was also seen at two codons for *yheS*.

For the five hypothetical genes confirmed to be under selection by omegaMap, minor alleles were largely observed in clade A strains for group_1349 and group_3177 and clade B strains for group_1172, group_2443 and group_10276. group_10276 also featured the most minor alleles attributed to clade C strains.

### Structural analysis

Structural alignments in ChimeraX between the most common haplotype sequence for each gene and selected haplotypes showed a maximum root mean square deviation (RMSD) value of less than 1 Å (Table S2), indicating no mutations had a significant impact on overall protein structure (see Fig. S3 as an example). There were numerous mutations, however, which involved a shift in either charge or polarity that may affect the protein’s function (see Table S3). Changes in folding free energy (ΔΔG) ranged from highly stabilizing (–3.39 kcal/mol) to destabilizing (4.81 kcal mol^−1^; see Table S3).

## Discussion

Identifying genes under positive selection is key to understanding how natural selection has shaped the evolution of an organism, both at the species level and within specific lineages of a species [64]. While it is expected that detectable signals of selection would be found across most bacterial species, the distribution of selected sites and their association with specific genes or pathways can yield insights into functionally important aspects of a species’ evolution. We have assembled a dataset comprising 100 high-quality genomes that provide a proportional representation of the 3 major clades and 6 genetic clusters of *S. epidermidis* to detect positive selection within the core genome.

Before the development of site models that allow for variation in *ω* across individual sites in a sequence, studies were mostly limited to gene-wide averages of the dN/dS ratio, meaning the strength of selection had to cover a substantial portion of the gene for it to be possible to sufficiently detect the signal to indicate a gene is under selection. While neutral theory plays a vital role in population genomics [65,66], multiple studies looking at signatures of selection within the core genome of bacterial species have observed extremely low average *ω* values: 0.16 in *Legionella pneumophila* [20], 0.05 in *Verminephrobacter* [16] and 0.064 in *Streptococcus dysgalactiae* [19]. We observed an average *ω* value of 0.12 across all genes tested for selection, indicating that most core genes within *S. epidermidis* are also under strong purifying selection; this includes genes that were themselves identified as under positive selection, for example, group_1349, which had five sites under selection, still had a gene-wide average *ω* value of 0.43. This highlights the importance of using site or branch-site models when analysing selection instead of gene-wide dN/dS values. There are exceptions to this, such as high sequence diversity, for example, in studies screening for selection across multiple species [67,69].

### Selection at the site level

We detected 16 genes under selection from PAML site models, ~1.9% of the core genome. While we could not perform COG enrichment analysis due to the diverse array of categories identified, most of the genes under selection are related to metabolism or are membrane-associated. This is unsurprising given that membrane-associated proteins are the most likely to encounter selective pressures due to their contact with the environment [70]. This pattern has been observed in multiple studies of other Gram-positive organisms: in *Staphylococcus*, a genome-wide screen of 14 livestock-associated isolates found 60 genes under positive selection that were largely linked to metabolism (COG categories P, ion transport and metabolism; E, amino acid transport and metabolism; C, energy production and conversion) [71]. In *Bacillus* species, studies have shown that between 5 and 10% of the core genome is under positive selection; most genes under selection are linked to metabolism [17,72]. Only 0.38% of core genes in *Pseudomonas aeruginosa* were under selection, and most genes were either proteases and hydrolases, transporters or associated with DNA stabilization and replication [18]. While this comparison focuses on the overall proportion of the genome inferred to be under positive selection, variation in the methods and statistical thresholds used to detect selection across studies may contribute to differences in the number of genes identified.

Of the 16 genes PAML identified as under selection, 1 of them was not supported by omegaMap, and a further 5 were hypothetical genes. The genes *cysG* and *sirC* catalyse the first two reactions in the three-step siroheme biosynthesis pathway (as a uroporphyrinogen III methyltransferase and precorrin-2 dehydrogenase, respectively). Mutagenesis studies of *sirC* have shown that manipulating a number of residues significantly increased the activity of the enzyme [73]. Siroheme biosynthesis is distributed across bacteria, archaea, fungi and plants [74] and enables the assimilation of oxidized nitrogen and sulphur, acting as a cofactor for a range of enzymes, many of which are crucial for the biosynthesis of amino acids [75].

The genes *csd* and *yfcA* are both involved in the movement of sulphur between biomolecules (Csd is a cysteine desulphurase, while YfcA is a putative sulphur transporter belonging to the 4-toluene sulphonate uptake permease (TSUP) family). *csd* is involved in several different pathways, including the formation of iron–sulphur (Fe–S) clusters and protection from oxidative stress and has recently been characterized in *S. aureus*; it is thought *csd*-like genes could be potential therapeutic targets [76,77]. TSUP proteins are poorly characterized; however, TSUP homologues from *S. aureus* largely cluster as either Fe–S assembly proteins or transporters of sulphur-containing molecules [78].

Signals of positive selection were also identified in *nasD* (*nirB*), the gene encoding the large subunit of the nitrite reductase enzyme NirBD. NirBD requires both siroheme and Fe–S clusters as cofactors [79] and converts nitrite (NO_3_^-^) into ammonia as a means of detoxifying the environment [80]. It has been previously shown that high concentrations of nitrite derivatives can disrupt *S. epidermidis* biofilm formation, one of *S. epidermidis*’ key virulence factors [81,82]. Amongst the genes identified as positively selected, 29% were associated with siroheme biosynthesis, sulphur transport and Fe–S cluster formation, pathways that converge to provide essential cofactors for the subunit of a single enzyme, NirB, highlighting a striking signal of selection that warrants further investigation (Fig. S4).

Notably, yet another enzyme subunit linked to amino acid biosynthesis, HisI, was found to be under positive selection. *hisIE* encodes a bifunctional enzyme which catalyses the second and third steps of the histidine biosynthesis pathway [83,84]. Histidine biosynthesis plays an important role in bacterial metabolism; however, it has also been linked to pathogenesis. In *Acinetobacter baumannii,* it was found that extracellular histidine promoted pathogenesis, while in *M. tuberculosis, de novo* synthesis of histidine counteracted host upregulation of histidine-catabolizing enzymes [85,86].

Most genes linked to antimicrobial resistance or niche adaptation in *S. epidermidis* were not investigated in this study, as the majority of them are accessory genes. Despite this, four of the genes found to be under positive selection can also be linked to antimicrobial resistance. Mutations in the *ileS* gene, which encodes an isoleucyl-tRNA synthetase (IleRS), have been extensively linked to mupirocin resistance in staphylococci [87,90]. Mupirocin is a topical antibiotic typically used to decolonize patients of methicillin-resistant *S. aureus* (MRSA) by targeting bacterial IleRS [90]. In *S. aureus*, it was found that a single residue change from valine to phenylalanine at codon 588 (which we have identified as under positive selection) resulted in low-level resistance to mupirocin and did not incur a large fitness cost [88]. Unsurprisingly, widespread use of mupirocin has resulted in increased resistance in both *S. aureus* and *S. epidermidis* since they colonize similar body sites. However, this highlights the need to look for alternative antimicrobial agents to decolonize MRSA or employ stricter antimicrobial stewardship. In addition, the use of mupirocin has been shown to enhance biofilm production in *S. epidermidis*, which serves as its main virulence mechanism [91,92]. Although ColabFold and ChimeraX did not predict structural alterations in the proteins encoded by any of the genes under selection, the detection of loci such as *ileS*, which has a well-established adaptive role, as under positive selection, highlights the biological relevance of the signals identified here. Analysis of protein stability using FoldX did, however, identify multiple mutations predicted to influence protein stability. Different studies apply varying ΔΔG thresholds when classifying stability effects, with destabilizing mutations commonly defined by ΔΔG values of ~1.5–2.5 kcal mol^−1^ and stabilizing mutations by values below −0.5 kcal mol^−1^ [93,94]. Here, we adopted a more conservative ΔΔG threshold of ≥2.5 kcal mol^−1^ to classify mutations as destabilizing and ≤–0.5 kcal mol^−1^ to classify mutations as stabilizing. Sixteen mutations across six genes were predicted to have stabilizing effects on protein structure, including all four mutations identified in *sirC*. In contrast, four mutations across three genes, including the V588F mutation in *ileS*, were predicted to be destabilizing. As this mutation in *ileS* is known to confer resistance to mupirocin, this may reflect a trade-off between antibiotic resistance and protein stability.

*rluD_3* encodes a pseudouridine synthase responsible for converting uridine to pseudouridine in 23S rRNA; mutations in this gene have previously been associated with resistance to aminoglycosides [95,97]. *ndhB* encodes one of the subunits of the type I NADH dehydrogenase, which forms part of the electron transport chain and is critical for bacterial metabolism [98,99]. Mutations in NADH dehydrogenase genes have been linked to resistance against aminoglycosides due to the requirement of proton-motive force needed for uptake of the antibiotic [100]. It has also been shown that mutations in the *ndh* genes were linked to isoniazid resistance in *Mycobacterium tuberculosis*, where it is used as a treatment for both active and latent TB infections [101].

*yheS* encodes a putative ATP-binding cassette F (ABC-F) protein; these proteins are associated with antibiotic resistance, typically mediated through ribosomal protection where the ABC-F protein displaces the antibiotic at the ribosome binding site [102,103]. ABC-F proteins are found in a wide array of bacterial species, with a number of them having been characterized in staphylococci [104,107].

There was a strong association between minor alleles and clade. For *ileS*, *rluD_3* and *yheS* described above, most of these minor alleles were found in clade A. This is especially true for *ileS*, where the mutation was almost solely found in GC5 strains, which is consistent with earlier studies indicating that GC5 isolates are more hospital-adapted [7,12]. This selective pressure towards antimicrobial resistance amongst hospital-adapted isolates also highlights the importance of monitoring antibiotic efficacy in *S. epidermidis* isolates. For the remaining genes, there was a clear association between the minor alleles and clade B/GC4. These genes are associated with key metabolic processes that are well understood; however, it would appear that mutations in these genes are being driven by less-evident selection pressures. Clade B isolates include both commensal strains and those from more unique environmental niches; however, further study is needed to identify the role selection plays within this clade.

Comparison of the genes identified here as being under positive selection with those highlighted in the previous exploratory scan for balancing selection by Zhang *et al*. [21] revealed no overlap. This is not unexpected, as the exploratory scan focused on the top 1.3% of Tajima’s *D* values, whereas signatures of positive selection are typically associated with negative Tajima’s *D* values due to an excess of rare alleles following selective sweeps [108].

### Selection at the branch-site level

Using branch-site models to identify genes under selection along branches between clades A and B was more challenging compared to the site models. With site models highlighting a few key sites under selection, repeating the same analysis using only four foreground branches posed a new challenge, since the proportion of sites under selection would most likely be extremely low and so the signal present to be detected by PAML and HyPhy was also much lower than during the site model tests. This was demonstrated by the BEB test in the PAML branch-site models; the BEB test was only able to highlight specific sites in one of the three genes under selection. Zhang *et al*. [54] observed this issue in simulations of the PAML branch-site models; the power of the BEB test was low, and it frequently provided no sites under selection with a posterior probability of >95% despite the branch-site model supporting selection on foreground branches. More recently, Álvarez-Carretero *et al*. [64] discussed how foreground branches in PAML branch-site models should be determined *a priori*; otherwise, multiple correction testing is needed, while Bonferroni correction is too conservative for this type of analysis.

A similar issue is seen with branch-site models from HyPhy. Kosakovsky-Pond *et al*. [109] described how the proportion of sites under selection required to detect episodic selection along foreground branches is very high, ~10–15% for a gene even with inflated *ω* values of at least 4 or 5. However, when *ω*=2, with the same proportion of sites under selection, the power of the branch-site model used dropped to 8%, which points to the fact that recognizing a weak selection signal is much more difficult, even with a high proportion of sites under selection [109]. Therefore, as the site model with PAML only identified a maximum of five sites under selection for any of the tested genes, given the low power of the branch-site model in the simulations described above, at the branch level, it is difficult to detect a strong signal of selection. In addition, when testing datasets of viral pathogens using aBSREL, it was found that because of the large number of tests involved, genes that showed significant support (uncorrected *P*-value<0.05) for selection along foreground branches rarely survived multiple correction testing [58]. This demonstrates that even with a large cohort of samples, it is still challenging to reach the significance threshold with branch-level data, as shown with both our PAML and HyPhy results. Given that none of the genes identified under branch-site analyses were significant by both PAML and either aBSREL or BUSTED post-multiple-test correction, this suggests that the signal of selection in these genes is relatively weak and should, therefore, be interpreted with caution. Given that HyPhy methods detect different patterns of positive selection at the site, branch and gene levels, we applied multiple complementary tests (aBSREL, BUSTED and MEME). The limited overlap amongst methods likely reflects both the weak signal of selection and methodological differences rather than contradictory signals of selection.

*rhtC* was identified as under selection along the branches separating clades A and B, from the HyPhy branch-site analysis. Interestingly, while both the BEB test from PAML and MEME identified codon 94 under selection, the only observed change was a synonymous mutation of AGT to TCT (serine). This mutation was also almost exclusively observed in clade C strains; one clade B strain also had this alternative codon, but was the closest strain to clade C on the tree. *rhtC* encodes a threonine efflux pump which has been shown to confer resistance to a variety of amino acid analogues [110,111]. While the mutation in *rhtC* is synonymous, specific patterns of codon usage can impact both the speed and accuracy of translation and, therefore, affect fitness [112,114].

### Limitations and conclusion

While isolates from North America and Europe are well represented in our dataset, there are few from Central and South America, Africa and Asia. As this work is based upon a ‘global’ phylogeny of *S. epidermidis*, important lineages could be missing, biassing the selection analysis towards western countries, despite featuring all of the major clades previously identified; in *Escherichia coli*, lineages characterized as recombinant were later more accurately defined when using a larger, more diverse set of isolates [115]. Insufficient isolates from GC2 were available to accurately capture information about the role selection has played in this cluster, although this is unsurprising given the rarity of isolates. However, this should not have biassed the branch-site analysis, given that there were virtually even numbers of strains between clades A and B, while GC2 and GC4 were closely related, collapsing into a single group by STRUCTURE.

Screening the entire 1,003-isolate dataset for selection was not feasible due to the computing power required to run the analyses, particularly PAML. Initially, a larger subset of 200 strains was tested with PAML to estimate runtime; however, this equated to roughly 19 h per gene and was not feasible. Even though this was mitigated by sub-setting the isolates based on the phylogeny and using the alignment data for the whole dataset when analysing allelic diversity, it is still possible that not all genes under positive selection were identified.

Understanding the level of positive selection across a core genome can reveal the selection pressures that have defined the evolution of a species. The candidate genes we have identified are typically related to core metabolic pathways or associated with antimicrobial resistance, which highlights that different selective pressures are driving natural selection in the three clades within the population. In addition to the characterized genes discussed above, signatures of selection were also detected in several hypothetical genes, highlighting targets for future functional and evolutionary studies to determine how these variants may influence protein function and bacterial fitness.

## Supplementary material

10.1099/mgen.0.001692 Uncited Supplementary Material 1.

## Abbreviations

ABC-F: ATP-binding cassette F
ANI: average nucleotide identity
BEB: Bayes empirical Bayes
Fe–S: iron–sulphur
GCs: genetic clusters
HPC: high-performance computing cluster
IleRS: isoleucyl-tRNA synthetase
LRT: likelihood-ratio test
MRSA: methicillin-resistant *Staphylococcus aureus*
MSAs: multiple sequence alignments
STs: sequence types
TSB: trypticase soy broth
TSUP: 4-toluene sulphonate uptake permease
WGSs: whole-genome sequences
