# Supplementary material for: Screen for signatures of positive selection in the core genome of Staphylococcus epidermidis
Source: Microb Genom. 2026 Apr 20;12(4):001692. doi: 10.1099/mgen.0.001692 (PMC13095176; doi:10.1099/mgen.0.001692)
Supplement: Uncited Supplementary Material 1. [file mgen-12-01692-s001.pdf]

Supplementary material

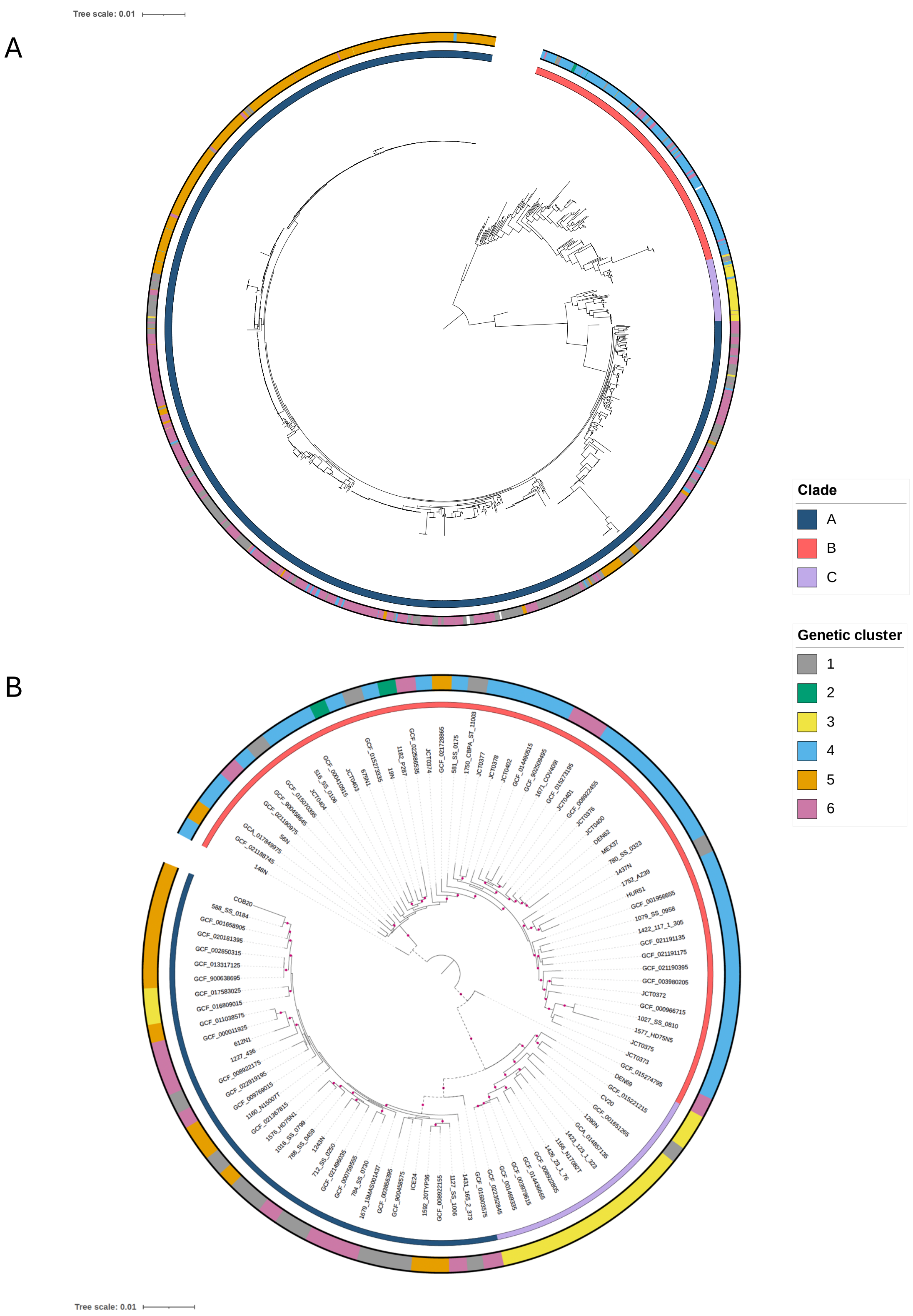

Figure S1. Core gene phylogenies for the dataset. Inner and outer rings represent clade and genetic clusters, respectively. Tree scales refer to substitutions per site. **(A)** Maximum-likelihood tree based on 467 core genes for all 1,003 strains. **(B)** Maximum-likelihood tree for the 100-strain subset, based on 826 core genes. Purple circles at nodes represent bootstrap support of at least 75%. Branches with dashed lines were selected for branch-site analysis.

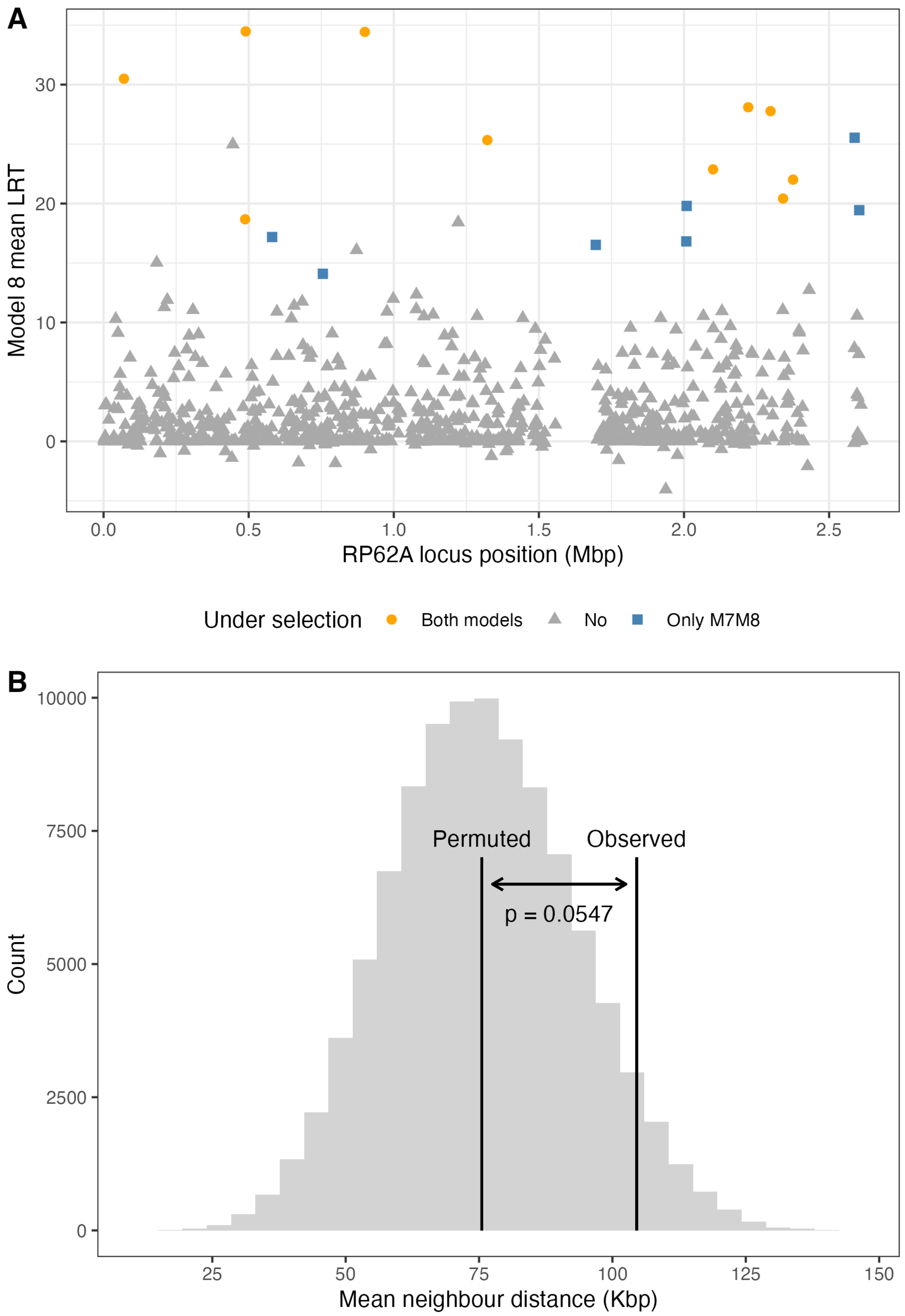

Figure S2. Clustering analysis for loci under site-level selection. **(A)** Distribution of likelihood ratio test scores from PAML models M7M8 for all 826 core genes, mapped to the RP62A genome. **(B)** Permutation test results comparing the mean distance to the nearest locus between genes under selection (observed) and all remaining loci (permuted) over 100,000 permutations.

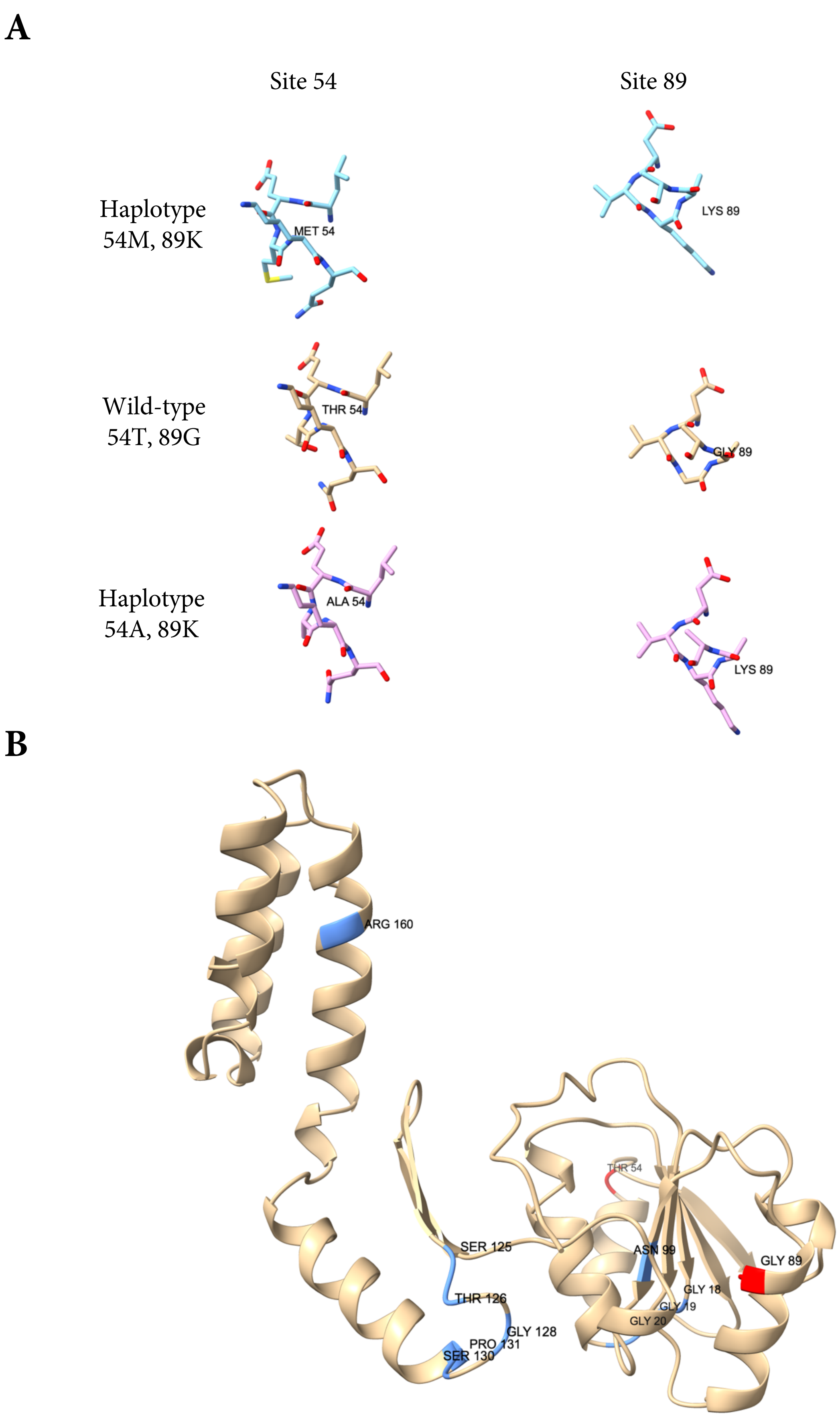

Figure S3. Alignment of *sirC* protein structures with ChimeraX. **(A)** Comparisons between sites 54 and 89 for the wild-type, and haplotypes screened for structural differences (top and bottom; RMSD values of 0.473 and 0.511, respectively). **(B)** Protein structure for wild-type *sirC*. Residues under selection are coloured red, while sites of functional importance are coloured blue (Schubert *et al.*, 2008).

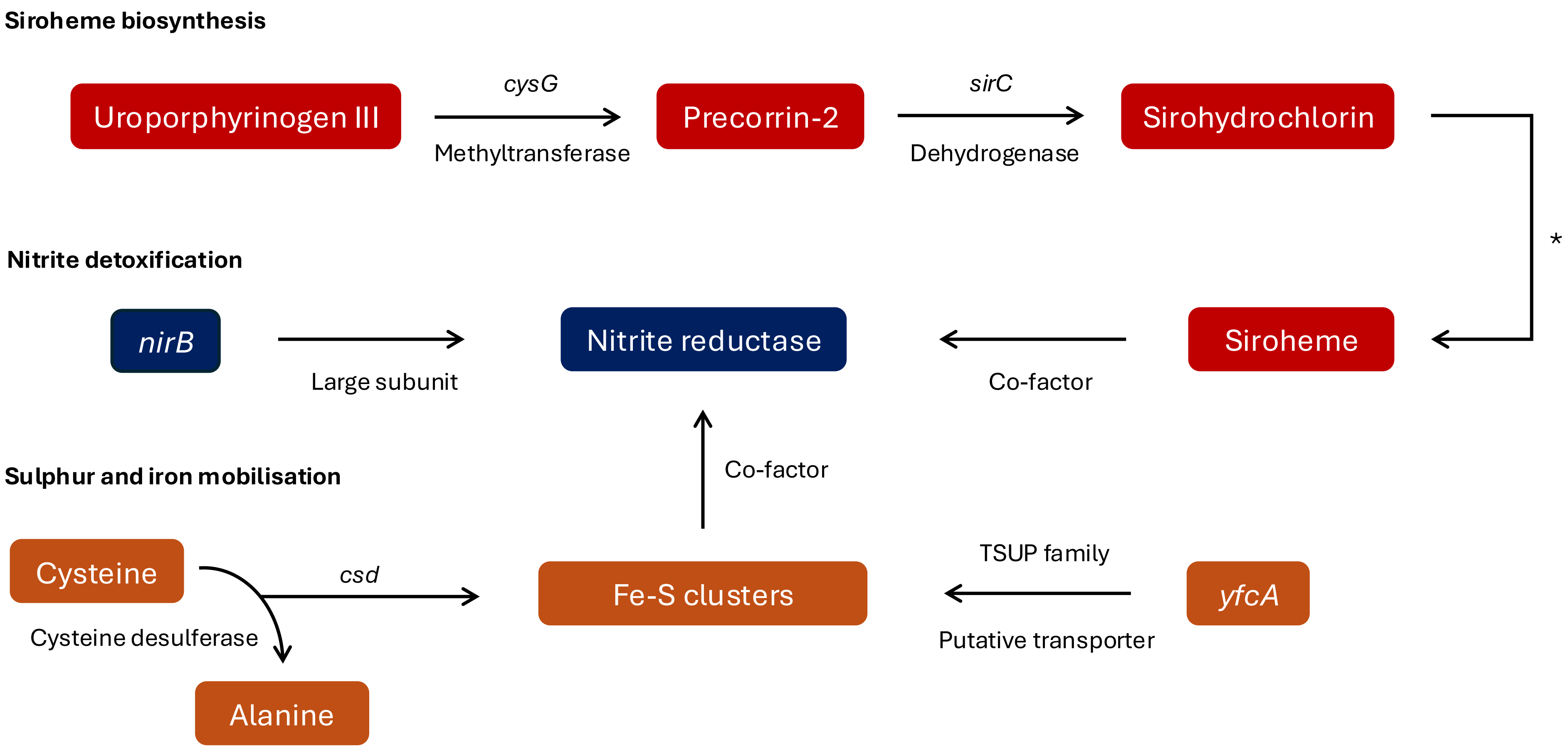

Figure S4. Flowchart of pathways with genes under site-level selection converging on the enzyme nitrite reductase. * = not under selection.

Table S1. Metadata for the genomes used in this study.

Genome	Source	Accession number	Strain name	Country	Year of isolation	Isolation information	ST	GC	Genomic group	Genome size (Mb)	N50 (kb)	Contig no.	Completeness	Contamination	ANI compared to type strain	100 strain subset
AGT24	Meric et al., 2015		NA	Argentina	1997	Blood	23	5	A	2.44	119	81	99.81	0	99.13	No
GCF_000308395	NCBI SSB		NA	Australia	Unknown	intravascular catheter	59	6	A	2.41	82	50	99.81	0.33	99.53	No
GCF_015273045	NCBI SSB		NA	Australia	Unknown	skin swab1639 from squamous cell	2	5	A	2.37	171.31	29	99.81	0.06	99.55	No
GCF_015273085	NCBI SSB		NA	Australia	Unknown	skin swab1639 from squamous cell	57	6	A	2.37	157.15	32	99.81	0.06	99.63	No
GCF_015273105	NCBI SSB		NA	Australia	Unknown	skin swab1639 from squamous cell	57	6	A	2.37	156.88	31	99.81	0.06	99.61	No
GCF_015273135	NCBI SSB		NA	Australia	Unknown	skin swab1639 from squamous cell	57	6	A	2.37	171.45	30	99.81	0.06	99.60	No
GCF_015273145	NCBI SSB		NA	Australia	Unknown	skin swab1639 from squamous cell	57	6	A	2.38	155.96	34	99.81	0.06	99.57	No
GCF_015273175	NCBI SSB		NA	Australia	Unknown	skin swab1617 from squamous cell	57	6	B	2.52	252.31	27	99.39	0	96.84	No
GCF_015273185	NCBI SSB		NA	Australia	Unknown	skin swab1639 from squamous cell	679	4	A	2.38	152.77	33	99.81	0.06	99.59	No
GCF_015273195	NCBI SSB		NA	Australia	Unknown	skin swab1639 from squamous cell	57	6	B	2.55	210.57	26	99.67	0.42	96.89	Yes
GCF_015273235	NCBI SSB		NA	Australia	Unknown	skin swab1617 from squamous cell	558	4	B	2.52	331.10	22	99.39	0	96.82	No
GCF_015273245	NCBI SSB		NA	Australia	Unknown	skin swab1617 from squamous cell	679	4	B	2.52	331.01	24	99.39	0	96.80	No
GCF_015273275	NCBI SSB		NA	Australia	Unknown	skin swab1617 from squamous cell	679	4	B	2.52	331.24	23	99.39	0	96.84	No
GCF_015273285	NCBI SSB		NA	Australia	Unknown	skin swab1617 from squamous cell	679	4	A	2.38	156.38	32	99.81	0	99.48	No
GCF_015273295	NCBI SSB		NA	Australia	Unknown	skin swab1617 from squamous cell	487	6	A	2.43	167.70	24	99.81	0.28	99.41	No
GCF_015273335	NCBI SSB		NA	Australia	Unknown	skin swab1599 from squamous cell	1152	1	B	2.56	643.10	26	99.45	0.03	96.83	Yes
GCF_015273345	NCBI SSB		NA	Australia	Unknown	skin swab1599 from squamous cell	1091	4	B	2.47	217.56	28	99.67	0	96.85	No
GCF_015273365	NCBI SSB		NA	Australia	Unknown	skin swab1574 from squamous cell	987	4	A	2.47	167.34	27	99.81	0	99.53	No
GCF_015273385	NCBI SSB		NA	Australia	Unknown	skin swab1574 from squamous cell	110	6	A	2.47	167.05	30	99.81	0	99.55	No
GCF_015273415	NCBI SSB		NA	Australia	Unknown	skin swab1574 from squamous cell	110	6	A	2.47	199.05	28	99.81	0	99.52	No
GCF_015273495	NCBI SSB		NA	Australia	Unknown	skin swab1561 from squamous cell	110	6	A	2.36	166.01	31	99.81	0.28	99.49	No
GCF_015273505	NCBI SSB		NA	Australia	Unknown	skin swab1561 from squamous cell	35	1	A	2.37	205.32	23	99.81	0.28	99.44	No
GCF_015273525	NCBI SSB		NA	Australia	Unknown	skin swab1561 from squamous cell	35	1	A	2.37	209.74	23	99.81	0.28	99.46	No
GCF_015273535	NCBI SSB		NA	Australia	Unknown	skin swab1561 from squamous cell	35	1	A	2.37	209.87	22	99.81	0.28	99.47	No
GCF_015273575	NCBI SSB		NA	Australia	Unknown	skin swab1561 from squamous cell	35	1	A	2.37	209.87	23	99.81	0.28	99.41	No
GCF_015273585	NCBI SSB		NA	Australia	Unknown	skin swab1556 from squamous cell	35	1	A	2.45	86.31	53	99.81	0	99.24	No
GCF_015273595	NCBI SSB		NA	Australia	Unknown	skin swab1561 from squamous cell	20	1	A	2.37	209.87	22	99.81	0.28	99.45	No
GCF_015273605	NCBI SSB		NA	Australia	Unknown	skin swab1556 from squamous cell	35	1	A	2.42	156.57	34	99.81	0	99.52	No
GCF_015273655	NCBI SSB		NA	Australia	Unknown	skin swab1556 from squamous cell	59	6	A	2.46	107.01	52	99.81	0	99.18	No
GCF_015273665	NCBI SSB		NA	Australia	Unknown	skin swab1556 from squamous cell	20	1	A	2.45	83.03	59	99.81	0	99.18	No
GCF_015273735	NCBI SSB		NA	Australia	Unknown	skin swab1532 from squamous cell	20	1	B	2.54	245.03	24	99.67	0.28	96.80	No
GCF_015273785	NCBI SSB		NA	Australia	Unknown	skin swab1532 from squamous cell	558	4	A	2.43	232.30	31	99.81	0.09	99.62	No
GCF_015273805	NCBI SSB		NA	Australia	Unknown	skin swab1532 from squamous cell	59	6	B	2.58	193.95	33	99.67	0.42	96.80	No
GCF_015273815	NCBI SSB		NA	Australia	Unknown	skin swab1532 from squamous cell	290	4	B	2.62	132.42	34	99.67	0.08	96.90	No
GCF_015273855	NCBI SSB		NA	Australia	Unknown	skin swab1483 from squamous cell	640	4	A	2.47	109.93	46	99.81	0	99.35	No
GCF_015273865	NCBI SSB		NA	Australia	Unknown	skin swab1483 from squamous cell	59	6	A	2.77	87.69	61	99.81	0	99.15	No
GCF_015273875	NCBI SSB		NA	Australia	Unknown	skin swab1483 from squamous cell	2	5	A	2.49	121.09	37	99.81	0	99.46	No
GCF_015273895	NCBI SSB		NA	Australia	Unknown	skin swab1483 from squamous cell	153	6	A	2.44	121.09	38	99.81	0	99.45	No
GCF_015274155	NCBI SSB		NA	Australia	Unknown	skin swab1470 from squamous cell	153	6	A	2.41	210.82	30	99.81	0	99.52	No
GCF_015274195	NCBI SSB		NA	Australia	Unknown	skin swab1442 from squamous cell	59	6	A	2.43	135.62	28	99.81	0	99.54	No
GCF_015274235	NCBI SSB		NA	Australia	Unknown	skin swab1442 from squamous cell	59	6	A	2.43	126.29	28	99.81	0	99.50	No
GCF_015274255	NCBI SSB		NA	Australia	Unknown	skin swab1442 from squamous cell	59	6	A	2.43	210.08	27	99.81	0	99.55	No
GCF_015274265	NCBI SSB		NA	Australia	Unknown	skin swab1442 from squamous cell	59	6	A	2.43	126.88	29	99.81	0	99.54	No
GCF_015274275	NCBI SSB		NA	Australia	Unknown	skin swab1391 from squamous cell	59	6	B	2.54	199.73	34	99.67	0	96.91	No
GCF_015274285	NCBI SSB		NA	Australia	Unknown	skin swab1391 from squamous cell	1153	4	B	2.53	125.40	41	99.67	0	96.83	No
GCF_015274325	NCBI SSB		NA	Australia	Unknown	skin swab1391 from squamous cell	1153	4	B	2.53	125.58	44	99.67	0	96.85	No
GCF_015274355	NCBI SSB		NA	Australia	Unknown	skin swab1391 from squamous cell	1153	4	B	2.53	155.77	38	99.67	0	96.89	No
GCF_015274385	NCBI SSB		NA	Australia	Unknown	skin swab1389 from squamous cell	1153	4	B	2.52	199.60	27	99.67	0	96.94	No
GCF_015274425	NCBI SSB		NA	Australia	Unknown	skin swab1389 from squamous cell	558	4	B	2.52	167.83	28	99.67	0	96.91	No
GCF_015274455	NCBI SSB		NA	Australia	Unknown	skin swab1385 from squamous cell	558	4	B	2.52	125.40	38	99.67	0	96.91	No
GCF_015274475	NCBI SSB		NA	Australia	Unknown	skin swab1389 from squamous cell	1153	4	A	2.45	126.26	31	99.81	0	99.50	No
GCF_015274485	NCBI SSB		NA	Australia	Unknown	skin swab1385 from squamous cell	59	6	B	2.52	143.79	37	99.67	0	96.89	No
GCF_015274505	NCBI SSB		NA	Australia	Unknown	skin swab1385 from squamous cell	1153	4	B	2.52	199.80	29	99.67	0	96.95	No
GCF_015274555	NCBI SSB		NA	Australia	Unknown	skin swab1385 from squamous cell	558	4	B	2.52	151.62	36	99.67	0	96.90	No
GCF_015274575	NCBI SSB		NA	Australia	Unknown	skin swab1383 from squamous cell	1153	4	A	2.43	161.88	30	99.81	0	99.54	No
GCF_015274615	NCBI SSB		NA	Australia	Unknown	skin swab1383 from squamous cell	59	6	A	2.42	161.62	29	99.81	0	99.55	No
GCF_015274625	NCBI SSB		NA	Australia	Unknown	skin swab1383 from squamous cell	59	6	A	2.43	161.85	32	99.81	0	99.55	No
GCF_015274735	NCBI SSB		NA	Australia	Unknown	skin swab1381 from squamous cell	59	6	A	2.43	161.82	28	99.81	0	99.51	No
GCF_015274775	NCBI SSB		NA	Australia	Unknown	skin swab1378 from squamous cell	59	6	B	2.56	180.60	37	99.67	0	96.95	No
GCF_015274785	NCBI SSB		NA	Australia	Unknown	skin swab1381 from squamous cell	332	4	A	2.45	192.08	32	99.81	0	99.57	No
GCF_015274795	NCBI SSB		NA	Australia	Unknown	skin swab1378 from squamous cell	59	6	B	2.56	138.26	42	99.67	0.19	97.56	Yes
GCF_015274835	NCBI SSB		NA	Australia	Unknown	skin swab1378 from squamous cell	672	4	B	2.53	171.34	37	99.67	0	96.87	No
GCF_015274905	NCBI SSB		NA	Australia	Unknown	skin swab1378 from squamous cell	332	4	B	2.56	203.70	38	99.67	0	96.92	No
GCF_022832895	NCBI SSB		NA	Australia	Unknown	pertoneal cavity	226	4	A	2.54	2490.70	6	99.81	0	99.40	No
GCF_022832915	NCBI SSB		NA	Australia	Unknown	pertoneal cavity	844	6	A	2.54	2489.17	7	99.81	0	99.35	No
GCF_022832935	NCBI SSB		NA	Australia	Unknown	pertoneal cavity	844	6	A	2.54	2489.41	7	99.81	0	99.39	No
GCF_022585795	NCBI SSB		NA	Austria	Unknown	skin	1171	6	A	2.53	147.47	35	99.81	0.09	99.17	No
GCF_022586395	NCBI SSB		NA	Austria	Unknown	skin	10	6	A	2.47	201.41	25	99.81	0	99.54	No
GCF_022586415	NCBI SSB		NA	Austria	Unknown	skin	59	6	A	2.53	147.47	36	99.81	0.09	99.09	No
GCF_022586435	NCBI SSB		NA	Austria	Unknown	skin	10	6	A	2.42	145.99	30	99.53	0	99.40	No
GCF_022586445	NCBI SSB		NA	Austria	Unknown	skin	1172	6	A	2.44	123.33	33	99.81	0	99.48	No
GCF_022586475	NCBI SSB		NA	Austria	Unknown	skin	59	6	A	2.46	165.57	28	99.81	0.02	99.56	No
GCF_022586495	NCBI SSB		NA	Austria	Unknown	skin	5	6	A	2.48	119.59	33	99.81	0.58	99.57	No
GCF_022586515	NCBI SSB		NA	Austria	Unknown	skin	5	6	A	2.46	204.80	26	99.81	0	99.42	No
GCF_022586535	NCBI SSB		NA	Austria	Unknown	skin	210	6	B	2.54	281.08	22	99.67	0.03	96.88	Yes
GCF_022586555	NCBI SSB		NA	Austria	Unknown	skin	640	4	A	2.43	204.79	31	99.81	0	99.41	No
GCF_022586575	NCBI SSB		NA	Austria	Unknown	skin	210	6	A	2.55	144.36	42	99.25	0.02	99.46	No
GCF_022586595	NCBI SSB		NA	Austria	Unknown	skin	5	6	A	2.55	144.36	40	99.81	0.02	99.49	No
GCF_000685135	NCBI SSB		NA	Belgium	Unknown	endotracheal tube biofilm	35	1	A	2.35	76	102	99.81	0	99.56	No
GCF_001298115	NCBI SSB		NA	Belgium	Unknown	skin	184	1	A	2.48	137	42	99.81	0	99.50	No
GCF_005760475	NCBI SSB		NA	Belgium	Unknown	Femur	59	6	A	2.43	71	126	99.81	0	99.47	No
GCF_005760495	NCBI SSB		NA	Belgium	Unknown	Femur	83	6	A	2.52	61	116	99.81	0.23	99.30	No
GCF_021366715	NCBI SSB		NA	Belgium	Unknown	milk samples	769	1	A	2.48	86.92	90	99.81	0.28	99.48	No
GCF_021366835	NCBI SSB		NA	Belgium	Unknown	milk samples	100	6	A	2.49	119.55	55	99.81	0.37	99.40	No
GCF_021366885	NCBI SSB		NA	Belgium	Unknown	milk samples	1166	6	A	2.53	110.72	58	99.81	0.28	99.44	No
GCF_021367085	NCBI SSB		NA	Belgium	Unknown	milk samples	1166	6	A	2.40	133.49	52	99.81	0	99.53	No

GCF_021384785	NCBI SSB	NA	Belgium	Unknown	milk sample	100	6	A	2.44	163.66	46	99.81	0	99.56	No
1671_COV409I	PubMLST	NA	Brazil	2020	Hospital tracheal aspirate	1002	6	B	2.51	101.25	75	99.67	0	97.10	Yes
GCF_002799425	NCBI SSB	NA	Brazil	Unknown	blood	2	5	A	2.73	82	63	98.21	0.05	99.26	No
GCF_002799485	NCBI SSB	NA	Brazil	Unknown	blood	2	5	A	2.76	126	70	99.81	0.31	99.29	No
GCF_002799505	NCBI SSB	NA	Brazil	Unknown	blood	2	5	A	2.69	102	56	99.19	0.05	99.36	No
GCF_002799565	NCBI SSB	NA	Brazil	Unknown	blood	2	5	A	2.68	91	57	99.81	0	99.34	No
BUG37	Meric et al., 2015	NA	Bulgaria	1997	Wound	19	3	C	2.51	297	55	99.67	0	98.20	No
BUG46	Meric et al., 2015	NA	Bulgaria	1997	n.a.	189	1	A	2.45	170	59	99.81	0	99.54	No
1749_CBPA_ST_10002	PubMLST	NA	Canada	2010	Human blood	1072	1	A	2.47	2439.28	4	99.81	0	99.27	No
1750_CBPA_ST_11003	PubMLST	NA	Canada	2011	Human blood	16	1	B	2.59	2567.00	2	99.67	0	96.88	Yes
1751_AZ22	PubMLST	NA	Canada	2015	Human skin swab	1174	4	A	2.48	2462.01	2	99.67	0.28	99.51	No
1752_AZ39	PubMLST	NA	Canada	2015	Human skin swab	73	1	B	2.51	2454.33	4	98.79	0	96.77	Yes
GCF_003035325	NCBI SSB	NA	Canada	Unknown	Herd 302	100	6	A	2.50	111	163	99.81	0.28	99.50	No
GCF_003035345	NCBI SSB	NA	Canada	Unknown	Herd 416	57	6	A	2.36	64	93	99.81	0	99.48	No
GCF_003035385	NCBI SSB	NA	Canada	Unknown	Herd 102	1053	6	A	2.43	56	119	99.63	0.47	99.49	No
GCF_003035685	NCBI SSB	NA	Canada	Unknown	Herd 225	59	6	A	2.36	52	78	99.81	0	99.61	No
GCF_003577845	NCBI SSB	NA	Canada	Unknown	Herd 213	57	6	A	2.47	197	97	99.81	0.17	99.50	No
GCF_003578055	NCBI SSB	NA	Canada	Unknown	Herd 308	59	6	A	2.47	82	88	99.81	0	99.46	No
GCF_003578065	NCBI SSB	NA	Canada	Unknown	Herd 202	100	6	A	2.49	185	56	99.81	0.09	99.50	No
CV13	Meric et al., 2015	NA	Cape Verde	1997	Nasal swab	369	4	B	2.44	184	59	99.67	0	96.99	No
CV20	Meric et al., 2015	NA	Cape Verde	1997	Nasal swab	72	1	C	2.49	231	35	99.67	0	98.42	Yes
CV28	Meric et al., 2015	NA	Cape Verde	1997	Nasal swab	44	6	A	2.53	128	65	99.81	1.24	99.49	No
CV45	Meric et al., 2015	NA	Cape Verde	1997	Nasal swab	23	5	A	2.46	135	63	99.81	0	99.08	No
GCF_009663955	NCBI SSB	NA	China	Unknown	soil from plant root system	307	6	A	2.51	183	27	99.81	0.56	99.48	No
COB20	Meric et al., 2015	NA	Columbia	1997	Urine	51	5	A	2.46	144	78	99.11	0	98.71	Yes
GCF_004323085	NCBI SSB	NA	Czech Republic	Unknown	Blood Culture	5	6	A	2.46	112	36	99.81	0	99.56	No
GCF_001412455	NCBI SSB	NA	Czechoslovakia	Unknown	nose	5	6	A	2.38	70	85	99.75	0	99.99	No
DEN107	Meric et al., 2015	NA	Denmark	1998	Blood	40	1	A	2.48	121	90	99.25	0.06	99.08	No
DEN110	Meric et al., 2015	NA	Denmark	1998	n.a.	66	4	B	2.53	165	70	99.67	0	96.83	No
DEN116	Meric et al., 2015	NA	Denmark	1998	Blood	215	1	A	2.49	144	76	99.81	0.28	99.36	No
DEN161	Meric et al., 2015	NA	Denmark	1998	Blood	35	1	A	2.45	154	62	99.81	0	99.31	No
DEN178	Meric et al., 2015	NA	Denmark	1998	Blood	5	6	A	2.54	96	122	99.81	0.66	99.51	No
DEN185	Meric et al., 2015	NA	Denmark	1998	Blood	21	6	A	2.56	119	76	99.81	0	99.47	No
DEN189	Meric et al., 2015	NA	Denmark	1998	n.a.	55	6	A	2.43	126	52	99.81	0	99.49	No
DEN19	Meric et al., 2015	NA	Denmark	1997	n.a.	1	1	A	2.52	91	100	99.81	0.09	99.40	No
DEN22	Meric et al., 2015	NA	Denmark	1997	Blood	4	6	A	2.50	76	93	99.81	0	99.43	No
DEN61	Meric et al., 2015	NA	Denmark	1997	Wound	22	5	A	2.44	137	76	99.81	0.02	99.46	No
DEN62	Meric et al., 2015	NA	Denmark	1997	Blood	230	4	B	2.57	195	85	99.67	0.06	96.83	Yes
DEN69	Meric et al., 2015	NA	Denmark	1997	Blood	56	3	C	2.48	205	63	99.67	0	98.27	Yes
DEN73	Meric et al., 2015	NA	Denmark	1997	Blood	21	6	A	2.57	119	86	99.81	0.66	99.52	No
DEN76	Meric et al., 2015	NA	Denmark	1997	Blood	14	1	A	2.41	123	69	99.75	0.3	99.43	No
GCF_001457235	NCBI SSB	NA	Denmark	Unknown	NA	184	1	A	2.43	85	61	99.81	0	99.55	No
GCF_001469315	NCBI SSB	NA	Denmark	Unknown	NA	73	1	A	2.49	2431	5	99.67	0.7	99.48	No
GCF_001469325	NCBI SSB	NA	Denmark	Unknown	skin	73	1	A	2.42	82	72	99.67	0.7	99.46	No
GCF_001469335	NCBI SSB	NA	Denmark	Unknown	NA	170	3	C	2.55	54	91	98.87	0.22	98.23	Yes
GCF_001956655	NCBI SSB	NA	Denmark	Unknown	NA	1018	4	B	2.61	2573	3	99.61	0.09	96.86	Yes
MCO150	Meric et al., 2015	NA	Denmark	1998	Blood	46	6	A	2.58	145	81	99.81	0	99.46	No
GCF_000304575	NCBI SSB	NA	France	Unknown	blood from a patient with	673	3	C	2.56	94	81	99.06	0.22	98.11	No
GCF_014436665	NCBI SSB	NA	France	Unknown	cheekbone	65	3	C	2.48	256	48	99.67	0	98.46	Yes
GCF_016406625	NCBI SSB	NA	France	Unknown	NA	329	4	A	2.60	2468.35	6	99.81	0	99.27	No
GCF_021484865	NCBI SSB	NA	France	Unknown	NA	59	6	A	2.43	2405.81	4	99.81	0	99.53	No
GCF_021496015	NCBI SSB	NA	France	Unknown	NA	153	6	A	2.40	2389.52	3	99.81	0	99.62	No
GCF_021496035	NCBI SSB	NA	France	Unknown	NA	218	1	A	2.59	2480.09	7	99.77	0	99.34	Yes
GCF_021496075	NCBI SSB	NA	France	Unknown	NA	1168	1	A	2.45	2439.44	2	99.81	0	99.50	No
GCF_021513035	NCBI SSB	NA	France	Unknown	NA	5	6	A	2.56	2465.75	5	99.67	0.06	99.45	No
GCF_021513055	NCBI SSB	NA	France	Unknown	NA	73	1	A	2.53	2502.35	4	99.81	0.59	99.48	No
GCF_021513075	NCBI SSB	NA	France	Unknown	NA	297	1	A	2.49	2435.35	4	99.67	0.06	99.43	No
GCF_021513095	NCBI SSB	NA	France	Unknown	NA	253	1	A	2.46	2384.55	8	99.81	0	99.60	No
GCF_021513115	NCBI SSB	NA	France	Unknown	NA	1169	6	A	2.52	2429.88	5	99.67	0.06	99.40	No
GCF_021728865	NCBI SSB	NA	France	Unknown	Chronic skin infection	2	5	B	2.52	133.98	51	99.67	0.06	96.81	Yes
1043_SS_0922	Meric et al., 2018	NA	Germany	2011	Hip endoprosthesis	5	6	A	2.40	96	65	99.25	0.28	99.50	No
1044_SS_0923	Meric et al., 2018	NA	Germany	2011	Knee endoprosthesis	2	5	A	2.68	74	114	99.81	0.28	99.36	No
1049_SS_0928	Meric et al., 2018	NA	Germany	2011	Knee endoprosthesis	2	5	A	2.67	83	97	99.81	0	99.37	No
1050_SS_0929	Meric et al., 2018	NA	Germany	2011	Femur endoprosthesis	48	5	A	2.40	117	83	99.67	0.09	99.33	No
1051_SS_0930	Meric et al., 2018	NA	Germany	2011	Hip endoprosthesis	2	5	A	2.53	119	88	99.81	0	99.42	No
1054_SS_0933	Meric et al., 2018	NA	Germany	2011	Knee endoprosthesis	23	5	A	2.45	115	74	99.81	0	99.09	No
1065_SS_0944	Meric et al., 2018	NA	Germany	2011	Osteomyelitis without implant, elbow	2	5	A	2.68	93	89	99.81	0.28	99.38	No
1066_SS_0945	Meric et al., 2018	NA	Germany	2011	Osteomyelitis without implant, tibia	73	1	A	2.45	204	63	99.67	0	99.45	No
1067_SS_0946	Meric et al., 2018	NA	Germany	2011	Knee endoprosthesis	5	6	A	2.53	119	67	99.81	0	99.46	No
1070_SS_0949	Meric et al., 2018	NA	Germany	2011	Osteomyelitis without implant, thoracal spine	7	6	A	2.49	142	62	99.81	0.58	99.44	No
1071_SS_0950	Meric et al., 2018	NA	Germany	2011	Osteomyelitis without implant, knee	2	5	A	2.70	83	92	99.81	0.28	99.38	No
1072_SS_0951	Meric et al., 2018	NA	Germany	2011	Fracture fixation, plate, calcaneus	23	5	A	2.46	102	82	99.81	0	99.06	No
1073_SS_0952	Meric et al., 2018	NA	Germany	2011	Fracture fixation, plate, tibia distal	5	6	A	2.44	96	83	99.81	0.28	99.52	No
1076_SS_0955	Meric et al., 2018	NA	Germany	2011	Fracture fixation, screw, upper ankle joint	73	1	A	2.54	132	75	99.67	0.37	99.48	No
1078_SS_0957	Meric et al., 2018	NA	Germany	2011	Fracture fixation, Ligament reconstruction device, femur	1004	4	B	2.43	83	96	99.67	0	96.88	No
1079_SS_0958	Meric et al., 2018	NA	Germany	2011	Fracture fixation, screw, tibia proximal	1005	4	B	2.49	102	73	99.65	0.14	96.77	Yes
1085_SS_0964	Meric et al., 2018	NA	Germany	2011	Fracture fixation, plate, tibia	10	6	A	2.46	58	134	99.81	0.09	99.24	No
1102_SS_0981	Meric et al., 2018	NA	Germany	2011	Fracture fixation, plate, radius distal	325	4	B	2.52	111	107	99.67	0	96.81	No
1103_SS_0982	Meric et al., 2018	NA	Germany	2011	Fracture fixation, Internal fixator, lumbar spine	130	6	A	2.44	98	84	99.81	0	99.47	No
1104_SS_0983	Meric et al., 2018	NA	Germany	2011	Fracture fixation, plate, tibia distal	2	5	A	2.67	71	101	99.81	0	99.41	No
1106_SS_0985	Meric et al., 2018	NA	Germany	2011	Fracture fixation, plate, tibia distal	2	5	A	2.68	106	78	99.81	0	99.42	No
1107_SS_0986	Meric et al., 2018	NA	Germany	2011	Fracture fixation, plate, tibia proximal	188	5	A	2.70	111	77	99.77	0	99.31	No
1110_SS_0989	Meric et al., 2018	NA	Germany	2011	Fracture fixation, plate, tibia midshaft	2	5	A	2.68	109	86	99.81	0	99.44	No
1113_SS_0992	Meric et al., 2018	NA	Germany	2011	Fracture fixation, plate, pelvis	1006	4	B	2.50	201	103	99.67	0	96.69	No
1122_SS_1001	Meric et al., 2018	NA	Germany	2011	Fracture fixation, plate, clavicle	1030	3	C	2.49	143	46	99.67	0.28	98.19	No
1123_SS_1002	Meric et al., 2018	NA	Germany	2011	Fracture fixation, plate, radius	5	6	A	2.51	83	92	99.81	0.09	99.51	No
1127_SS_1006	Meric et al., 2018	NA	Germany	2011	Fracture fixation,plate, tibia midshaft	467	6	A	2.46	71	98	99.67	0.28	99.40	Yes

1131_SS_1010	Meric et al., 2018	NA	Germany	2011	Fracture fixation, nail, tibia distal	2	5	A	2.66	122	84	99.81	0	99.35	No
1135_SS_1014	Meric et al., 2018	NA	Germany	2011	Fracture fixation, plate, tibia distal	87	5	A	2.47	99	78	99.81	0	99.40	No
1570_HD75_1	PubMLST	NA	Germany	2017	NA	984	4	B	2.62	217	51	99.67	0.3	96.95	No
1571_HD4N10	PubMLST	NA	Germany	2016	carrier	1034	6	A	2.47	103	81	99.81	0.28	99.86	No
1574_HD43N12	PubMLST	NA	Germany	2016	carrier	986	6	A	2.42	102	120	99.25	0.56	99.53	No
1575_HD59N12	PubMLST	NA	Germany	2016	carrier	987	4	B	2.49	182	68	99.67	0	96.88	No
1576_HD75N1	PubMLST	NA	Germany	2017	carrier	988	5	A	2.47	154	66	99.67	0	98.98	Yes
1577_HD75N5	PubMLST	NA	Germany	2017	carrier	723	4	B	2.56	422	59	99.67	0	97.63	Yes
1578_HD75N6	PubMLST	NA	Germany	2017	carrier	723	4	B	2.56	363	55	99.67	0	97.65	No
423_SS_0001	Meric et al., 2018	NA	Germany	2001	Joint synovial fluid (direct isolation)	171	3	C	2.50	1311	39	99.67	0	98.21	No
424_SS_0002	Meric et al., 2018	NA	Germany	2001	Intraoperative isolation	171	3	C	2.50	1350	43	99.67	0	98.22	No
426_SS_0004	Meric et al., 2018	NA	Germany	2001	Intraoperative isolation	6	1	A	2.48	112	75	99.25	0	99.54	No
429_SS_0007	Meric et al., 2018	NA	Germany	2001	Joint synovial fluid (direct isolation)	6	1	A	2.46	162	57	99.81	0	99.53	No
433_SS_0013	Meric et al., 2018	NA	Germany	2001	Joint synovial fluid (direct isolation)	2	5	A	2.52	102	92	99.81	0.09	99.41	No
434_SS_0014	Meric et al., 2018	NA	Germany	2001	Intraoperative isolation	2	5	A	2.47	124	93	99.81	0.14	99.48	No
443_SS_0025	Meric et al., 2018	NA	Germany	2001	Joint synovial fluid (direct isolation)	2	5	A	2.55	152	71	99.81	0	99.38	No
444_SS_0026	Meric et al., 2018	NA	Germany	2002	Intraoperative isolation	2	5	A	2.44	147	56	99.81	0	99.57	No
445_SS_0027	Meric et al., 2018	NA	Germany	2001	Joint synovial fluid (direct isolation)	59	6	A	2.46	126	84	99.7	0	99.49	No
446_SS_0028	Meric et al., 2018	NA	Germany	2002	Intraoperative isolation	59	6	A	2.46	126	84	99.81	0.11	99.44	No
447_SS_0029	Meric et al., 2018	NA	Germany	2001	Joint synovial fluid (direct isolation)	2	5	A	2.73	158	74	99.81	0	99.28	No
448_SS_0030	Meric et al., 2018	NA	Germany	2002	Intraoperative isolation	2	5	A	2.74	148	73	99.81	0	99.30	No
453_SS_0035	Meric et al., 2018	NA	Germany	2002	Joint synovial fluid (direct isolation)	23	5	A	2.52	131	82	99.81	0	98.98	No
454_SS_0036	Meric et al., 2018	NA	Germany	2002	Intraoperative isolation	23	5	A	2.52	131	86	99.81	0	99.04	No
457_SS_0039	Meric et al., 2018	NA	Germany	2001	Joint synovial fluid (direct isolation)	51	5	A	2.47	110	87	99.67	0.07	98.65	No
458_SS_0040	Meric et al., 2018	NA	Germany	2002	Intraoperative isolation	51	5	A	2.47	118	90	99.67	0.06	98.66	No
464_SS_0046	Meric et al., 2018	NA	Germany	2002	Intraoperative isolation	1035	1	A	2.44	110	76	99.67	0	98.93	No
467_SS_0051	Meric et al., 2018	NA	Germany	2002	Joint synovial fluid (direct isolation)	1036	3	C	2.55	351	40	99.67	0.37	97.98	No
468_SS_0052	Meric et al., 2018	NA	Germany	2002	Intraoperative isolation	1037	3	C	2.50	1298	36	99.67	0.34	98.01	No
469_SS_0053	Meric et al., 2018	NA	Germany	2002	Joint synovial fluid (direct isolation)	66	4	B	2.57	119	148	99.67	0	96.84	No
470_SS_0054	Meric et al., 2018	NA	Germany	2002	Intraoperative isolation	66	4	B	2.53	197	59	99.67	0	96.82	No
473_SS_0057	Meric et al., 2018	NA	Germany	2002	Joint synovial fluid (direct isolation)	59	6	A	2.46	111	67	99.81	0	99.53	No
474_SS_0058	Meric et al., 2018	NA	Germany	2002	Intraoperative isolation	59	6	A	2.46	126	81	99.81	0	99.49	No
479_SS_0063	Meric et al., 2018	NA	Germany	2002	Joint synovial fluid (direct isolation)	89	1	A	2.44	140	68	99.67	0	98.88	No
480_SS_0064	Meric et al., 2018	NA	Germany	2002	Intraoperative isolation	89	1	A	2.43	140	66	99.67	0	98.84	No
482_SS_0068	Meric et al., 2018	NA	Germany	2002	Intraoperative isolation	22	5	A	2.36	194	51	99.81	0.02	99.57	No
483_SS_0071	Meric et al., 2018	NA	Germany	2001	Joint synovial fluid (direct isolation)	217	6	A	2.44	242	56	99.81	0	99.26	No
484_SS_0072	Meric et al., 2018	NA	Germany	2002	Intraoperative isolation	217	6	A	2.42	147	65	99.81	0.19	99.28	No
485_SS_0073	Meric et al., 2018	NA	Germany	2002	Joint synovial fluid (direct isolation)	22	5	A	2.35	185	44	99.81	0.02	99.55	No
486_SS_0074	Meric et al., 2018	NA	Germany	2002	Intraoperative isolation	22	5	A	2.35	149	54	99.81	0.02	99.57	No
487_SS_0075	Meric et al., 2018	NA	Germany	2001	Joint synovial fluid (direct isolation)	5	6	A	2.47	119	77	99.81	0	99.56	No
488_SS_0076	Meric et al., 2018	NA	Germany	2002	Intraoperative isolation	5	6	A	2.47	119	80	99.81	0	99.54	No
498_SS_0086	Meric et al., 2018	NA	Germany	2002	Intraoperative isolation	5	6	A	2.47	121	57	99.81	0	99.58	No
502_SS_0090	Meric et al., 2018	NA	Germany	2002	Intraoperative isolation	89	1	A	2.45	140	70	99.67	0.08	98.88	No
503_SS_0091	Meric et al., 2018	NA	Germany	2002	Intraoperative isolation	5	6	A	2.49	110	72	99.81	0	99.47	No
504_SS_0092	Meric et al., 2018	NA	Germany	2002	Intraoperative isolation	5	6	A	2.47	102	70	99.81	0	99.53	No
507_SS_0095	Meric et al., 2018	NA	Germany	2002	Intraoperative isolation	2	5	A	2.49	144	69	99.81	0	99.44	No
508_SS_0096	Meric et al., 2018	NA	Germany	2002	Intraoperative isolation	2	5	A	2.55	144	73	99.81	0	99.33	No
509_SS_0097	Meric et al., 2018	NA	Germany	2002	Intraoperative isolation	5	6	A	2.51	119	71	99.81	0.02	99.54	No
510_SS_0098	Meric et al., 2018	NA	Germany	2002	Intraoperative isolation	5	6	A	2.51	121	54	99.81	0	99.52	No
511_SS_0099	Meric et al., 2018	NA	Germany	2002	Intraoperative isolation	2	5	A	2.58	167	61	99.81	0	99.54	No
512_SS_0100	Meric et al., 2018	NA	Germany	2002	Intraoperative isolation	2	5	A	2.64	147	74	99.81	0	99.40	No
513_SS_0103	Meric et al., 2018	NA	Germany	2002	Intraoperative isolation	22	5	A	2.59	117	69	99.7	0.09	99.48	No
514_SS_0104	Meric et al., 2018	NA	Germany	2002	Intraoperative isolation	22	5	A	2.59	186	66	99.81	0.09	99.47	No
515_SS_0105	Meric et al., 2018	NA	Germany	2002	Intraoperative isolation	897	4	B	2.57	314	43	99.67	0.06	96.89	No
516_SS_0106	Meric et al., 2018	NA	Germany	2002	Intraoperative isolation	897	4	B	2.55	724	35	99.67	0.06	96.85	Yes
517_SS_0107	Meric et al., 2018	NA	Germany	2002	Intraoperative isolation	21	6	A	2.67	118	82	99.81	0	99.46	No
518_SS_0108	Meric et al., 2018	NA	Germany	2002	Intraoperative isolation	21	6	A	2.66	84	98	99.81	0.3	99.43	No
521_SS_0111	Meric et al., 2018	NA	Germany	2002	Joint synovial fluid (direct isolation)	2	5	A	2.62	147	64	99.81	0	99.55	No
522_SS_0112	Meric et al., 2018	NA	Germany	2002	Intraoperative isolation	2	5	A	2.70	165	89	99.81	0	99.42	No
523_SS_0113	Meric et al., 2018	NA	Germany	2002	Joint synovial fluid (direct isolation)	89	1	A	2.50	96	79	99.67	0.19	98.80	No
524_SS_0114	Meric et al., 2018	NA	Germany	2002	Intraoperative isolation	89	1	A	2.50	89	93	99.67	0.02	98.81	No
526_SS_0116	Meric et al., 2018	NA	Germany	2002	Intraoperative isolation	23	5	A	2.47	147	69	99.81	0.09	99.10	No
527_SS_0117	Harris et al., 2016	NA	Germany	2002	Prosthetic Joint	2	5	A	2.59	155	97	99.81	0	99.58	No
533_SS_0125	Meric et al., 2018	NA	Germany	2002	Joint synovial fluid (direct isolation)	88	1	A	2.40	111	73	99.72	0.05	99.47	No
534_SS_0126	Meric et al., 2018	NA	Germany	2002	Intraoperative isolation	88	1	A	2.47	126	91	99.81	0	99.51	No
537_SS_0129	Harris et al., 2016	NA	Germany	2002	Prosthetic Joint	22	5	A	2.59	178	78	99.81	0	99.46	No
538_SS_0130	Meric et al., 2018	NA	Germany	2002	Intraoperative isolation	2	5	A	2.53	166	94	99.81	0.01	99.45	No
542_SS_0134	Meric et al., 2018	NA	Germany	2003	Intraoperative isolation	5	6	A	2.43	121	83	98.69	0.15	99.57	No
543_SS_0135	Meric et al., 2018	NA	Germany	2002	Joint synovial fluid (direct isolation)	297	1	A	2.50	169	81	99.81	0	99.42	No
544_SS_0136	Meric et al., 2018	NA	Germany	2003	Intraoperative isolation	297	1	A	2.41	125	75	99.81	0	99.50	No
547_SS_0139	Meric et al., 2018	NA	Germany	2002	Joint synovial fluid (direct isolation)	22	5	A	2.45	293	63	99.81	0.09	99.59	No
548_SS_0140	Meric et al., 2018	NA	Germany	2003	Intraoperative isolation	22	5	A	2.45	124	63	99.81	0.09	99.57	No
553_SS_0145	Meric et al., 2018	NA	Germany	2003	Joint synovial fluid (direct isolation)	297	1	A	2.50	155	80	99.81	0.84	99.43	No
554_SS_0146	Meric et al., 2018	NA	Germany	2003	Intraoperative isolation	297	1	A	2.50	156	76	99.81	0.94	99.36	No
557_SS_0149	Meric et al., 2018	NA	Germany	2003	Joint synovial fluid (direct isolation)	2	5	A	2.68	128	110	99.81	0	99.51	No
558_SS_0150	Meric et al., 2018	NA	Germany	2003	Intraoperative isolation	2	5	A	2.76	88	123	99.81	0	99.32	No
559_SS_0151	Meric et al., 2018	NA	Germany	2002	Joint synovial fluid (direct isolation)	2	5	A	2.63	131	90	99.22	0	99.39	No
560_SS_0152	Meric et al., 2018	NA	Germany	2003	Intraoperative isolation	2	5	A	2.67	153	82	99.25	0	99.39	No
561_SS_0153	Meric et al., 2018	NA	Germany	2003	Joint synovial fluid (direct isolation)	59	6	A	2.51	152	78	99.81	0	99.41	No
562_SS_0154	Meric et al., 2018	NA	Germany	2003	Intraoperative isolation	59	6	A	2.53	140	103	99.81	0.14	99.48	No
563_SS_0155	Meric et al., 2018	NA	Germany	2002	Joint synovial fluid (direct isolation)	5	6	A	2.52	119	79	99.81	0.09	99.50	No
564_SS_0156	Meric et al., 2018	NA	Germany	2003	Intraoperative isolation	5	6	A	2.52	117	89	99.81	0.09	99.50	No
565_SS_0157	Meric et al., 2018	NA	Germany	2002	Joint synovial fluid (direct isolation)	59	6	A	2.48	110	109	99.81	0.14	99.53	No
566_SS_0158	Meric et al., 2018	NA	Germany	2003	Intraoperative isolation	59	6	A	2.48	153	84	99.81	0	99.52	No
567_SS_0159	Meric et al., 2018	NA	Germany	2002	Joint synovial fluid (direct isolation)	2	5	A	2.52	130	101	99.81	0	99.37	No
568_SS_0160	Meric et al., 2018	NA	Germany	2003	Intraoperative isolation	2	5	A	2.52	125	92	99.81	0.3	99.36	No
573_SS_0165	Harris et al., 2016	NA	Germany	2002	Prosthetic Joint	726	6	A	2.46	112	79	99.25	0.05	99.38	No

575_SS_0167	Harris et al., 2016	NA	Germany	2003	Prosthetic Joint	168	5	A	2.77	79	111	99.81	0	99.25	No
576_SS_0168	Meric et al., 2018	NA	Germany	2003	Intraoperative isolation	168	5	A	2.66	90	107	99.81	0	99.48	No
579_SS_0171	Meric et al., 2018	NA	Germany	2003	Joint synovial fluid (direct isolation)	10	6	A	2.53	147	73	99.81	0	99.23	No
580_SS_0172	Meric et al., 2018	NA	Germany	2003	Intraoperative isolation	10	6	A	2.53	147	88	99.81	0	99.22	No
581_SS_0175	Meric et al., 2018	NA	Germany	2003	Joint synovial fluid (direct isolation)	208	4	B	2.46	276	70	99.67	0	96.92	Yes
582_SS_0176	Meric et al., 2018	NA	Germany	2003	Intraoperative isolation	208	4	B	2.46	251	74	99.67	0	96.94	No
583_SS_0178	Meric et al., 2018	NA	Germany	2003	Joint synovial fluid (direct isolation)	59	6	A	2.42	111	65	99.81	0	99.52	No
584_SS_0179	Meric et al., 2018	NA	Germany	2003	Intraoperative isolation	59	6	A	2.43	124	85	99.81	0.19	99.53	No
585_SS_0181	Meric et al., 2018	NA	Germany	2003	Joint synovial fluid (direct isolation)	59	6	A	2.37	211	59	99.81	0.28	99.60	No
586_SS_0182	Meric et al., 2018	NA	Germany	2003	Intraoperative isolation	59	6	A	2.37	181	59	99.81	0.01	99.57	No
587_SS_0183	Meric et al., 2018	NA	Germany	2003	Joint synovial fluid (direct isolation)	48	5	A	2.45	203	67	99.67	0.58	99.32	No
588_SS_0184	Meric et al., 2018	NA	Germany	2003	Intraoperative isolation	48	5	A	2.44	203	65	99.67	0.56	99.32	Yes
589_SS_0185	Meric et al., 2018	NA	Germany	2003	Joint synovial fluid (direct isolation)	9	1	A	2.52	105	109	99.81	0.66	99.51	No
590_SS_0186	Meric et al., 2018	NA	Germany	2003	Intraoperative isolation	9	1	A	2.51	104	69	99.81	0.66	99.51	No
591_SS_0227	Meric et al., 2018	NA	Germany	Unknown	Nasal cavity	184	1	A	2.48	142	89	99.81	0.08	99.58	No
592_SS_0228	Meric et al., 2018	NA	Germany	Unknown	Nasal cavity	88	1	A	2.44	127	79	99.81	0	99.55	No
593_SS_0229	Meric et al., 2018	NA	Germany	Unknown	Nasal cavity	57	6	A	2.51	117	83	99.7	0	99.47	No
595_SS_0231	Meric et al., 2018	NA	Germany	Unknown	Nasal cavity	59	6	A	2.47	118	92	99.81	0.06	99.39	No
596_SS_0232	Meric et al., 2018	NA	Germany	Unknown	Nasal cavity	88	1	A	2.49	209	93	99.81	0	99.51	No
597_SS_0233	Meric et al., 2018	NA	Germany	Unknown	Nasal cavity	17	6	A	2.44	181	79	99.81	0	99.39	No
598_SS_0234	Meric et al., 2018	NA	Germany	Unknown	Nasal cavity	73	1	A	2.53	152	115	99.67	0.37	99.45	No
599_SS_0235	Meric et al., 2018	NA	Germany	Unknown	Nasal cavity	35	1	A	2.41	502	64	99.81	0	99.44	No
600_SS_0236	Meric et al., 2018	NA	Germany	Unknown	Nasal cavity	73	1	A	2.44	166	81	99.67	0.28	99.48	No
601_SS_0237	Meric et al., 2018	NA	Germany	Unknown	Nasal cavity	5	6	A	2.55	110	83	99.81	0.09	99.43	No
602_SS_0238	Meric et al., 2018	NA	Germany	Unknown	Nasal cavity	7	6	A	2.42	193	66	99.81	0	99.50	No
615_SS_0187	Meric et al., 2018	NA	Germany	2001	Isolated from hospital patient	22	5	A	2.62	184	104	99.81	0	99.46	No
617_SS_0189	Meric et al., 2018	NA	Germany	2001	Isolated from hospital patient	2	5	A	2.59	167	66	99.77	0	99.39	No
618_SS_0190	Meric et al., 2018	NA	Germany	2001	Isolated from hospital patient	2	5	A	2.60	155	99	99.77	0.06	99.42	No
619_SS_0191	Meric et al., 2018	NA	Germany	2001	Isolated from hospital patient	2	5	A	2.59	121	103	99.77	0.01	99.43	No
620_SS_0192	Meric et al., 2018	NA	Germany	1999	Isolated from hospital patient	2	5	A	2.57	130	94	99.81	0	99.42	No
621_SS_0193	Meric et al., 2018	NA	Germany	1999	Isolated from hospital patient	2	5	A	2.51	110	82	99.81	0	99.50	No
622_SS_0194	Meric et al., 2018	NA	Germany	1999	Isolated from hospital patient	22	5	A	2.62	129	84	99.81	0	99.44	No
623_SS_0195	Meric et al., 2018	NA	Germany	1999	Isolated from hospital patient	22	5	A	2.59	105	78	99.76	0	99.46	No
624_SS_0196	Meric et al., 2018	NA	Germany	1999	Isolated from hospital patient	22	5	A	2.63	138	88	99.81	0	99.38	No
625_SS_0197	Meric et al., 2018	NA	Germany	2001	Isolated from hospital patient	2	5	A	2.66	185	58	99.81	0	99.41	No
626_SS_0198	Meric et al., 2018	NA	Germany	2001	Isolated from hospital patient	2	5	A	2.67	148	89	99.81	0	99.41	No
627_SS_0199	Meric et al., 2018	NA	Germany	1999	Isolated from hospital patient	2	5	A	2.49	136	103	99.81	0.09	99.56	No
628_SS_0200	Meric et al., 2018	NA	Germany	1999	Isolated from hospital patient	2	5	A	2.48	124	71	99.81	0.09	99.51	No
629_SS_0201	Meric et al., 2018	NA	Germany	2000	Isolated from hospital patient	22	5	A	2.56	124	95	99.81	0	99.47	No
630_SS_0202	Meric et al., 2018	NA	Germany	2000	Isolated from hospital patient	22	5	A	2.58	91	88	99.81	0	99.36	No
631_SS_0203	Meric et al., 2018	NA	Germany	2000	Isolated from hospital patient	2	5	A	2.69	110	73	99.81	0	99.31	No
632_SS_0204	Meric et al., 2018	NA	Germany	2000	Isolated from hospital patient	2	5	A	2.51	55	126	99.77	0.11	99.47	No
633_SS_0205	Meric et al., 2018	NA	Germany	2000	Isolated from hospital patient	2	5	A	2.45	123	80	99.77	0.02	99.57	No
634_SS_0206	Meric et al., 2018	NA	Germany	2002	Isolated from hospital patient	726	6	A	2.46	155	87	99.81	0.28	99.39	No
635_SS_0207	Meric et al., 2018	NA	Germany	2002	Isolated from hospital patient	726	6	A	2.44	182	84	99.81	0.28	99.41	No
636_SS_0208	Meric et al., 2018	NA	Germany	1999	Isolated from hospital patient	2	5	A	2.48	95	82	99.81	0.09	99.54	No
637_SS_0209	Meric et al., 2018	NA	Germany	1999	Isolated from hospital patient	2	5	A	2.71	117	104	99.81	0	99.37	No
638_SS_0210	Meric et al., 2018	NA	Germany	2002	Isolated from hospital patient	2	5	A	2.52	74	132	99.81	0.34	99.37	No
639_SS_0211	Meric et al., 2018	NA	Germany	2002	Isolated from hospital patient	2	5	A	2.44	96	88	99.81	0	99.43	No
640_SS_0212	Meric et al., 2018	NA	Germany	2002	Isolated from hospital patient	2	5	A	2.45	137	92	99.53	0	99.44	No
641_SS_0213	Meric et al., 2018	NA	Germany	2001	Isolated from hospital patient	2	5	A	2.51	103	92	99.81	0	99.37	No
642_SS_0214	Meric et al., 2018	NA	Germany	2001	Isolated from hospital patient	2	5	A	2.60	284	102	99.81	0.53	99.40	No
643_SS_0215	Meric et al., 2018	NA	Germany	1998	Isolated from hospital patient	2	5	A	2.59	109	122	99.81	0.02	99.38	No
644_SS_0216	Meric et al., 2018	NA	Germany	1998	Isolated from hospital patient	2	5	A	2.59	101	124	99.81	0	99.39	No
645_SS_0217	Meric et al., 2018	NA	Germany	1998	Isolated from hospital patient	2	5	A	2.59	109	115	99.81	0	99.40	No
646_SS_0218	Meric et al., 2018	NA	Germany	1998	Isolated from hospital patient	2	5	A	2.54	185	69	99.81	0	99.43	No
647_SS_0219	Meric et al., 2018	NA	Germany	1998	Isolated from hospital patient	2	5	A	2.55	120	103	99.81	0	99.34	No
648_SS_0220	Meric et al., 2018	NA	Germany	1998	Isolated from hospital patient	2	5	A	2.55	121	99	99.81	0.28	99.40	No
649_SS_0221	Meric et al., 2018	NA	Germany	2001	Isolated from hospital patient	2	5	A	2.45	137	75	99.81	0.28	99.52	No
651_SS_0223	Meric et al., 2018	NA	Germany	2001	Isolated from hospital patient	5	6	A	2.57	96	141	99.81	0.09	99.49	No
652_SS_0224	Meric et al., 2018	NA	Germany	2001	Isolated from hospital patient	5	6	A	2.55	119	76	99.81	0.19	99.45	No
653_SS_0225	Meric et al., 2018	NA	Germany	2001	Isolated from hospital patient	5	6	A	2.56	106	80	99.81	0.09	99.48	No
654_SS_0226	Meric et al., 2018	NA	Germany	2001	Isolated from hospital patient	5	6	A	2.56	113	82	99.81	0.09	99.46	No
707_SS_0510	Meric et al., 2018	NA	Germany	Unknown	Cerebrospinal fluid shunt	10	6	A	2.45	157	58	99.81	0	99.23	No
708_SS_0511	Meric et al., 2018	NA	Germany	Unknown	Cerebrospinal fluid shunt	10	6	A	2.45	177	60	99.81	0	99.20	No
709_SS_0516	Meric et al., 2018	NA	Germany	Unknown	Port-catheter system	89	1	A	2.41	121	70	99.67	0.06	98.89	No
711_SS_0249	Harris et al., 2016	NA	Germany	Unknown	Animal	55	6	A	2.49	130	94	99.81	0.01	99.37	No
712_SS_0250	Harris et al., 2016	NA	Germany	Unknown	Animal	256	1	A	2.49	99	160	99.81	0.09	99.40	Yes
722_SS_0261	Harris et al., 2016	NA	Germany	Unknown	Animal	19	3	C	2.53	272	48	99.67	0	98.12	No
734_SS_0276	Harris et al., 2016	NA	Germany	Unknown	Animal	93	1	A	2.43	85	85	99.81	0.56	99.37	No
743_SS_0286	Harris et al., 2016	NA	Germany	Unknown	Animal	59	6	A	2.39	111	91	99.81	0	99.53	No
748_SS_0291	Harris et al., 2016	NA	Germany	Unknown	Animal	110	6	A	2.49	109	97	99.81	0	99.44	No
772_SS_0315	Harris et al., 2016	NA	Germany	Unknown	Nasal	5	6	A	2.61	96	91	99.81	0	99.48	No
778_SS_0321	Harris et al., 2016	NA	Germany	Unknown	Nasal	5	6	A	2.57	152	71	99.81	0	99.53	No
779_SS_0322	Meric et al., 2018	NA	Germany	Unknown	Nasal cavity	5	6	A	2.61	94	91	99.81	0	99.51	No
780_SS_0323	Meric et al., 2018	NA	Germany	Unknown	Nasal cavity	66	4	B	2.58	169	95	99.67	0.09	96.83	Yes
781_SS_0324	Meric et al., 2018	NA	Germany	Unknown	Nasal cavity	48	5	A	2.51	173	101	99.67	0.56	99.31	No
782_SS_0325	Meric et al., 2018	NA	Germany	Unknown	Stethoscope in operative room	10	6	A	2.52	110	107	99.81	0.02	99.20	No
798_SS_0512	Meric et al., 2018	NA	Germany	Unknown	Isolated from hospital patient	86	1	A	2.43	224	74	99.81	0	99.37	No
799_SS_0513	Meric et al., 2018	NA	Germany	Unknown	Central venous catheter	86	1	A	2.44	169	81	99.81	0	99.40	No
800_SS_0514	Meric et al., 2018	NA	Germany	Unknown	Hospital patient with bacteremia	114	1	A	2.41	214	73	99.81	0	99.42	No
801_SS_0515	Meric et al., 2018	NA	Germany	Unknown	Hospital patient with bacteremia	2	5	A	2.50	113	130	99.81	0.09	99.42	No
GCF_000417985	NCBI SSB	NA	Germany	Unknown	skin	702	4	B	2.58	98	148	99.67	0.28	96.89	No
GCF_000705035	NCBI SSB	NA	Germany	Unknown	central veneous catheter	114	1	A	2.39	110	45	99.81	0	99.44	No
GCF_000705055	NCBI SSB	NA	Germany	Unknown	NA	1015	5	A	2.50	113	71	99.81	0	99.35	No
GCF_000705075	NCBI SSB	NA	Germany	Unknown	feces	86	1	A	2.42	82	74	99.81	0	99.46	No
GCF_001622455	NCBI SSB	NA	Germany	Unknown	clinic	2	5	A	2.71	82	70	99.81	0	99.39	No

GCF_001622495	NCBI SSB	NA	Germany	Unknown	clinic	2	5	A	2.68	54	86	99.81	0	99.37	No
GCF_001622545	NCBI SSB	NA	Germany	Unknown	clinic	2	5	A	2.71	71	76	99.81	0	99.33	No
GCF_001622555	NCBI SSB	NA	Germany	Unknown	clinic	2	5	A	2.71	87	72	99.81	0.19	99.42	No
GCF_001622605	NCBI SSB	NA	Germany	Unknown	clinic	2	5	A	2.70	50	101	99.25	0.19	99.35	No
GCF_001622625	NCBI SSB	NA	Germany	Unknown	clinic	2	5	A	2.74	83	72	99.81	0.19	99.36	No
GCF_001622635	NCBI SSB	NA	Germany	Unknown	clinic	2	5	A	2.73	58	96	99.81	0.19	99.46	No
GCF_001622665	NCBI SSB	NA	Germany	Unknown	clinic	2	5	A	2.72	76	72	99.81	0.19	99.43	No
GCF_001622685	NCBI SSB	NA	Germany	Unknown	clinic	2	5	A	2.71	69	77	99.81	0.19	99.40	No
GCF_001622695	NCBI SSB	NA	Germany	Unknown	clinic	2	5	A	2.75	62	79	99.81	0.19	99.36	No
GCF_001622715	NCBI SSB	NA	Germany	Unknown	clinic	2	5	A	2.65	70	75	98.13	0.19	99.30	No
GCF_001622755	NCBI SSB	NA	Germany	Unknown	clinic	2	5	A	2.75	97	69	99.81	0.19	99.40	No
GCF_001622805	NCBI SSB	NA	Germany	Unknown	clinic	2	5	A	2.73	96	67	99.81	0.19	99.40	No
GCF_001622825	NCBI SSB	NA	Germany	Unknown	clinic	2	5	A	2.71	95	64	99.81	0	99.32	No
GCF_001622875	NCBI SSB	NA	Germany	Unknown	clinic	2	5	A	2.72	83	68	99.81	0	99.37	No
GCF_001622905	NCBI SSB	NA	Germany	Unknown	clinic	2	5	A	2.72	83	65	99.81	0	99.32	No
GCF_001658445	NCBI SSB	NA	Germany	Unknown	NA	5	6	A	2.55	87	73	99.81	0	99.50	No
GCF_001658455	NCBI SSB	NA	Germany	Unknown	NA	5	6	A	2.46	96	51	99.81	0.09	99.55	No
GCF_001658465	NCBI SSB	NA	Germany	Unknown	NA	59	6	A	2.46	130	49	99.81	0	99.46	No
GCF_001658475	NCBI SSB	NA	Germany	Unknown	NA	5	6	A	2.48	79	96	99.81	0	99.48	No
GCF_001658525	NCBI SSB	NA	Germany	Unknown	NA	5	6	A	2.38	105	56	99.81	0	99.59	No
GCF_001658535	NCBI SSB	NA	Germany	Unknown	bronchoalveolar lavage	5	6	A	2.40	63	72	98.69	0	99.45	No
GCF_001658555	NCBI SSB	NA	Germany	Unknown	NA	5	6	A	2.50	83	75	99.81	0	99.51	No
GCF_001658565	NCBI SSB	NA	Germany	Unknown	NA	59	6	A	2.46	130	50	99.81	0	99.49	No
GCF_001658635	NCBI SSB	NA	Germany	Unknown	blood	2	5	A	2.67	63	77	99.81	0.56	99.39	No
GCF_001658705	NCBI SSB	NA	Germany	Unknown	clinic	2	5	A	2.72	70	79	99.81	0	99.35	No
GCF_001658715	NCBI SSB	NA	Germany	Unknown	clinic	2	5	A	2.60	97	59	99.81	0	99.41	No
GCF_001658725	NCBI SSB	NA	Germany	Unknown	clinic	2	5	A	2.75	66	88	99.81	0.47	99.36	No
GCF_001658785	NCBI SSB	NA	Germany	Unknown	clinic	2	5	A	2.71	59	93	99.81	0	99.39	No
GCF_001658805	NCBI SSB	NA	Germany	Unknown	clinic	2	5	A	2.72	83	72	99.81	0	99.40	No
GCF_001658815	NCBI SSB	NA	Germany	Unknown	clinic	2	5	A	2.72	71	82	99.81	0	99.36	No
GCF_001658865	NCBI SSB	NA	Germany	Unknown	clinic	2	5	A	2.57	70	72	98.13	0	99.34	No
GCF_001658895	NCBI SSB	NA	Germany	Unknown	clinic	2	5	A	2.51	83	73	99.81	0	99.43	No
GCF_001658905	NCBI SSB	NA	Germany	Unknown	clinic	2	5	A	2.50	72	78	99.81	0.06	99.39	Yes
GCF_001658945	NCBI SSB	NA	Germany	Unknown	clinic	2	5	A	2.53	67	92	98.41	0	99.46	No
GCF_001658985	NCBI SSB	NA	Germany	Unknown	clinic	2	5	A	2.53	72	93	99.81	0	99.39	No
GCF_001658995	NCBI SSB	NA	Germany	Unknown	clinic	2	5	A	2.73	61	89	99.81	0.06	99.40	No
GCF_001659025	NCBI SSB	NA	Germany	Unknown	clinic	2	5	A	2.64	83	71	99.81	0	99.40	No
GCF_001659045	NCBI SSB	NA	Germany	Unknown	clinic	1050	5	A	2.64	94	65	99.81	0	99.37	No
GCF_001659065	NCBI SSB	NA	Germany	Unknown	clinic	2	5	A	2.67	82	73	99.81	0	99.42	No
GCF_001659075	NCBI SSB	NA	Germany	Unknown	clinic	2	5	A	2.67	83	79	99.81	0	99.37	No
GCF_001659105	NCBI SSB	NA	Germany	Unknown	clinic	2	5	A	2.74	53	88	99.81	0.19	99.43	No
GCF_001659125	NCBI SSB	NA	Germany	Unknown	clinic	2	5	A	2.76	66	91	99.81	0.19	99.41	No
GCF_001659145	NCBI SSB	NA	Germany	Unknown	clinic	2	5	A	2.67	63	83	99.81	0	99.36	No
GCF_001659165	NCBI SSB	NA	Germany	Unknown	clinic	2	5	A	2.63	82	69	99.81	0	99.39	No
GCF_001659185	NCBI SSB	NA	Germany	Unknown	clinic	2	5	A	2.60	69	89	99.81	0	99.44	No
GCF_001659205	NCBI SSB	NA	Germany	Unknown	clinic	2	5	A	2.63	71	76	99.81	0	99.42	No
GCF_001659225	NCBI SSB	NA	Germany	Unknown	clinic	2	5	A	2.42	53	94	95.25	0	99.39	No
GCF_001659235	NCBI SSB	NA	Germany	Unknown	skin; prosthetic joint infection	2	5	A	2.72	81	80	99.81	0	99.36	No
GCF_001659245	NCBI SSB	NA	Germany	Unknown	skin; prosthetic joint infection	2	5	A	2.46	96	49	99.81	0.56	99.51	No
GCF_001659255	NCBI SSB	NA	Germany	Unknown	skin; prosthetic joint infection	5	6	A	2.50	72	73	99.81	0.09	99.46	No
GCF_001659315	NCBI SSB	NA	Germany	Unknown	clinic	2	5	A	2.48	96	57	99.81	0	99.38	No
GCF_001659345	NCBI SSB	NA	Germany	Unknown	clinic	2	5	A	2.72	85	95	99.14	0.44	99.37	No
GCF_001659355	NCBI SSB	NA	Germany	Unknown	clinic	5	6	A	2.50	89	53	99.81	0	99.47	No
GCF_001659395	NCBI SSB	NA	Germany	Unknown	housing complex	5	6	A	2.54	83	78	99.81	0.28	99.43	No
GCF_001659425	NCBI SSB	NA	Germany	Unknown	clinic	5	6	A	2.44	96	55	99.81	0	99.52	No
GCF_001659475	NCBI SSB	NA	Germany	Unknown	cowhouse	59	6	A	2.40	110	43	99.81	0	99.55	No
GCF_001659505	NCBI SSB	NA	Germany	Unknown	cowhouse	59	6	A	2.40	211	33	99.81	0	99.60	No
GCF_001659515	NCBI SSB	NA	Germany	Unknown	cowhouse	59	6	A	2.39	81	71	99.81	0	99.52	No
GCF_001662155	NCBI SSB	NA	Germany	Unknown	clinic	2	5	A	2.68	54	93	99.53	0	99.38	No
GCF_001662165	NCBI SSB	NA	Germany	Unknown	clinic	2	5	A	2.70	53	99	99.81	0	99.28	No
GCF_001662175	NCBI SSB	NA	Germany	Unknown	clinic	2	5	A	2.71	64	87	99.81	0	99.37	No
GCF_001662225	NCBI SSB	NA	Germany	Unknown	clinic	2	5	A	2.71	64	84	99.81	0	99.30	No
GCF_001662985	NCBI SSB	NA	Germany	Unknown	clinic	2	5	A	2.70	59	83	99.81	0	99.41	No
GCF_001663015	NCBI SSB	NA	Germany	Unknown	clinic	2	5	A	2.78	54	97	99.81	0.19	99.41	No
GCF_001663025	NCBI SSB	NA	Germany	Unknown	clinic	2	5	A	2.68	54	91	99.81	0	99.34	No
GCF_001683645	NCBI SSB	NA	Germany	Unknown	blood	5	6	A	2.44	51	79	99.25	0.01	99.52	No
GCF_002085695	NCBI SSB	NA	Germany	Unknown	central veneous catheter	86	1	A	2.47	2455	2	99.81	0	99.39	No
GCF_011038575	NCBI SSB	NA	Germany	Unknown	central veneous catheter	23	5	A	2.45	2449	1	99.81	0.56	99.18	Yes
GCF_013317125	NCBI SSB	NA	Germany	Unknown	overnight culture	2	5	A	2.52	2518	1	99.81	0	99.49	Yes
GCF_016727385	NCBI SSB	NA	Germany	Unknown	NA	387	6	A	2.48	2440.37	3	99.77	0	99.30	No
GCF_017315545	NCBI SSB	NA	Germany	Unknown	skin	153	6	A	2.51	172.01	45	99.67	0.67	99.45	No
GCF_017315595	NCBI SSB	NA	Germany	Unknown	skin	73	1	C	2.47	577.93	22	99.67	0	98.10	No
GCF_017583025	NCBI SSB	NA	Germany	Unknown	Supermarket	19	3	A	2.45	144.60	63	99.81	0	99.40	Yes
GCF_019329245	NCBI SSB	NA	Germany	Unknown	NA	924	4	A	2.73	2712.68	3	99.72	0	99.32	No
GCF_019329325	NCBI SSB	NA	Germany	Unknown	NA	2	5	A	2.73	2712.67	3	99.81	0.09	99.36	No
GCF_019329365	NCBI SSB	NA	Germany	Unknown	NA	2	5	A	2.73	2713.51	3	99.81	0	99.36	No
GCF_019329405	NCBI SSB	NA	Germany	Unknown	NA	2	5	A	2.73	2713.96	3	99.81	0	99.32	No
GCF_019329425	NCBI SSB	NA	Germany	Unknown	NA	2	5	A	2.78	2752.43	3	99.81	0.09	99.38	No
GCF_019329505	NCBI SSB	NA	Germany	Unknown	NA	2	5	A	2.74	2715.34	3	99.72	0	99.33	No
GCF_019329525	NCBI SSB	NA	Germany	Unknown	NA	2	5	A	2.73	2712.68	2	99.81	0	99.34	No
GCF_019329545	NCBI SSB	NA	Germany	Unknown	NA	2	5	A	2.75	2712.68	3	99.81	0.09	99.35	No
GCF_019329565	NCBI SSB	NA	Germany	Unknown	NA	2	5	A	2.73	2712.68	3	99.81	0.09	99.35	No
GCF_019329585	NCBI SSB	NA	Germany	Unknown	NA	2	5	A	2.73	2719.93	2	99.81	0	99.32	No
GCF_019329605	NCBI SSB	NA	Germany	Unknown	NA	2	5	A	2.73	2712.68	3	99.81	0.09	99.36	No
GCF_019329625	NCBI SSB	NA	Germany	Unknown	NA	2	5	A	2.65	2634.71	2	99.72	0	99.52	No
GCF_019329645	NCBI SSB	NA	Germany	Unknown	NA	2	5	A	2.75	2712.77	3	99.81	0.09	99.34	No
GCF_019329665	NCBI SSB	NA	Germany	Unknown	NA	2	5	A	2.82	2802.21	2	99.81	0.13	99.38	No

GCF_019329685	NCBI SSB	NA	Germany	Unknown	NA	2	5	A	2.73	2712.68	2	99.81	0	99.34	No
GCF_019329705	NCBI SSB	NA	Germany	Unknown	NA	2	5	A	2.73	2712.68	2	99.81	0.09	99.38	No
GCF_019329725	NCBI SSB	NA	Germany	Unknown	NA	2	5	A	2.73	2712.66	2	99.81	0	99.36	No
GCF_019329745	NCBI SSB	NA	Germany	Unknown	NA	2	5	A	2.83	2812.60	3	99.81	0	99.34	No
GCF_019329765	NCBI SSB	NA	Germany	Unknown	NA	2	5	A	2.73	2712.52	3	99.81	0	99.32	No
GCF_019329785	NCBI SSB	NA	Germany	Unknown	NA	2	5	A	2.74	2712.68	4	99.81	0	99.34	No
GCF_019329805	NCBI SSB	NA	Germany	Unknown	NA	2	5	A	2.73	2712.52	2	99.81	0.09	99.35	No
GCF_019330045	NCBI SSB	NA	Germany	Unknown	NA	2	5	A	2.73	2712.65	3	99.81	0.09	99.35	No
GCF_019330105	NCBI SSB	NA	Germany	Unknown	NA	2	5	A	2.74	2713.99	3	99.81	0	99.35	No
GCF_019330125	NCBI SSB	NA	Germany	Unknown	NA	2	5	A	2.73	2712.68	3	99.81	0	99.32	No
GCF_019330145	NCBI SSB	NA	Germany	Unknown	NA	2	5	A	2.75	2712.68	3	99.81	0.09	99.36	No
GCF_019330165	NCBI SSB	NA	Germany	Unknown	NA	2	5	A	2.73	2712.68	3	99.81	0	99.35	No
GCF_019330185	NCBI SSB	NA	Germany	Unknown	NA	2	5	A	2.75	2713.95	3	99.72	0	99.35	No
GCF_019330205	NCBI SSB	NA	Germany	Unknown	NA	2	5	A	2.73	2712.68	2	99.81	0	99.33	No
GCF_019330225	NCBI SSB	NA	Germany	Unknown	NA	2	5	A	2.73	2712.68	3	99.81	0.09	99.36	No
GCF_019330245	NCBI SSB	NA	Germany	Unknown	NA	2	5	A	2.75	2712.68	3	99.81	0.09	99.37	No
GCF_019787665	NCBI SSB	NA	Germany	Unknown	NA	2	5	A	2.75	2712.68	3	99.81	0.09	99.37	No
1582_HESN074B	PubMLST	NA	Ghana	2018	sepsis	993	4	B	2.44	148	30	99.81	0	96.90	No
1584_HESN038B	PubMLST	NA	Ghana	2019	sepsis	994	4	B	2.68	78	115	99.67	0.9	96.76	No
GCF_015070395	NCBI SSB	NA	Ghana	Unknown	blood	16	1	B	2.43	233.41	25	99.67	0	96.97	Yes
GCF_015070555	NCBI SSB	NA	Ghana	Unknown	nasal mucosa	226	4	A	2.37	79.02	55	99.67	0.02	99.34	No
GCF_015070585	NCBI SSB	NA	Ghana	Unknown	blood	48	5	B	2.68	78.19	115	99.67	0.9	96.76	No
GCF_015070605	NCBI SSB	NA	Ghana	Unknown	blood	994	4	B	2.44	148.42	30	99.81	0	96.90	No
GCF_015070675	NCBI SSB	NA	Ghana	Unknown	blood	993	4	C	2.41	155.34	22	99.67	0	98.37	No
GCF_015070735	NCBI SSB	NA	Ghana	Unknown	blood	490	3	C	2.42	129.62	41	99.67	0.02	98.37	No
GCF_022815765	NCBI SSB	NA	Ghana	Unknown	nasal swab	8	6	B	2.55	269.69	39	99.67	0	96.90	No
GRE34	Meric et al., 2015	NA	Greece	1998	n.a.	69	6	A	2.56	132	76	99.81	0	99.42	No
GRE41	Meric et al., 2015	NA	Greece	1998	n.a.	83	6	A	2.47	132	78	99.81	0	99.27	No
GRE53	Meric et al., 2015	NA	Greece	1998	n.a.	1023	4	B	2.54	157	62	99.67	0.12	96.86	No
HUR105	Meric et al., 2015	NA	Hungary	1998	Sputum	23	5	A	2.49	130	97	99.81	0	99.03	No
HUR51	Meric et al., 2015	NA	Hungary	1997	Wound	1025	4	B	2.44	295	39	99.25	0	97.07	Yes
ICE102	Meric et al., 2015	NA	Iceland	1998	Wound	21	6	A	2.68	94	105	99.81	0.62	99.45	No
ICE122	Meric et al., 2015	NA	Iceland	1998	Wound	215	1	A	2.62	106	94	99.81	0.28	99.36	No
ICE18	Meric et al., 2015	NA	Iceland	1997	Wound	1	1	A	2.49	130	70	99.81	0.47	99.38	No
ICE192	Meric et al., 2015	NA	Iceland	1998	Blood	5	6	A	2.46	110	62	99.81	0	99.50	No
ICE21	Meric et al., 2015	NA	Iceland	1997	Wound	1026	1	A	2.61	141	83	99.81	0.28	99.34	No
ICE24	Meric et al., 2015	NA	Iceland	1997	Wound	38	1	A	2.48	104	87	99.25	0	99.37	Yes
ICE26	Meric et al., 2015	NA	Iceland	1997	Blood	248	1	A	2.69	118	89	99.81	0	99.59	No
ICE7	Meric et al., 2015	NA	Iceland	1997	Blood	2	5	A	2.72	92	97	99.81	0.04	99.27	No
ICE87	Meric et al., 2015	NA	Iceland	1997	Wound	40	1	A	2.61	110	87	99.81	0	99.13	No
ICE9	Meric et al., 2015	NA	Iceland	1997	Urine	6	1	A	2.49	105	102	99.25	0.38	99.53	No
ICE95	Meric et al., 2015	NA	Iceland	1997	Wound	10	6	A	2.44	132	75	99.81	0.56	99.12	No
ICE99	Meric et al., 2015	NA	Iceland	1997	Wound	20	1	A	2.44	154	58	99.81	0	99.14	No
GCF_001465365	NCBI SSB	NA	India	Unknown	Rice seed	836	4	B	2.48	66	85	99.67	0	96.77	No
GCF_001465375	NCBI SSB	NA	India	Unknown	Rice seed	836	4	B	2.50	175	38	99.67	0	96.75	No
GCF_001465385	NCBI SSB	NA	India	Unknown	Rice seed	836	4	B	2.49	96	77	99.67	0	96.82	No
GCF_001476515	NCBI SSB	NA	India	Unknown	NA	1017	4	B	2.54	228	66	99.67	0.38	96.83	No
GCF_001476525	NCBI SSB	NA	India	Unknown	NA	1017	4	B	2.56	109	96	99.67	0.74	96.82	No
GCF_002892285	NCBI SSB	NA	India	Unknown	groundwater	81	6	A	2.47	56	86	96.49	0.04	99.38	No
GCF_003956755	NCBI SSB	NA	India	Unknown	skin	691	4	B	2.47	75	72	99.11	0.66	96.84	No
GCF_003956765	NCBI SSB	NA	India	Unknown	skin	691	4	B	2.53	58	83	99.67	2.44	96.82	No
GCF_003956785	NCBI SSB	NA	India	Unknown	skin	691	4	B	2.48	90	60	99.67	0.66	96.87	No
GCF_003956805	NCBI SSB	NA	India	Unknown	skin	691	4	B	2.48	100	52	99.67	0.66	96.81	No
GCF_003956855	NCBI SSB	NA	India	Unknown	Female Ear	691	4	B	2.47	75	78	98.74	0.66	96.84	No
GCF_003956885	NCBI SSB	NA	India	Unknown	Female Behind the ears	691	4	B	2.47	69	75	99.67	0.66	96.81	No
GCF_003956905	NCBI SSB	NA	India	Unknown	Female Behind the ears	Incomplete	#N/A	B	2.46	62	100	98.83	0.66	96.84	No
GCF_003956955	NCBI SSB	NA	India	Unknown	Male Behind the ears	691	4	B	2.44	52	133	98.01	0.85	96.74	No
GCF_003956965	NCBI SSB	NA	India	Unknown	Female Behind the ears	691	4	B	2.47	102	74	99.61	0.66	96.79	No
GCF_003956995	NCBI SSB	NA	India	Unknown	Female Behind the ears	691	4	B	2.48	59	77	99.67	0.66	96.83	No
GCF_003957035	NCBI SSB	NA	India	Unknown	Female Nares	691	4	B	2.48	75	65	99.67	1.23	96.86	No
GCF_003979615	NCBI SSB	NA	India	Unknown	Female Nares	171	3	C	2.54	63	78	99.11	0.01	98.23	Yes
GCF_003979655	NCBI SSB	NA	India	Unknown	Female Nares	59	6	A	2.42	61	83	99.81	0	99.54	No
GCF_003979695	NCBI SSB	NA	India	Unknown	Female Behind the ears	59	6	A	2.49	59	94	99.81	0	99.48	No
GCF_003980055	NCBI SSB	NA	India	Unknown	skin	691	4	B	2.48	55	107	99.67	1	96.87	No
GCF_003980155	NCBI SSB	NA	India	Unknown	skin	384	6	A	2.50	55	91	99.81	0.47	99.41	No
GCF_003980205	NCBI SSB	NA	India	Unknown	skin	691	4	B	2.48	70	70	99.11	0.78	96.91	Yes
GCF_003980285	NCBI SSB	NA	India	Unknown	NA	691	4	B	2.58	123	107	99.67	1.91	96.89	No
GCF_004343675	NCBI SSB	NA	India	Unknown	ear	35	1	A	2.53	96	100	96.82	0.11	99.24	No
GCF_019149045	NCBI SSB	NA	India	Unknown	Milk	769	1	A	2.41	59.44	76	99.81	0.28	99.50	No
GCF_019149245	NCBI SSB	NA	India	Unknown	Milk	1157	6	A	2.46	78.70	67	99.67	0.91	99.32	No
GCF_019165085	NCBI SSB	NA	India	Unknown	milk	1158	6	B	2.45	75.49	83	99.39	0.02	96.89	No
GCF_021188745	NCBI SSB	NA	India	Unknown	milk	2	5	B	2.46	481.85	55	99.67	0	96.92	Yes
GCF_021190395	NCBI SSB	NA	India	Unknown	Rectal swab	1159	4	B	2.43	434.28	35	99.67	0	96.79	Yes
GCF_021190495	NCBI SSB	NA	India	Unknown	nasal swab	1160	4	B	2.45	178.16	60	99.67	0.02	96.81	No
GCF_021190595	NCBI SSB	NA	India	Unknown	Cow Milk	1161	4	B	2.53	198.47	61	99.67	0.09	96.88	No
GCF_021190695	NCBI SSB	NA	India	Unknown	Human community	1162	4	A	2.43	110.40	75	99.81	0	99.49	No
GCF_021190975	NCBI SSB	NA	India	Unknown	Cow milk	57	6	B	2.55	537.45	67	99.67	0	96.86	Yes
GCF_021191135	NCBI SSB	NA	India	Unknown	NA	1163	4	B	2.55	293.54	40	99.67	0.44	96.81	Yes
GCF_021191175	NCBI SSB	NA	India	Unknown	Cow Milk	1164	4	B	2.46	220.94	50	99.67	0	96.86	Yes
GCF_021191255	NCBI SSB	NA	India	Unknown	Cow milk	987	4	B	2.55	275.69	53	99.67	0.09	96.84	No
GCF_021191295	NCBI SSB	NA	India	Unknown	Human community	1165	4	A	2.44	151.92	69	99.81	0.28	99.35	No
GCF_021191715	NCBI SSB	NA	India	Unknown	Human community	575	6	A	2.43	126.30	74	99.81	0	99.46	No
GCF_021215415	NCBI SSB	NA	India	Unknown	NA	57	6	A	2.44	214.90	46	99.81	0	99.43	No
GCF_003325735	NCBI SSB	NA	Ireland	Unknown	cerebrospinal fluid	297	1	A	2.54	2481	4	99.81	0.56	99.45	No
GCF_009685135	NCBI SSB	NA	Ireland	Unknown	blood	2	5	A	2.75	2737	2	99.81	0	99.33	No
GCF_009896645	NCBI SSB	NA	Ireland	Unknown	milk	5	6	A	2.51	96	49	99.81	0	99.46	No
GCF_009896875	NCBI SSB	NA	Ireland	Unknown	milk	1056	4	B	2.49	143	49	99.67	0	96.81	No

GCF_009896905	NCBI SSB	NA	Ireland	Unknown	milk	1057	1	A	2.41	331	25	99.81	0	99.41	No
GCF_009896915	NCBI SSB	NA	Ireland	Unknown	milk	225	6	A	2.56	82	59	99.81	0	99.32	No
GCF_009896965	NCBI SSB	NA	Ireland	Unknown	milk	6	1	A	2.59	128	33	99.81	0.09	99.41	No
GCF_009896975	NCBI SSB	NA	Ireland	Unknown	milk	6	1	A	2.59	119	43	99.81	0.09	99.42	No
GCF_009897025	NCBI SSB	NA	Ireland	Unknown	milk	6	1	A	2.59	121	35	99.81	0.09	99.41	No
GCF_009897035	NCBI SSB	NA	Ireland	Unknown	milk	307	6	A	2.56	121	38	99.81	0.56	99.44	No
GCF_009897065	NCBI SSB	NA	Ireland	Unknown	milk	35	1	A	2.42	121	37	99.81	0.56	99.40	No
GCF_009897075	NCBI SSB	NA	Ireland	Unknown	milk	307	6	A	2.56	170	36	99.81	0.56	99.44	No
GCF_009897085	NCBI SSB	NA	Ireland	Unknown	milk	54	1	A	2.48	205	31	99.81	0.02	99.31	No
GCF_009897125	NCBI SSB	NA	Ireland	Unknown	milk	307	6	A	2.56	174	32	99.81	0.56	99.38	No
GCF_009897145	NCBI SSB	NA	Ireland	Unknown	milk	5	6	A	2.49	96	45	99.81	0	99.50	No
GCF_009897165	NCBI SSB	NA	Ireland	Unknown	milk	5	6	A	2.49	96	44	99.81	0	99.48	No
GCF_009897185	NCBI SSB	NA	Ireland	Unknown	milk	5	6	A	2.49	96	46	99.81	0	99.48	No
GCF_009897195	NCBI SSB	NA	Ireland	Unknown	milk	5	6	A	2.49	104	43	99.81	0	99.45	No
GCF_009897225	NCBI SSB	NA	Ireland	Unknown	milk	5	6	A	2.49	96	44	99.81	0	99.47	No
GCF_009897235	NCBI SSB	NA	Ireland	Unknown	milk	749	1	A	2.53	129	33	99.81	0.62	99.38	No
GCF_009897265	NCBI SSB	NA	Ireland	Unknown	milk	153	6	A	2.42	90	59	99.81	0	99.41	No
GCF_009897305	NCBI SSB	NA	Ireland	Unknown	milk	153	6	A	2.43	120	44	99.81	0	99.39	No
GCF_009897315	NCBI SSB	NA	Ireland	Unknown	milk	88	1	A	2.42	165	35	99.81	0.09	99.54	No
GCF_009897335	NCBI SSB	NA	Ireland	Unknown	milk	57	6	A	2.42	104	44	99.81	0	99.54	No
GCF_009897365	NCBI SSB	NA	Ireland	Unknown	milk	57	6	A	2.42	104	47	99.81	0	99.57	No
GCF_009897375	NCBI SSB	NA	Ireland	Unknown	milk	32	6	A	2.47	120	40	99.67	0	99.15	No
GCF_009897425	NCBI SSB	NA	Ireland	Unknown	milk	57	6	A	2.41	111	45	99.81	0	99.49	No
GCF_009897435	NCBI SSB	NA	Ireland	Unknown	milk	32	6	A	2.48	123	48	99.67	0	99.17	No
GCF_009897505	NCBI SSB	NA	Ireland	Unknown	milk	1058	6	A	2.43	119	46	99.81	0.1	99.50	No
GCF_009897515	NCBI SSB	NA	Ireland	Unknown	milk	73	1	A	2.47	132	36	99.67	0.28	99.45	No
GCF_009897605	NCBI SSB	NA	Ireland	Unknown	milk	35	1	A	2.48	164	40	99.81	0	99.33	No
GCF_009897625	NCBI SSB	NA	Ireland	Unknown	milk	81	6	A	2.50	90	58	99.81	0	99.42	No
GCF_009897655	NCBI SSB	NA	Ireland	Unknown	milk	173	6	A	2.47	119	42	99.81	0	99.28	No
GCF_009897685	NCBI SSB	NA	Ireland	Unknown	milk	130	6	A	2.43	166	35	99.81	0	99.38	No
GCF_009897805	NCBI SSB	NA	Ireland	Unknown	milk	153	6	A	2.50	170	41	99.81	0	99.44	No
GCF_009897825	NCBI SSB	NA	Ireland	Unknown	milk	153	6	A	2.46	139	37	99.81	0	99.44	No
GCF_009897905	NCBI SSB	NA	Ireland	Unknown	milk	190	6	A	2.45	75	64	99.81	0	99.81	No
GCF_009897925	NCBI SSB	NA	Ireland	Unknown	milk	769	1	A	2.48	98	42	99.7	0.06	99.38	No
GCF_009897935	NCBI SSB	NA	Ireland	Unknown	milk	769	1	A	2.48	119	42	99.7	0.06	99.36	No
GCF_009897945	NCBI SSB	NA	Ireland	Unknown	milk	190	6	A	2.38	96	44	99.81	0	99.79	No
GCF_009897975	NCBI SSB	NA	Ireland	Unknown	milk	769	1	A	2.42	146	35	99.7	0.06	99.39	No
GCF_009898025	NCBI SSB	NA	Ireland	Unknown	milk	1059	6	A	2.44	199	38	99.81	0	99.39	No
GCF_009898055	NCBI SSB	NA	Ireland	Unknown	milk	130	6	A	2.49	132	46	99.81	0	99.40	No
GCF_009898075	NCBI SSB	NA	Ireland	Unknown	milk	769	1	A	2.42	121	35	99.7	0.06	99.36	No
GCF_009898115	NCBI SSB	NA	Ireland	Unknown	milk	5	6	A	2.51	96	45	99.81	0	99.46	No
GCF_009898145	NCBI SSB	NA	Ireland	Unknown	milk	769	1	A	2.42	146	34	99.7	0.06	99.38	No
GCF_000410915	NCBI SSB	NA	Italy	Unknown	cured meat	595	4	B	2.49	84	119	99.67	0.06	96.91	Yes
ITL299	Meric et al., 2015	NA	Italy	1997	Wound	32	6	A	2.46	108	58	99.67	0.28	99.26	No
ITL34	Meric et al., 2015	NA	Italy	1997	Wound	66	4	B	2.61	140	93	99.67	0	96.87	No
GCA_017849975	NCBI SSB	NA	Japan	Unknown	Human skin swab	1175	4	B	2.54	87.85	75	99.63	0	96.99	Yes
GCF_020181395	NCBI SSB	NA	Japan	Unknown	human skin	2	5	A	2.52	2454.13	4	99.81	0	99.52	Yes
JAP263	Meric et al., 2015	NA	Japan	1998	Wound	1025	4	B	2.49	234	61	99.67	0	96.86	No
JAP271	Meric et al., 2015	NA	Japan	1998	Urine	781	6	A	2.43	83	96	97.57	0	99.39	No
GCF_003038655	NCBI SSB	NA	Lebanon	Unknown	sputum	23	5	A	2.46	121	73	99.81	0.66	99.16	No
GCF_000763625	NCBI SSB	NA	Malaysia	Unknown	sputum	2	5	A	2.54	77	63	99.81	0	99.47	No
GCF_002307395	NCBI SSB	NA	Mexico	Unknown	pharyngeal exudade	1052	4	B	2.56	67	146	99.55	0	96.83	No
GCF_004374765	NCBI SSB	NA	Mexico	Unknown	cerebrospinal fluid from newborn	1054	1	A	2.44	77	77	99.81	0	99.39	No
GCF_004374915	NCBI SSB	NA	Mexico	Unknown	Blood culture from newborn	2	5	A	2.55	62	127	99.81	0	99.35	No
GCF_004374925	NCBI SSB	NA	Mexico	Unknown	Blood from newborn	5	6	A	2.55	71	92	99.81	0.09	99.51	No
GCF_004374965	NCBI SSB	NA	Mexico	Unknown	Blood from adult	5	6	A	2.55	78	97	99.81	0.11	99.45	No
GCF_004374995	NCBI SSB	NA	Mexico	Unknown	Blood from newborn	59	6	A	2.45	73	90	99.81	0	99.51	No
GCF_004375005	NCBI SSB	NA	Mexico	Unknown	soft tissue aspirate from adult	1055	4	B	2.52	56	115	99.67	0.19	96.85	No
MEX35	Meric et al., 2015	NA	Mexico	1996	Catheter	559	4	B	2.56	185	73	99.67	0	96.79	No
MEX37	Meric et al., 2015	NA	Mexico	1996	CSF	1027	4	B	2.59	258	53	99.67	0	97.30	Yes
MEX60	Meric et al., 2015	NA	Mexico	1996	Catheter	61	6	A	2.62	150	65	99.81	0	99.42	No
MEX61	Meric et al., 2015	NA	Mexico	1996	Blood	61	6	A	2.61	103	91	99.81	0	99.46	No
GCF_000007645	NCBI SSB	ATCC 12228	NA	Unknown	NA	8	6	A	2.56	2499	7	99.81	0	99.58	No
GCF_000011925	NCBI SSB	RP62A	NA	Unknown	NA	10	6	A	2.64	2617	2	99.81	0	99.21	Yes
GCF_000177115	NCBI SSB	RP62A	NA	Unknown	NA	57	6	A	2.52	277	33	99.62	0.33	99.46	No
GCF_000205405	NCBI SSB	NA	NA	Unknown	NA	1	1	A	2.45	94	61	99.81	0	99.37	No
GCF_000205425	NCBI SSB	NA	NA	Unknown	NA	5	6	A	2.48	77	62	99.81	0.09	99.58	No
GCF_000221705	NCBI SSB	NA	NA	Unknown	NA	5	6	A	2.45	116	44	99.81	0	99.54	No
GCF_000245655	NCBI SSB	NA	NA	Unknown	NA	8	6	A	2.48	51	108	99.81	0	99.55	No
GCF_000245675	NCBI SSB	NA	NA	Unknown	NA	1013	1	A	2.55	83	87	99.81	0.67	99.54	No
GCF_000245695	NCBI SSB	NA	NA	Unknown	NA	1	1	A	2.38	66	78	99.78	0	99.47	No
GCF_000247025	NCBI SSB	NA	NA	Unknown	NA	23	5	A	2.45	66	90	99.81	0.18	99.14	No
GCF_000247045	NCBI SSB	NA	NA	Unknown	NA	1045	4	B	2.55	70	75	99.67	0.24	96.75	No
GCF_000247065	NCBI SSB	NA	NA	Unknown	NA	22	5	A	2.55	110	61	99.81	0.3	99.48	No
GCF_000247105	NCBI SSB	NA	NA	Unknown	NA	73	1	A	2.44	65	80	99.5	0.32	99.52	No
GCF_000247125	NCBI SSB	NA	NA	Unknown	NA	384	6	A	2.44	70	70	99.81	0.63	99.43	No
GCF_000247145	NCBI SSB	NA	NA	Unknown	NA	23	5	A	2.45	69	80	99.7	0	99.09	No
GCF_000247165	NCBI SSB	NA	NA	Unknown	NA	136	6	A	2.43	80	81	99.81	0	99.81	No
GCF_000247205	NCBI SSB	NA	NA	Unknown	NA	329	4	B	2.44	152	36	99.67	0	96.87	No
GCF_000260295	NCBI SSB	NA	NA	Unknown	skin	19	3	C	2.40	83	46	99.67	0	98.27	No
GCF_000443975	NCBI SSB	NA	NA	Unknown	faecal	1014	6	A	2.41	87	56	99.81	0	99.60	No
GCF_000627055	NCBI SSB	NA	NA	Unknown	faecal	1046	1	A	2.40	118	43	99.81	0	99.35	No
GCF_000697805	NCBI SSB	NA	NA	Unknown	faecal	327	1	A	2.49	72	73	99.81	0	99.60	No
GCF_000697905	NCBI SSB	NA	NA	Unknown	NA	4	6	A	2.43	67	68	99.72	0	99.44	No
GCF_000703025	NCBI SSB	NA	NA	Unknown	NA	73	1	A	2.57	2446	9	99.67	0.78	99.43	No
GCF_000751035	NCBI SSB	NA	NA	Unknown	CVP line blood	184	1	A	2.60	2490	5	99.7	0	99.48	No
GCF_000759555	NCBI SSB	NA	NA	Unknown	NA	1016	6	A	2.54	2501	2	99.81	0	99.22	Yes

GCF_003812425	NCBI SSB	NA	NA	Unknown	NA	924	4	B	2.58	2544	3	99.67	0	96.79	No
GCF_006094375	NCBI SSB	NA	NA	Unknown	human eye	5	6	A	2.49	2467	3	99.81	0	99.99	No
GCF_006742205	NCBI SSB	NA	NA	Unknown	NA	5	6	A	2.43	2423	2	99.81	0	100.00	No
GCF_009873455	NCBI SSB	ATCC 12228	NA	Unknown	NA	8	6	A	2.53	2504	2	99.81	0	99.55	No
GCF_014489335	NCBI SSB	NA	NA	Unknown	NA	35	1	A	2.54	2510	2	99.81	0	99.28	No
GCF_014490515	NCBI SSB	NA	NA	Unknown	NA	924	4	B	2.52	2522	1	99.67	0.02	96.91	Yes
GCF_016026915	NCBI SSB	NA	NA	Unknown	NA	54	1	A	2.63	2504.45	8	99.81	0	99.52	No
GCF_022869565	NCBI SSB	NA	NA	Unknown	NA	844	6	A	2.59	2504.46	4	99.81	0	99.53	No
GCF_900086615	NCBI SSB	NA	NA	Unknown	NA	2	5	A	2.85	2793	4	99.81	0	99.21	No
GCF_900108795	NCBI SSB	NA	NA	Unknown	NA	626	6	A	2.43	130	55	99.77	0	99.33	No
GCF_900458515	NCBI SSB	NA	NA	Unknown	clinical	8	6	A	2.64	2518	8	99.79	0	99.53	No
GCF_900458535	NCBI SSB	NA	NA	Unknown	clinical	Incomplete	#N/A	A	2.38	2279	5	98.13	0	99.35	No
GCF_900458575	NCBI SSB	NA	NA	Unknown	clinical	488	1	A	2.61	2495	4	99.81	0	99.38	Yes
GCF_900458585	NCBI SSB	NA	NA	Unknown	clinical	656	6	A	2.59	2476	3	99.67	2.21	99.37	No
GCF_900458605	NCBI SSB	NA	NA	Unknown	NA	5	6	A	2.54	2477	5	99.7	0.67	100.00	No
GCF_900458645	NCBI SSB	NA	NA	Unknown	NA	1060	4	B	2.61	2517	5	99.67	1.15	96.97	Yes
GCF_900458655	NCBI SSB	NA	NA	Unknown	NA	1061	6	A	2.51	2460	3	99.81	0	99.56	No
GCF_900638695	NCBI SSB	NA	NA	Unknown	uncut heroin sample	2	5	A	2.75	2751	1	99.81	0	99.35	Yes
GCF_902159425	NCBI SSB	NA	NA	Unknown	human eye	1062	6	A	2.44	170	40	99.81	0.02	99.47	No
GCF_902509485	NCBI SSB	NA	NA	Unknown	NA	89	1	A	2.55	2480	3	99.67	0	98.71	No
GCF_902509495	NCBI SSB	NA	NA	Unknown	NA	368	4	B	2.54	2526	2	99.67	0	96.86	Yes
GCF_902509505	NCBI SSB	NA	NA	Unknown	NA	2	5	A	2.58	2534	3	99.25	0.28	99.38	No
GCF_902509515	NCBI SSB	NA	NA	Unknown	NA	5	6	A	2.60	2557	3	99.81	0	99.49	No
GCF_902509525	NCBI SSB	NA	NA	Unknown	NA	10	6	A	2.65	2619	2	99.81	0	99.16	No
GCF_902509535	NCBI SSB	NA	NA	Unknown	NA	59	6	A	2.56	2529	3	99.81	0	99.50	No
GCF_905397425	NCBI SSB	NA	NA	Unknown	NA	5	6	A	2.40	53.56	86	99.67	0.28	99.45	No
GCF_910589805	NCBI SSB	NA	NA	Unknown	NA	1173	1	A	2.51	79.55	86	99.81	0.09	99.32	No
GCF_910589875	NCBI SSB	NA	NA	Unknown	NA	2	5	A	2.51	74.87	88	99.81	0	99.36	No
GCF_910589905	NCBI SSB	NA	NA	Unknown	NA	87	5	A	2.55	76.45	97	99.81	0.09	99.39	No
GCF_910589925	NCBI SSB	NA	NA	Unknown	NA	2	5	A	2.51	82.46	93	99.81	0.09	99.38	No
GCF_910589955	NCBI SSB	NA	NA	Unknown	NA	2	5	A	2.51	77.60	86	99.81	0.66	99.50	No
GCF_910590035	NCBI SSB	NA	NA	Unknown	NA	5	6	A	2.45	72.40	79	99.81	0.11	99.36	No
GCF_910590075	NCBI SSB	NA	NA	Unknown	NA	35	1	A	2.48	62.47	112	99.34	0.09	99.38	No
GCF_910590085	NCBI SSB	NA	NA	Unknown	NA	2	5	A	2.45	79.82	78	99.81	0.11	99.40	No
GCF_910590115	NCBI SSB	NA	NA	Unknown	NA	35	1	A	2.51	82.45	89	99.81	0	99.39	No
GCF_910590125	NCBI SSB	NA	NA	Unknown	NA	87	5	A	2.51	76.10	90	99.81	0.66	99.54	No
GCF_910590245	NCBI SSB	NA	NA	Unknown	NA	5	6	A	2.52	79.55	85	99.81	0.09	99.28	No
GCF_910590265	NCBI SSB	NA	NA	Unknown	NA	2	5	A	2.48	60.03	93	99.81	0	99.38	No
GCF_910590275	NCBI SSB	NA	NA	Unknown	NA	87	5	A	2.51	82.46	78	99.81	0.09	99.31	No
GCF_910590295	NCBI SSB	NA	NA	Unknown	NA	2	5	A	2.51	62.47	102	99.81	0.09	99.38	No
GCF_910590335	NCBI SSB	NA	NA	Unknown	NA	2	5	A	2.51	87.29	84	99.81	0	99.40	No
GCF_910590345	NCBI SSB	NA	NA	Unknown	NA	731	5	A	2.52	91.59	82	99.81	0.09	99.43	No
GCF_910590355	NCBI SSB	NA	NA	Unknown	NA	2	5	A	2.50	70.51	84	99.81	0	99.44	No
GCF_910590365	NCBI SSB	NA	NA	Unknown	NA	2	5	A	2.49	90.24	82	99.81	0	99.40	No
GCF_910590375	NCBI SSB	NA	NA	Unknown	NA	87	5	A	2.52	87.29	88	99.81	0	99.45	No
GCF_910591395	NCBI SSB	NA	NA	Unknown	NA	731	5	A	2.48	50.23	109	99.81	0	99.44	No
GCF_910591415	NCBI SSB	NA	NA	Unknown	NA	2	5	A	2.52	65.07	100	99.81	0.09	99.32	No
GCF_012029995	NCBI SSB	NA	Netherlands	Unknown	Healthy skin	329	4	B	2.46	167	44	99.67	0.03	96.80	No
GCF_016107415	NCBI SSB	NA	Netherlands	Unknown	skin	8	6	B	2.46	167.17	44	99.67	0.03	96.86	No
GCF_016107425	NCBI SSB	NA	Netherlands	Unknown	skin	329	4	B	2.46	135.96	49	99.67	0.03	96.76	No
GCF_001651265	NCBI SSB	NA	Norway	Unknown	Poultry processing equipment	1049	3	C	2.48	126	76	99.67	1.12	98.29	Yes
GCF_021367675	NCBI SSB	NA	Norway	Unknown	milk samples	986	6	A	2.48	193.23	44	99.81	0.02	99.40	No
GCF_021367815	NCBI SSB	NA	Norway	Unknown	milk samples	1167	1	A	2.44	166.54	59	99.81	0	99.49	Yes
1076NL	Meric et al., 2015	NA	Portugal	1998	Nasal swab	307	6	A	2.55	126	67	99.81	0.37	99.42	No
1243N	Meric et al., 2015	NA	Portugal	1998	Nasal swab	1031	6	A	2.53	96	91	99.81	0	99.26	Yes
1290N	Meric et al., 2015	NA	Portugal	1999	Nasal swab	1032	3	C	2.53	136	62	99.67	0	98.11	Yes
1340N	Meric et al., 2015	NA	Portugal	1999	Nasal swab	1033	4	B	2.55	262	92	99.11	0	96.79	No
1347N	Meric et al., 2015	NA	Portugal	1999	Nasal swab	2	5	A	2.45	142	69	99.59	0.02	99.50	No
1437N	Meric et al., 2015	NA	Portugal	1999	Nasal swab	1007	4	B	2.50	455	56	99.67	0	96.83	Yes
1474N	Meric et al., 2015	NA	Portugal	1999	Nasal swab	1008	4	B	2.56	159	92	99.67	0	96.83	No
148N	Meric et al., 2015	NA	Portugal	1996	Nasal swab	1009	4	B	2.57	105	103	99.67	0	97.33	Yes
19N	Meric et al., 2015	NA	Portugal	1996	Nasal swab	788	4	B	2.61	186	74	99.67	0.7	96.81	Yes
56N	Meric et al., 2015	NA	Portugal	1996	Nasal swab	1033	4	B	2.56	202	62	99.11	0.3	96.89	Yes
59N	Meric et al., 2015	NA	Portugal	1996	Nasal swab	212	6	A	2.50	144	64	99.75	0	99.43	No
603N	Meric et al., 2015	NA	Portugal	1997	Nasal swab	1033	4	B	2.53	221	96	99.11	0	96.84	No
612N1	Meric et al., 2015	NA	Portugal	1997	Nasal swab	556	6	A	2.46	139	66	99.81	0	99.15	Yes
619N1	Meric et al., 2015	NA	Portugal	1997	Nasal swab	184	1	A	2.47	204	51	99.81	0	99.55	No
619N2	Meric et al., 2015	NA	Portugal	1997	Nasal swab	184	1	A	2.47	132	67	99.81	0	99.57	No
675N1	Meric et al., 2015	NA	Portugal	1997	Nasal swab	1040	4	B	2.53	149	90	99.67	0	96.81	Yes
676NR1	Meric et al., 2015	NA	Portugal	1997	Nasal swab	1010	4	B	2.44	189	62	99.67	0	96.80	No
747N1	Meric et al., 2015	NA	Portugal	1997	Nasal swab	171	3	C	2.51	462	43	99.67	0	98.14	No
790N	Meric et al., 2015	NA	Portugal	1997	Nasal swab	719	4	B	2.56	126	123	99.67	0	97.17	No
803NLR2	Meric et al., 2015	NA	Portugal	1997	Nasal swab	595	4	B	2.53	125	93	99.67	0	96.91	No
93N	Meric et al., 2015	NA	Portugal	1996	Nasal swab	329	4	B	2.52	263	80	99.67	0.64	96.86	No
94N2B	Meric et al., 2015	NA	Portugal	1996	Nasal swab	328	4	B	2.60	171	105	99.67	0	96.78	No
966N	Meric et al., 2015	NA	Portugal	1998	Nasal swab	35	1	A	2.49	321	59	99.81	0.09	99.21	No
HFA6096	Meric et al., 2015	NA	Portugal	2000	Nasal swab	184	1	A	2.47	137	57	99.25	0.11	99.51	No
HFA6162	Meric et al., 2015	NA	Portugal	2000	Nasal swab	57	6	A	2.58	130	70	99.81	0	99.42	No
HFA6173B	Meric et al., 2015	NA	Portugal	2000	Nasal swab	1024	4	B	2.58	182	84	99.67	0	96.76	No
HFA6181	Meric et al., 2015	NA	Portugal	2001	Nasal swab	17	6	A	2.46	119	96	99.81	0	99.51	No
HFA6226	Meric et al., 2015	NA	Portugal	2001	Nasal swab	60	1	A	2.48	157	79	99.56	0.28	99.33	No
HFA6228	Meric et al., 2015	NA	Portugal	2001	Nasal swab	22	5	A	2.47	88	79	99.81	0.02	99.37	No
HFA6286	Meric et al., 2015	NA	Portugal	2001	Nasal swab	88	1	A	2.45	151	70	99.81	0	99.47	No
HFA6391	Meric et al., 2015	NA	Portugal	2001	Nasal swab	73	1	A	2.43	151	67	99.67	0.28	99.45	No
GCA_014857135	NCBI SSB	NA	Russia	Unknown	NA	1044	3	C	2.54	230	108	98.99	0.65	98.18	Yes
GCF_000390365	NCBI SSB	NA	Russia	Unknown	NA	2	5	A	2.69	64	100	99.65	0	99.34	No
GCF_015221215	NCBI SSB	NA	South Africa	Unknown	blood	490	3	C	2.41	348.11	22	99.67	0	98.25	Yes

GCF_015221265	NCBI SSB		NA	South Africa	Unknown	blood	490	3	A	2.64	126.61	103	99.81	0.43	99.44	No
GCF_015698585	NCBI SSB		NA	South Africa	Unknown	blood	59	6	A	2.57	94.19	53	99.81	0	99.39	No
GCF_015698595	NCBI SSB		NA	South Africa	Unknown	blood	54	1	A	2.89	53.31	177	99.76	1.54	99.32	No
GCF_003856395	NCBI SSB		NA	South Korea	Unknown	skin	20	1	A	2.57	2478	4	99.25	0.02	99.10	Yes
GCF_003856455	NCBI SSB		NA	South Korea	Unknown	toluene treated bioreactor sludge	20	1	A	2.57	2525	3	99.81	0.02	99.15	No
GCF_009769015	NCBI SSB		NA	South Korea	Unknown	Vagina	198	5	A	2.44	2444	1	99.81	0	99.49	Yes
GCF_009769125	NCBI SSB		NA	South Korea	Unknown	blood	673	3	C	2.56	2504	2	99.67	0.22	98.01	No
GCF_016834475	NCBI SSB		NA	South Korea	Unknown	skin	183	3	A	2.54	2486.89	4	99.81	0.02	99.49	No
GCF_016834495	NCBI SSB		NA	South Korea	Unknown	NA	218	1	A	2.56	2509.27	4	99.81	0	99.39	No
GCF_022591335	NCBI SSB		NA	South Korea	Unknown	skin	5	6	A	2.58	2511.01	4	99.72	0	99.56	No
JCT0400	In-house	JBJHQI000000000	CCARM 3A673	South Korea	Unknown	NA	1148	4	B	2.75	2585.549	8	99.39	2.64	96.76	Yes
JCT0401	In-house	JBICQX000000000	CCARM 3A593	South Korea	Unknown	NA	1094	4	B	2.60	509.713	13	97.85	0	96.80	Yes
JCT0402	In-house	CP170661-CP170662	CCARM 3A686	South Korea	Unknown	NA	1145	4	B	2.57	2561.483	2	99.11	0.28	96.78	Yes
JCT0403	In-house	CP170244	CCARM 3A616	South Korea	Unknown	NA	763	2	B	2.50	2497.149	1	99.52	0	96.87	Yes
JCT0404	In-house	CP170659-CP170660	CCARM 3A600	South Korea	Unknown	NA	1146	4	B	2.49	2467.735	2	99.65	0	96.88	Yes
JCT0372	In-house	CP170251	PS08	Spain	Unknown	NA	1141	4	B	2.49	2485.174	1	99.67	0	96.80	Yes
JCT0373	In-house	CP170250	PS21	Spain	Unknown	NA	1142	4	B	2.42	2417.268	1	99.61	0	96.88	Yes
JCT0374	In-house	CP170249	HS01	Spain	Unknown	NA	332	4	B	2.50	2498.113	1	99.64	0	96.85	Yes
JCT0375	In-house	CP170248	PS25	Spain	Unknown	NA	1006	4	B	2.40	2403.127	1	99.54	0	96.88	Yes
JCT0376	In-house	CP170247	Q10	Spain	Unknown	NA	368	4	B	2.55	2554.19	1	99.67	0.09	96.83	Yes
JCT0377	In-house	CP170246	Q8	Spain	Unknown	NA	1143	4	B	2.54	2541.778	1	99.67	0.02	96.80	Yes
JCT0378	In-house	CP170245	Q17	Spain	Unknown	NA	1144	4	B	2.47	2465.548	1	99.58	0.09	96.93	Yes
GCF_021728775	NCBI SSB		NA	Sudan	Unknown	Sepsis	73	1	B	2.47	208.56	28	99.67	0	96.95	No
GCF_021728835	NCBI SSB		NA	Sudan	Unknown	Table surface	369	4	A	2.51	165.07	45	99.81	0.09	99.46	No
1153_N002_1	PubMLST		NA	Sweden	Unknown	NA	73	1	A	2.47	96	110	99.67	0.28	99.41	No
1154_N13424T	PubMLST		NA	Sweden	Unknown	NA	712	1	A	2.53	88	110	99.81	0.37	99.54	No
1155_N13670T	PubMLST		NA	Sweden	Unknown	NA	713	6	A	2.49	83	141	99.81	0.56	99.42	No
1156_N13018T	PubMLST		NA	Sweden	Unknown	NA	714	6	A	2.45	107	84	99.81	0	99.38	No
1157_N14195T	PubMLST		NA	Sweden	Unknown	NA	715	1	A	2.45	83	107	99.81	0	99.63	No
1158_N14210T	PubMLST		NA	Sweden	Unknown	NA	716	6	A	2.44	98	101	99.81	0	99.58	No
1159_N14	PubMLST		NA	Sweden	Unknown	NA	717	1	A	2.50	102	91	99.63	0.1	99.55	No
1160_N15007T	PubMLST		NA	Sweden	Unknown	NA	718	5	A	2.41	92	114	99.81	0.91	99.44	Yes
1161_N15023T	PubMLST		NA	Sweden	Unknown	NA	719	4	B	2.56	118	101	99.63	0	97.08	No
1162_N17008T	PubMLST		NA	Sweden	Unknown	NA	720	6	A	2.42	96	95	99.81	0	99.57	No
1163_N17010T	PubMLST		NA	Sweden	Unknown	NA	721	1	A	2.47	81	107	99.67	0.28	99.46	No
1164_N17061T	PubMLST		NA	Sweden	Unknown	NA	637	6	A	2.47	111	122	99.81	0	99.47	No
1165_N17064T	PubMLST		NA	Sweden	Unknown	NA	723	4	B	2.61	137	162	99.67	0.08	97.61	No
1166_N17082T	PubMLST		NA	Sweden	Unknown	NA	673	3	C	2.51	162	60	99.67	0.22	98.04	Yes
1167_N177	PubMLST		NA	Sweden	Unknown	NA	725	1	A	2.45	96	82	99.81	0	99.38	No
1168_N33_1	PubMLST		NA	Sweden	Unknown	NA	726	6	A	2.47	96	79	99.81	0.56	99.41	No
1169_N415_1	PubMLST		NA	Sweden	Unknown	NA	727	6	A	2.45	79	97	99.81	0	99.22	No
1170_N74_3	PubMLST		NA	Sweden	Unknown	NA	728	4	B	2.46	179	61	99.67	0	96.84	No
1171_P155	PubMLST		NA	Sweden	Unknown	NA	Incomplete	#N/A	A	2.38	101	90	98.11	0.28	99.39	No
1172_P166355T	PubMLST		NA	Sweden	Unknown	NA	729	6	A	2.46	96	85	99.81	0	99.60	No
1174_P174	PubMLST		NA	Sweden	Unknown	NA	730	1	A	2.59	84	116	99.81	0.28	99.31	No
1175_P182	PubMLST		NA	Sweden	Unknown	NA	731	5	A	2.59	83	88	99.81	0.09	99.43	No
1176_P198	PubMLST		NA	Sweden	Unknown	NA	732	1	A	2.58	96	106	99.81	0.19	99.55	No
1177_P202T	PubMLST		NA	Sweden	Unknown	NA	733	1	A	2.48	83	153	99.81	0.28	99.35	No
1178_P203	PubMLST		NA	Sweden	Unknown	NA	734	6	A	2.39	98	64	99.81	0	99.42	No
1179_P215	PubMLST		NA	Sweden	Unknown	NA	735	5	A	2.59	83	82	99.81	0	99.33	No
1180_P219	PubMLST		NA	Sweden	Unknown	NA	736	6	A	2.56	96	86	99.81	0	99.46	No
1181_P224	PubMLST		NA	Sweden	Unknown	NA	737	6	A	2.48	96	99	99.81	0.02	99.40	No
1182_P287	PubMLST		NA	Sweden	Unknown	NA	738	2	B	2.48	120	70	99.67	0	97.38	Yes
1433_13_538_3	PubMLST		NA	Sweden	2013	NA	881	6	A	2.50	99	119	99.81	0	99.29	No
1434_13_613_3	PubMLST		NA	Sweden	2013	NA	882	5	A	2.55	79	157	99.81	0.17	99.33	No
1526_55_122_201	PubMLST		NA	Sweden	2011	NA	964	6	A	2.42	58	72	99.81	0.06	99.49	No
1592_20TYP36	PubMLST		NA	Sweden	2020	Community nasal swab	2	5	A	2.48	145.60	128	99.67	0	99.34	Yes
1593_20TYP27	PubMLST		NA	Sweden	2020	Community nasal swab	1003	1	A	2.47	145.42	102	99.81	0.28	99.39	No
1672_15MAS003558	PubMLST		NA	Sweden	2015	Animal mastitis	1063	4	A	2.41	124.00	96	99.81	0	99.57	No
1673_Se_tank38	PubMLST		NA	Sweden	2020	Milk	1064	6	A	2.52	184.66	75	99.81	0.56	99.36	No
1674_14MAS017450	PubMLST		NA	Sweden	2014	Animal mastitis	1065	6	A	2.43	176.96	103	99.81	0.28	99.41	No
1675_Se_tank58	PubMLST		NA	Sweden	2020	Milk	1066	6	A	2.45	122.88	107	99.11	0.56	99.41	No
1676_14MAS018426	PubMLST		NA	Sweden	2014	Animal mastitis	1067	1	A	2.40	148.40	115	99.81	0	99.52	No
1677_Se_tank46	PubMLST		NA	Sweden	2020	Milk	1068	1	A	2.44	168.41	90	99.81	0.28	99.48	No
1678_Se_tank31	PubMLST		NA	Sweden	2020	Milk	1069	6	A	2.54	135.65	111	99.81	0.37	99.45	No
1679_15MAS001437	PubMLST		NA	Sweden	2015	Animal mastitis	1070	6	A	2.43	146.89	79	99.76	0.37	99.47	Yes
1680_14MAS018417	PubMLST		NA	Sweden	2014	Animal mastitis	1071	1	A	2.40	120.96	157	99.81	1.26	99.38	No
GCF_022919195	NCBI SSB		NA	Switzerland	Unknown	Bloodstream	8	6	A	2.54	2539.08	1	99.81	0.66	99.54	Yes
GCF_015645075	NCBI SSB		NA	Taiwan	Unknown	cerebrospinal fluid	332	4	A	2.45	153.90	51	99.81	0	99.42	No
GCF_015645085	NCBI SSB		NA	Taiwan	Unknown	cerebrospinal fluid	59	6	A	2.53	180.99	55	99.81	0.06	99.32	No
GCF_015645175	NCBI SSB		NA	Taiwan	Unknown	blood	35	1	A	2.52	181.08	57	99.81	0.06	99.34	No
GCF_015645215	NCBI SSB		NA	Taiwan	Unknown	blood	35	1	A	2.45	153.90	55	99.81	0	99.46	No
TAW113	Meric et al., 2015		NA	Taiwan	1997	Sputum	85	1	A	2.49	149	60	99.81	0	99.57	No
TAW60	Meric et al., 2015		NA	Taiwan	1997	Sputum	59	6	A	2.45	110	62	99.81	0.28	99.58	No
1000_SS_0783	Meric et al., 2018		NA	UK	2012	Forearm	14	1	A	2.54	91	118	99.81	0.28	99.41	No
1001_SS_0784	Meric et al., 2018		NA	UK	2012	Retroauricular crease	130	6	A	2.50	146	70	99.81	0	99.50	No
1003_SS_0786	Meric et al., 2018		NA	UK	2012	Retroauricular crease	73	1	A	2.44	197	94	99.67	0.28	99.42	No
1004_SS_0787	Meric et al., 2018		NA	UK	2012	Retroauricular crease	59	6	A	2.42	124	90	99.81	0.19	99.62	No
1005_SS_0788	Meric et al., 2018		NA	UK	2012	Retroauricular crease	631	1	A	2.44	162	80	99.81	0.09	99.43	No
1008_SS_0791	Meric et al., 2018		NA	UK	2012	Retroauricular crease	1028	1	A	2.48	100	93	99.67	0.28	99.42	No
1011_SS_0794	Meric et al., 2018		NA	UK	2012	Retroauricular crease	73	1	A	2.47	145	81	99.67	0.28	99.49	No
1015_SS_0798	Meric et al., 2018		NA	UK	2012	Retroauricular crease	73	1	A	2.49	204	88	99.67	0.34	99.40	No
1016_SS_0799	Meric et al., 2018		NA	UK	2012	Axilla	378	1	A	2.50	133	96	99.81	0	99.47	Yes
1018_SS_0801	Meric et al., 2018		NA	UK	2012	Retroauricular crease	153	6	A	2.53	141	132	99.81	0.1	99.42	No
1019_SS_0802	Meric et al., 2018		NA	UK	2012	Retroauricular crease	14	1	A	2.57	185	101	99.81	0	99.41	No
1020_SS_0803	Meric et al., 2018		NA	UK	2012	Retroauricular crease	1012	4	B	2.55	333	93	99.67	0.03	97.72	No
1021_SS_0804	Meric et al., 2018		NA	UK	2012	Retroauricular crease	19	3	C	2.46	725	43	99.67	0.28	98.18	No

1022_SS_0805	Meric et al., 2018	NA	UK	2012	Axilla	32	6	A	2.59	148	95	99.64	1.52	99.20	No
1025_SS_0808	Meric et al., 2018	NA	UK	2012	Retroauricular crease	1012	4	B	2.59	643	64	99.67	0	97.73	No
1027_SS_0810	Meric et al., 2018	NA	UK	2012	Retroauricular crease	719	4	B	2.54	327	80	99.67	0	97.06	Yes
1031_SS_0814	Meric et al., 2018	NA	UK	2012	Retroauricular crease	1029	1	A	2.52	130	82	99.67	0.56	99.48	No
1032_SS_0815	Meric et al., 2018	NA	UK	2012	Retroauricular crease	153	6	A	2.48	145	90	99.81	0	99.48	No
1034_SS_0817	Meric et al., 2018	NA	UK	2012	Isolated from healthy volunteer	73	1	A	2.56	90	143	99.67	0.28	99.42	No
1036_SS_0819	Meric et al., 2018	NA	UK	2012	Isolated from healthy volunteer	19	3	C	2.53	585	51	99.67	0.06	98.11	No
1037_SS_0820	Meric et al., 2018	NA	UK	2012	Retroauricular crease	54	1	A	2.55	345	73	99.81	0.08	99.34	No
1038_SS_0821	Meric et al., 2018	NA	UK	2012	Groin	558	4	B	2.57	239	83	99.67	0	96.79	No
1227_436	PubMLST	NA	UK	2018	NA	771	6	A	2.47	105	107	99.81	0.62	98.90	Yes
1228_664	PubMLST	NA	UK	Unknown	NA	779	6	A	2.54	181	116	99.81	0	99.38	No
603_SS_0468	Meric et al., 2018	NA	UK	2013	Nasal cavity	1038	6	A	2.59	134	117	99.53	0.28	99.60	No
604_SS_0469	Meric et al., 2018	NA	UK	2013	Nasal cavity	184	1	A	2.50	145	112	99.25	0.06	99.56	No
605_SS_0470	Meric et al., 2018	NA	UK	2013	Nasal cavity	1039	6	A	2.43	118	81	99.81	0.28	99.48	No
606_SS_0471	Meric et al., 2018	NA	UK	2013	Nasal cavity	130	6	A	2.46	181	77	99.81	0	99.46	No
607_SS_0472	Meric et al., 2018	NA	UK	2013	Nasal cavity	297	1	A	2.43	111	76	99.81	0	99.51	No
608_SS_0473	Meric et al., 2018	NA	UK	2013	Nasal cavity	171	3	C	2.52	1310	59	99.67	0	98.18	No
609_SS_0474	Meric et al., 2018	NA	UK	2013	Nasal cavity	73	1	A	2.49	151	73	99.67	0.28	99.42	No
610_SS_0475	Meric et al., 2018	NA	UK	2013	Nasal cavity	73	1	A	2.51	276	85	99.67	0.3	99.45	No
614_SS_0479	Meric et al., 2018	NA	UK	2013	Nasal cavity	14	1	A	2.56	142	125	99.81	0.31	99.39	No
784_SS_0730	Meric et al., 2018	NA	UK	2012	Axilla	1011	6	A	2.53	96	104	99.81	0.19	99.35	Yes
788_SS_0459	Meric et al., 2018	NA	UK	2013	Nasal cavity	60	1	A	2.52	127	108	99.67	0.38	99.40	Yes
790_SS_0461	Meric et al., 2018	NA	UK	2013	Nasal cavity	32	6	A	2.51	132	78	99.67	0.28	99.25	No
791_SS_0462	Meric et al., 2018	NA	UK	2013	Nasal cavity	5	6	A	2.51	134	59	99.81	0	99.66	No
793_SS_0464	Meric et al., 2018	NA	UK	2013	Nasal cavity	1001	1	A	2.46	141	92	99.81	0	99.45	No
796_SS_0467	Meric et al., 2018	NA	UK	2013	Nasal cavity	59	6	A	2.43	119	100	99.25	0	99.54	No
952_SS_0735	Meric et al., 2018	NA	UK	2012	Forearm	89	1	A	2.48	201	84	99.7	0.02	99.45	No
954_SS_0737	Meric et al., 2018	NA	UK	2012	Axilla	5	6	A	2.45	151	67	99.81	0	99.56	No
961_SS_0744	Meric et al., 2018	NA	UK	2012	Axilla	1041	1	A	2.48	128	94	99.81	0.56	99.45	No
963_SS_0746	Meric et al., 2018	NA	UK	2012	Forearm	88	1	A	2.48	121	93	99.81	0.58	99.37	No
969_SS_0752	Meric et al., 2018	NA	UK	2012	Retroauricular crease	130	6	A	2.48	166	75	99.81	0	99.41	No
973_SS_0756	Meric et al., 2018	NA	UK	2012	Retroauricular crease	204	1	A	2.47	175	74	99.81	1.12	99.34	No
975_SS_0758	Meric et al., 2018	NA	UK	2012	Retroauricular crease	1042	6	A	2.50	186	85	99.81	0	99.31	No
979_SS_0762	Meric et al., 2018	NA	UK	2012	Retroauricular crease	59	6	A	2.46	140	69	99.81	0	99.59	No
983_SS_0766	Meric et al., 2018	NA	UK	2012	Retroauricular crease	1043	4	B	2.51	361	66	99.67	0.06	96.74	No
984_SS_0767	Meric et al., 2018	NA	UK	2012	Retroauricular crease	73	1	A	2.52	133	119	99.67	0.9	99.41	No
986_SS_0769	Meric et al., 2018	NA	UK	2012	Axilla	190	6	A	2.45	158	112	99.81	0.19	99.93	No
988_SS_0771	Meric et al., 2018	NA	UK	2012	Retroauricular crease	89	1	A	2.49	119	85	99.81	0.02	99.52	No
990_SS_0773	Meric et al., 2018	NA	UK	2012	Retroauricular crease	32	6	A	2.48	121	86	99.67	0	99.16	No
994_SS_0777	Meric et al., 2018	NA	UK	2012	Retroauricular crease	1012	4	B	2.61	336	112	99.67	0	97.62	No
995_SS_0778	Meric et al., 2018	NA	UK	2012	Retroauricular crease	38	1	A	2.49	113	130	99.25	0.02	99.45	No
997_SS_0780	Meric et al., 2018	NA	UK	2012	Retroauricular crease	48	5	A	2.53	184	104	99.67	0.66	99.23	No
GCF_000966705	NCBI SSB	NA	UK	Unknown	blood	2	5	A	2.69	97	53	99.81	0.19	99.27	No
GCF_000966715	NCBI SSB	NA	UK	Unknown	blood	439	4	B	2.58	131	51	99.67	0	96.81	Yes
GCF_000966755	NCBI SSB	NA	UK	Unknown	blood	2	5	A	2.71	117	44	99.81	0.28	99.27	No
GCF_000966765	NCBI SSB	NA	UK	Unknown	blood	2	5	A	2.62	103	48	99.81	0.09	99.32	No
GCF_000966775	NCBI SSB	NA	UK	Unknown	blood	2	5	A	2.62	94	48	99.81	0.09	99.32	No
GCF_000966815	NCBI SSB	NA	UK	Unknown	blood	2	5	A	2.66	97	45	99.81	0.19	99.32	No
GCF_003859985	NCBI SSB	NA	UK	Unknown	dog mouth	130	6	A	2.50	71	102	99.81	0.09	99.42	No
GCF_008922045	NCBI SSB	NA	UK	Unknown	blood	2	5	A	2.65	107	133	99.81	1.23	99.21	No
GCF_008922095	NCBI SSB	NA	UK	Unknown	blood	48	5	A	2.53	184	66	99.67	0.86	99.29	No
GCF_008922115	NCBI SSB	NA	UK	Unknown	blood	2	5	A	2.80	80	179	99.81	1.37	99.12	No
GCF_008922155	NCBI SSB	NA	UK	Unknown	blood	2	5	A	2.46	169	64	99.67	0	99.27	Yes
GCF_008922165	NCBI SSB	NA	UK	Unknown	blood	2	5	A	2.73	94	60	99.81	0	99.19	No
GCF_008922175	NCBI SSB	NA	UK	Unknown	blood	204	1	A	2.51	270	87	99.81	0.57	99.32	Yes
GCF_008922205	NCBI SSB	NA	UK	Unknown	blood	2	5	A	2.57	150	111	99.81	1.2	99.44	No
GCF_008922245	NCBI SSB	NA	UK	Unknown	blood	19	3	C	2.44	1330	22	99.67	0	98.19	No
GCF_008922285	NCBI SSB	NA	UK	Unknown	blood	2	5	A	2.72	110	71	99.81	0	99.10	No
GCF_008922315	NCBI SSB	NA	UK	Unknown	blood	2	5	A	2.74	91	69	99.81	0	99.25	No
GCF_008922345	NCBI SSB	NA	UK	Unknown	blood	83	6	A	2.55	143	70	99.81	0.63	99.34	No
GCF_008922365	NCBI SSB	NA	UK	Unknown	blood	2	5	A	2.60	150	79	99.81	0.44	99.42	No
GCF_008922375	NCBI SSB	NA	UK	Unknown	blood	5	6	A	2.54	97	128	99.7	0.04	99.51	No
GCF_008922415	NCBI SSB	NA	UK	Unknown	blood	5	6	A	2.54	110	61	99.81	0	99.51	No
GCF_008922445	NCBI SSB	NA	UK	Unknown	blood	2	5	A	2.78	94	142	99.81	0.66	99.11	No
GCF_008922455	NCBI SSB	NA	UK	Unknown	blood	559	4	B	2.61	205	83	99.67	0.92	96.78	Yes
GCF_008922485	NCBI SSB	NA	UK	Unknown	blood	48	5	A	2.44	166	51	99.67	0.28	99.23	No
GCF_008922505	NCBI SSB	NA	UK	Unknown	blood	48	5	A	2.43	166	45	99.67	0.28	99.31	No
GCF_008922525	NCBI SSB	NA	UK	Unknown	blood	2	5	A	2.60	83	66	99.81	0.09	99.35	No
GCF_008922545	NCBI SSB	NA	UK	Unknown	blood	2	5	A	2.60	82	70	99.81	0.09	99.24	No
GCF_008922555	NCBI SSB	NA	UK	Unknown	blood	2	5	A	2.57	82	70	99.81	0	99.37	No
GCF_008922565	NCBI SSB	NA	UK	Unknown	blood	2	5	A	2.57	102	67	99.81	0	99.47	No
GCF_008922605	NCBI SSB	NA	UK	Unknown	blood	2	5	A	2.53	128	56	99.81	0	99.44	No
GCF_008922645	NCBI SSB	NA	UK	Unknown	blood	2	5	A	2.60	102	68	99.81	0.09	99.25	No
GCF_008922655	NCBI SSB	NA	UK	Unknown	blood	2	5	A	2.60	82	64	99.81	0.09	99.31	No
GCF_008922665	NCBI SSB	NA	UK	Unknown	blood	915	1	A	2.48	108	55	99.81	0.04	99.38	No
GCF_008922695	NCBI SSB	NA	UK	Unknown	blood	2	5	A	2.57	82	69	99.81	0	99.28	No
GCF_008922745	NCBI SSB	NA	UK	Unknown	blood	2	5	A	2.61	82	64	99.81	0.09	99.29	No
GCF_008922755	NCBI SSB	NA	UK	Unknown	blood	210	6	A	2.47	145	63	99.81	0	99.43	No
GCF_008922785	NCBI SSB	NA	UK	Unknown	blood	73	1	A	2.49	102	57	99.67	0.34	99.39	No
GCF_008922795	NCBI SSB	NA	UK	Unknown	blood	2	5	A	2.57	83	65	99.81	0	99.24	No
GCF_008922805	NCBI SSB	NA	UK	Unknown	blood	1022	3	C	2.56	293	31	99.67	0.02	98.44	Yes
GCF_008922845	NCBI SSB	NA	UK	Unknown	blood	210	6	A	2.53	145	67	99.81	0.02	99.40	No
GCF_008922855	NCBI SSB	NA	UK	Unknown	blood	54	1	A	2.51	143	61	99.81	0.02	99.31	No
GCF_008922885	NCBI SSB	NA	UK	Unknown	blood	2	5	A	2.60	82	66	99.81	0.09	99.25	No
GCF_008922895	NCBI SSB	NA	UK	Unknown	blood	2	5	A	2.60	82	68	99.81	0.09	99.24	No
GCF_008922925	NCBI SSB	NA	UK	Unknown	blood	2	5	A	2.60	82	62	99.81	0.09	99.28	No
GCF_008922945	NCBI SSB	NA	UK	Unknown	blood	528	6	A	2.52	75	70	99.81	0	99.18	No

GCF_008922955	NCBI SSB	NA	UK	Unknown	blood	2	5	A	2.73	82	69	99.81	0	99.13	No
GCF_008922985	NCBI SSB	NA	UK	Unknown	blood	23	5	A	2.46	82	65	99.81	0.02	99.14	No
GCF_008922995	NCBI SSB	NA	UK	Unknown	blood	2	5	A	2.72	82	65	99.81	0	99.17	No
GCF_008923005	NCBI SSB	NA	UK	Unknown	blood	2	5	A	2.59	103	67	99.81	0	99.47	No
GCF_008923045	NCBI SSB	NA	UK	Unknown	blood	5	6	A	2.43	96	64	99.81	0	99.54	No
GCF_008923065	NCBI SSB	NA	UK	Unknown	blood	5	6	A	2.43	96	63	99.81	0	99.53	No
GCF_008923075	NCBI SSB	NA	UK	Unknown	blood	59	6	A	2.43	123	47	99.81	0	99.57	No
GCF_008923085	NCBI SSB	NA	UK	Unknown	blood	48	5	A	2.51	166	59	99.67	0.37	99.22	No
GCF_008923125	NCBI SSB	NA	UK	Unknown	blood	35	1	A	2.45	122	50	99.81	0	99.35	No
GCF_008923145	NCBI SSB	NA	UK	Unknown	blood	2	5	A	2.73	83	68	99.81	0	99.18	No
GCF_009026045	NCBI SSB	NA	UK	Unknown	blood	2	5	A	2.58	122	76	99.81	0.04	99.27	No
GCF_009026065	NCBI SSB	NA	UK	Unknown	blood	54	1	A	2.54	227	124	99.81	1.13	99.40	No
GCF_009757165	NCBI SSB	NA	UK	Unknown	feces	5	6	A	2.47	226	50	99.81	0.56	99.58	No
GCF_018407685	NCBI SSB	NA	UK	Unknown	NA	487	6	A	2.49	180.36	33	99.81	0.08	99.34	No
GCF_018407805	NCBI SSB	NA	UK	Unknown	NA	54	1	A	2.45	136.98	42	99.81	0	99.47	No
GCF_018407885	NCBI SSB	NA	UK	Unknown	NA	1155	6	A	2.65	180.04	39	99.81	0.51	99.35	No
GCF_018407945	NCBI SSB	NA	UK	Unknown	NA	35	1	A	2.48	96.15	52	99.81	0.28	99.87	No
GCF_018408135	NCBI SSB	NA	UK	Unknown	NA	190	6	A	2.52	331.39	28	99.81	0.51	99.37	No
URU23	Meric et al., 2015	NA	Uraguay	1997	Urine	86	1	A	2.67	127	85	99.81	0	99.34	No
1422_117_1_305	PubMLST	NA	USA	Unknown	bacteraemia	877	4	B	2.52	160	50	99.67	0	96.80	Yes
1423_123_1_323	PubMLST	NA	USA	Unknown	bacteraemia	878	3	C	2.51	168	46	99.67	0.42	98.15	Yes
1424_145_1_361	PubMLST	NA	USA	Unknown	bacteraemia	879	6	A	2.46	102	60	99.81	0	99.48	No
1425_166_2_374	PubMLST	NA	USA	Unknown	bacteraemia	880	4	B	2.48	197	28	99.67	0	96.85	No
1426_23_1_76	PubMLST	NA	USA	Unknown	bacteraemia	865	3	C	2.56	250	47	99.67	0.48	98.06	Yes
1427_52_1_150	PubMLST	NA	USA	Unknown	bacteraemia	768	4	B	2.36	210	36	99.63	0	96.84	No
1428_67_1_191	PubMLST	NA	USA	Unknown	bacteraemia	883	1	A	2.49	111	53	99.81	0.06	99.42	No
1429_8_1_17	PubMLST	NA	USA	Unknown	bacteraemia	764	4	B	2.46	177	44	99.67	0.56	96.88	No
1430_163_2_371	PubMLST	NA	USA	Unknown	bacteraemia	885	4	B	2.49	108	79	99.67	0	96.83	No
1431_165_2_373	PubMLST	NA	USA	Unknown	bacteraemia	886	1	A	2.42	103	54	99.81	0.56	99.36	Yes
1432_37_1_109	PubMLST	NA	USA	Unknown	bacteraemia	887	4	B	2.54	188	37	99.63	0	96.82	No
1435_48_1_138	PubMLST	NA	USA	Unknown	bacteraemia	884	4	B	2.53	96	62	99.67	0	96.79	No
GCA_018373255	NCBI SSB	NA	USA	Unknown	Infant faeces	1151	4	A	2.36	81.08	57	99.81	0	99.52	No
GCA_019693595	NCBI SSB	NA	USA	Unknown	Cerebrospinal fluid	210	6	A	2.39	67.50	67	95.95	0	99.33	No
GCF_000275965	NCBI SSB	NA	USA	Unknown	gluteal crease	89	1	A	2.51	82	91	99.86	0.71	98.87	No
GCF_000276005	NCBI SSB	NA	USA	Unknown	nare	430	6	A	2.49	95	61	99.81	0	99.52	No
GCF_000276045	NCBI SSB	NA	USA	Unknown	axilla	5	6	A	2.62	89	76	99.81	0.36	99.47	No
GCF_000276065	NCBI SSB	NA	USA	Unknown	nare	5	6	A	2.51	89	68	99.81	0.66	99.46	No
GCF_000276385	NCBI SSB	NA	USA	Unknown	blood	330	1	A	2.48	126	56	99.77	0.62	99.36	No
GCF_000276465	NCBI SSB	NA	USA	Unknown	blood	333	1	A	2.40	122	51	99.81	0	99.47	No
GCF_000783535	NCBI SSB	NA	USA	Unknown	NA	69	6	A	2.58	2538	3	99.81	0.62	99.38	No
GCF_001061715	NCBI SSB	NA	USA	Unknown	NA	2	5	A	2.70	65	122	99.81	0	99.41	No
GCF_001066085	NCBI SSB	NA	USA	Unknown	NA	1047	1	A	2.47	70	139	99.81	0	99.40	No
GCF_001068455	NCBI SSB	NA	USA	Unknown	NA	2	5	A	2.61	62	143	99.81	0.66	99.34	No
GCF_001068505	NCBI SSB	NA	USA	Unknown	NA	2	5	A	2.55	53	143	99.81	0.02	99.48	No
GCF_001068895	NCBI SSB	NA	USA	Unknown	NA	2	5	A	2.52	56	123	99.81	0.12	99.40	No
GCF_001069515	NCBI SSB	NA	USA	Unknown	NA	2	5	A	2.62	63	113	99.81	0.09	99.37	No
GCF_001069955	NCBI SSB	NA	USA	Unknown	NA	2	5	A	2.51	50	140	99.81	0.3	99.40	No
GCF_001069965	NCBI SSB	NA	USA	Unknown	NA	2	5	A	2.49	51	143	99.81	0.4	99.51	No
GCF_001069995	NCBI SSB	NA	USA	Unknown	NA	130	6	A	2.48	65	172	99.81	0.95	99.35	No
GCF_001070075	NCBI SSB	NA	USA	Unknown	NA	2	5	A	2.57	56	131	99.81	0.4	99.39	No
GCF_001070155	NCBI SSB	NA	USA	Unknown	NA	5	6	A	2.44	62	127	99.81	0	99.46	No
GCF_001070245	NCBI SSB	NA	USA	Unknown	NA	88	1	A	2.44	81	107	99.7	0	99.55	No
GCF_001070485	NCBI SSB	NA	USA	Unknown	NA	17	6	A	2.42	62	124	99.81	0.08	99.53	No
GCF_001070505	NCBI SSB	NA	USA	Unknown	NA	17	6	A	2.43	63	116	99.81	0.08	99.44	No
GCF_001070735	NCBI SSB	NA	USA	Unknown	NA	1048	5	A	2.54	67	86	99.81	0.09	99.27	No
GCF_001071895	NCBI SSB	NA	USA	Unknown	NA	5	6	A	2.39	70	113	99.81	0	99.52	No
GCF_001071935	NCBI SSB	NA	USA	Unknown	NA	35	1	A	2.45	82	96	99.81	0.62	99.35	No
GCF_001072715	NCBI SSB	NA	USA	Unknown	NA	210	6	A	2.45	56	130	99.81	0	99.48	No
GCF_001073435	NCBI SSB	NA	USA	Unknown	NA	2	5	A	2.61	77	90	99.81	0.4	99.42	No
GCF_001073515	NCBI SSB	NA	USA	Unknown	NA	73	1	A	2.48	70	126	99.67	0.34	99.44	No
GCF_001073875	NCBI SSB	NA	USA	Unknown	NA	218	1	A	2.41	61	120	99.81	0.19	99.54	No
GCF_001073995	NCBI SSB	NA	USA	Unknown	NA	487	6	A	2.39	58	121	99.81	0.19	99.41	No
GCF_001074495	NCBI SSB	NA	USA	Unknown	NA	2	5	A	2.53	54	117	99.81	0	99.42	No
GCF_001074505	NCBI SSB	NA	USA	Unknown	NA	847	5	A	2.59	61	132	99.81	0.09	99.32	No
GCF_001074755	NCBI SSB	NA	USA	Unknown	NA	2	5	A	2.59	54	149	99.81	0.09	99.37	No
GCF_001075165	NCBI SSB	NA	USA	Unknown	NA	7	6	A	2.44	64	108	99.81	0.02	99.45	No
GCF_001075235	NCBI SSB	NA	USA	Unknown	NA	5	6	A	2.54	58	161	99.81	0.94	99.51	No
GCF_001075405	NCBI SSB	NA	USA	Unknown	NA	5	6	A	2.49	60	131	99.81	0	99.38	No
GCF_001075555	NCBI SSB	NA	USA	Unknown	NA	83	6	A	2.52	61	110	99.81	0	99.31	No
GCF_001075575	NCBI SSB	NA	USA	Unknown	NA	83	6	A	2.51	61	120	99.81	0	99.33	No
GCF_001075605	NCBI SSB	NA	USA	Unknown	NA	69	6	A	2.50	73	101	99.81	0	99.36	No
GCF_001075835	NCBI SSB	NA	USA	Unknown	NA	2	5	A	2.61	63	122	99.81	0.09	99.46	No
GCF_001473265	NCBI SSB	NA	USA	Unknown	Peripheral blood	20	1	A	2.60	2542	3	99.81	0	99.04	No
GCF_002215535	NCBI SSB	ATCC 12228	USA	Unknown	skin and mucous membranes	679	4	B	2.57	2498	6	99.39	0	96.82	No
GCF_002221915	NCBI SSB	NA	USA	Unknown	NA	1051	6	A	2.53	81	87	99.81	0	99.47	No
GCF_002221945	NCBI SSB	NA	USA	Unknown	NA	22	5	A	2.41	93	59	99.81	0.02	99.47	No
GCF_002417645	NCBI SSB	NA	USA	Unknown	NA	351	6	A	2.48	104	62	99.81	0.1	99.43	No
GCF_002750225	NCBI SSB	NA	USA	Unknown	Neck/Axilla	2	5	A	2.62	112	77	99.81	0.09	99.34	No
GCF_002850315	NCBI SSB	NA	USA	Unknown	Blood	2	5	A	2.73	2728	1	99.81	0	99.45	Yes
GCF_002944995	NCBI SSB	NA	USA	Unknown	Peripheral blood	5	6	A	2.55	2498	5	99.81	0	99.50	No
GCF_002954055	NCBI SSB	NA	USA	Unknown	Peripheral blood	20	1	A	2.52	2485	3	99.81	0.1	99.19	No
GCF_003725355	NCBI SSB	NA	USA	Unknown	stomach biopsy	1019	4	B	2.48	127	49	99.67	0.1	96.84	No
GCF_006151885	NCBI SSB	NA	USA	Unknown	stomach	73	1	A	2.57	232	55	99.67	0.06	99.32	No
GCF_007678685	NCBI SSB	NA	USA	Unknown	environmental	16	1	A	2.50	151	70	99.81	0	99.31	No
GCF_008726255	NCBI SSB	NA	USA	Unknown	NA	7	6	A	2.44	66	60	99.81	0.28	99.46	No
GCF_008726465	NCBI SSB	NA	USA	Unknown	NA	88	1	A	2.44	51	98	99.7	0.23	99.48	No
GCF_008726735	NCBI SSB	NA	USA	Unknown	NA	1020	6	A	2.48	91	61	99.81	0.78	99.40	No

GCF_008726775	NCBI SSB	NA	USA	Unknown	NA	167	6	A	2.43	90	57	99.81	0.56	99.38	No
GCF_008726905	NCBI SSB	NA	USA	Unknown	NA	32	6	A	2.45	84	45	99.67	0	99.27	No
GCF_008727015	NCBI SSB	NA	USA	Unknown	NA	32	6	A	2.48	120	41	99.67	0	99.24	No
GCF_009765515	NCBI SSB	NA	USA	Unknown	Precipitation	1012	4	B	2.46	77	98	99.67	0	97.73	No
GCF_010132335	NCBI SSB	NA	USA	Unknown	mobile phone	130	6	A	2.58	2491	4	99.56	0.42	99.38	No
GCF_012029805	NCBI SSB	NA	USA	Unknown	NA	323	6	A	2.54	88	61	99.81	0.1	99.29	No
GCF_012030625	NCBI SSB	NA	USA	Unknown	NA	487	6	A	2.53	158	68	99.81	0.73	99.36	No
GCF_012641685	NCBI SSB	NA	USA	Unknown	toothbrush	59	6	A	2.52	71	120	99.81	0.06	99.44	No
GCF_014334295	NCBI SSB	NA	USA	Unknown	Forehead	72	1	C	2.49	2494	1	99.67	0	98.37	No
GCF_016808595	NCBI SSB	NA	USA	Unknown	air	626	6	A	2.33	102.15	42	98.13	0.36	99.42	No
GCF_016808675	NCBI SSB	NA	USA	Unknown	air	329	4	B	2.38	281.54	20	99.67	0	96.88	No
GCF_016808755	NCBI SSB	NA	USA	Unknown	air	329	4	C	2.57	151.95	32	99.67	0.06	98.07	No
GCF_016809015	NCBI SSB	NA	USA	Unknown	air	183	3	A	2.41	96.10	46	99.81	0	99.43	Yes
GCF_016809035	NCBI SSB	NA	USA	Unknown	air	153	6	B	2.49	167.30	26	99.67	0	96.81	No
GCF_016809275	NCBI SSB	NA	USA	Unknown	air	1019	4	A	2.44	96.26	47	99.81	0	99.35	No
GCF_016809325	NCBI SSB	NA	USA	Unknown	air	17	6	C	2.52	126.88	30	99.67	0.11	98.09	No
GCF_016889165	NCBI SSB	NA	USA	Unknown	NA	130	6	A	2.53	2470.06	7	99.81	0.09	99.41	No
GCF_016903555	NCBI SSB	NA	USA	Unknown	NA	487	6	A	2.51	2470.07	4	99.81	0.09	99.45	No
GCF_016903575	NCBI SSB	NA	USA	Unknown	NA	487	6	A	2.45	2389.13	3	99.81	0.28	98.98	Yes
GCF_016904135	NCBI SSB	NA	USA	Unknown	NA	1154	1	A	2.52	2436.42	5	99.81	0.2	99.40	No
GCF_018919325	NCBI SSB	NA	USA	Unknown	NA	35	1	A	2.43	129.95	42	99.81	0	99.51	No
GCF_018919425	NCBI SSB	NA	USA	Unknown	NA	57	6	A	2.48	169.06	36	99.81	0	99.40	No
GCF_021292185	NCBI SSB	NA	USA	Unknown	nematode growth plate	179	1	A	2.51	105.62	100	99.81	0.14	99.36	No
GCF_022352465	NCBI SSB	NA	USA	Unknown	NA	1170	4	A	2.64	140.00	63	99.81	0	99.50	No
GCF_022352485	NCBI SSB	NA	USA	Unknown	NA	22	5	A	2.52	98.48	52	99.81	0	99.47	No
GCF_022352535	NCBI SSB	NA	USA	Unknown	NA	5	6	B	2.51	426.85	31	99.67	0	96.81	No
GCF_022352605	NCBI SSB	NA	USA	Unknown	NA	329	4	A	2.54	169.26	46	99.81	0.06	99.44	No
GCF_022352615	NCBI SSB	NA	USA	Unknown	NA	59	6	A	2.48	120.97	54	99.81	0.28	99.52	No
GCF_022352685	NCBI SSB	NA	USA	Unknown	NA	5	6	B	2.55	281.70	37	99.67	0	96.83	No
GCF_022352705	NCBI SSB	NA	USA	Unknown	NA	290	4	A	2.53	120.35	43	99.67	0	99.25	No
GCF_022352725	NCBI SSB	NA	USA	Unknown	NA	32	6	A	2.59	211.19	46	99.81	0.06	99.43	No
GCF_022352745	NCBI SSB	NA	USA	Unknown	NA	59	6	C	2.47	1333.51	15	99.67	0	98.13	No
GCF_022352845	NCBI SSB	NA	USA	Unknown	NA	19	3	C	2.53	170.93	45	99.67	0	98.30	Yes
GCF_022352855	NCBI SSB	NA	USA	Unknown	NA	170	3	A	2.56	124.96	52	99.81	0	99.30	No
GCF_022352885	NCBI SSB	NA	USA	Unknown	NA	16	1	A	2.49	95.97	53	99.81	0	99.46	No
GCF_022352895	NCBI SSB	NA	USA	Unknown	NA	5	6	A	2.45	197.37	39	99.53	0.1	99.38	No
GCF_022352925	NCBI SSB	NA	USA	Unknown	NA	153	6	A	2.58	132.16	43	99.81	0	99.18	No
GCF_022352985	NCBI SSB	NA	USA	Unknown	NA	10	6	B	2.55	196.43	28	99.67	0	96.82	No
GCF_022353005	NCBI SSB	NA	USA	Unknown	NA	290	4	B	2.54	179.48	41	99.67	0	96.86	No
GCF_022353015	NCBI SSB	NA	USA	Unknown	NA	290	4	A	2.49	103.25	49	99.81	0	99.49	No
GCF_022353025	NCBI SSB	NA	USA	Unknown	NA	5	6	A	2.46	318.18	32	99.81	0	99.36	No
GCF_022353065	NCBI SSB	NA	USA	Unknown	NA	16	1	C	2.44	178.12	31	99.67	0	98.33	No
GCF_022353125	NCBI SSB	NA	USA	Unknown	NA	490	3	A	2.39	180.99	33	99.81	0	99.56	No
GCF_022353135	NCBI SSB	NA	USA	Unknown	NA	5	6	A	2.46	166.56	38	99.81	0.06	99.49	No
GCF_022426545	NCBI SSB	NA	USA	Unknown	NA	59	6	A	2.44	187.04	42	99.81	0.64	99.36	No
GCF_022428385	NCBI SSB	NA	USA	Unknown	NA	6	1	B	2.51	283.55	27	99.67	0	96.86	No
GCF_022428405	NCBI SSB	NA	USA	Unknown	NA	984	4	A	2.41	146.04	44	99.81	0.28	99.49	No

Table S2. Structural alignment data for haplotypes of each gene (defined only by sites under selection). Alignments were only generated for haplotypes with a combined BLOSUM62 score of less than zero.

Gene	Model 8 Sites: Major Allele	Haplotype	Haplotype Frequency	BLOSUM62 score	Alignment to most common haplotype (RMSD)
csd	321S	K	0.0140	-1	0.078
csd	321S	S	0.1037	1	
csd	321S	T	0.8644	5	
cysG	32S; 242I; 270R	ATR	0.0010	2	
cysG	32S; 242I; 270R	ASK	0.0050	4	
cysG	32S; 242I; 270R	ATK	0.0459	5	
cysG	32S; 242I; 270R	STR	0.0010	5	
cysG	32S; 242I; 270R	AIR	0.0020	7	
cysG	32S; 242I; 270R	SSK	0.0050	7	
cysG	32S; 242I; 270R	VIK	0.0010	7	
cysG	32S; 242I; 270R	STK	0.0010	8	
cysG	32S; 242I; 270R	AIK	0.0518	10	
cysG	32S; 242I; 270R	SIR	0.0229	10	
cysG	32S; 242I; 270R	SVK	0.0120	12	
cysG	32S; 242I; 270R	SIK	0.8335	13	
group_10276	107K; 167D	RC	0.0010	-1	0.271
group_10276	107K; 167D	RG	0.0060	1	
group_10276	107K; 167D	KG	0.0010	4	
group_10276	107K; 167D	ND	0.0010	6	
group_10276	107K; 167D	RD	0.0748	8	
group_10276	107K; 167D	KD	0.8983	11	
group_1172	66E; 160C; 202P	GRQ	0.0130	-6	0.171
group_1172	66E; 160C; 202P	GRS	0.0070	-6	0.133
group_1172	66E; 160C; 202P	RRS	0.0030	-4	0.177
group_1172	66E; 160C; 202P	EHL	0.0050	-1	0.133
group_1172	66E; 160C; 202P	ERS	0.0289	1	
group_1172	66E; 160C; 202P	GHP	0.0070	2	
group_1172	66E; 160C; 202P	GRP	0.0957	2	
group_1172	66E; 160C; 202P	RRP	0.0449	4	
group_1172	66E; 160C; 202P	ECP	0.7777	21	
group_1349	183Y; 195N; 213G; 218A; 243S	NNVVS	0.0010	-5	0.218
group_1349	183Y; 195N; 213G; 218A; 243S	NNLAS	0.0090	-2	0.196
group_1349	183Y; 195N; 213G; 218A; 243S	NNVAS	0.2752	-1	0.322
group_1349	183Y; 195N; 213G; 218A; 243S	NYVAS	0.0010	0	
group_1349	183Y; 195N; 213G; 218A; 243S	NNGVS	0.0120	4	
group_1349	183Y; 195N; 213G; 218A; 243S	NHVAS	0.0189	6	
group_1349	183Y; 195N; 213G; 218A; 243S	NNGAS	0.0030	8	
group_1349	183Y; 195N; 213G; 218A; 243S	YNVAS	0.0010	8	
group_1349	183Y; 195N; 213G; 218A; 243S	NHGVS	0.0010	11	
group_1349	183Y; 195N; 213G; 218A; 243S	YNGVS	0.0160	13	
group_1349	183Y; 195N; 213G; 218A; 243S	NHGAS	0.0080	15	
group_1349	183Y; 195N; 213G; 218A; 243S	NNGAH	0.0120	17	
group_1349	183Y; 195N; 213G; 218A; 243S	YNGAS	0.0160	17	
group_1349	183Y; 195N; 213G; 218A; 243S	YHGAP	0.0818	23	
group_1349	183Y; 195N; 213G; 218A; 243S	YHGAH	0.5264	33	
group_2443	151H; 156G; 214P; 552L; 584V	HEPLG	0.0070	-4	0.457
group_2443	151H; 156G; 214P; 552L; 584V	HGPLG	0.0010	-4	0.457
group_2443	151H; 156G; 214P; 552L; 584V	REPLG	0.0040	1	
group_2443	151H; 156G; 214P; 552L; 584V	HGPLV	0.0608	3	
group_2443	151H; 156G; 214P; 552L; 584V	PGPIV	0.0020	3	
group_2443	151H; 156G; 214P; 552L; 584V	HGPIV	0.0070	5	
group_2443	151H; 156G; 214P; 552L; 584V	RNPLG	0.0219	7	
group_2443	151H; 156G; 214P; 552L; 584V	RELLG	0.0020	8	
group_2443	151H; 156G; 214P; 552L; 584V	RGPLV	0.0538	8	
group_2443	151H; 156G; 214P; 552L; 584V	RNPLS	0.0070	8	
group_2443	151H; 156G; 214P; 552L; 584V	REPIV	0.0299	10	
group_2443	151H; 156G; 214P; 552L; 584V	RGPIV	0.0050	10	
group_2443	151H; 156G; 214P; 552L; 584V	RGTLV	0.0040	10	
group_2443	151H; 156G; 214P; 552L; 584V	RELIV	0.0110	17	
group_2443	151H; 156G; 214P; 552L; 584V	RNLIV	0.7657	23	
group_3177	463S	A	0.0768	1	
group_3177	463S	S	0.9053	4	
hisI	122D	D	0.0538	-2	0.951
hisI	122D	G	0.0020	0	
hisI	122D	S	0.4626	1	
hisI	122D	A	0.4636	4	
ileS	588V	F	0.1167	-1	0.561
ileS	588V	V	0.8654	4	
nasD	27Q; 757V	PI	0.0050	2	
nasD	27Q; 757V	TI	0.0010	2	
nasD	27Q; 757V	PV	0.0518	3	
nasD	27Q; 757V	QI	0.0309	8	
nasD	27Q; 757V	QV	0.8933	9	
ndhB	158R; 441L	RR	0.0030	-5	0.286
ndhB	158R; 441L	RS	0.0518	-5	0.174
ndhB	158R; 441L	RL	0.0528	1	
ndhB	158R; 441L	WR	0.0169	9	
ndhB	158R; 441L	WS	0.0090	9	

ndhB	158R; 441L	WL	0.8485	15	
rluD3	77V; 174A; 179R; 201R	IVLK	0.0020	3	
rluD3	77V; 174A; 179R; 201R	VVLK	0.0638	4	
rluD3	77V; 174A; 179R; 201R	IALK	0.0160	7	
rluD3	77V; 174A; 179R; 201R	VALK	0.0927	8	
rluD3	77V; 174A; 179R; 201R	IAHR	0.2672	12	
rluD3	77V; 174A; 179R; 201R	VERR	0.0668	13	
rluD3	77V; 174A; 179R; 201R	IARK	0.0130	14	
rluD3	77V; 174A; 179R; 201R	VTRR	0.0010	14	
rluD3	77V; 174A; 179R; 201R	VARK	0.0229	15	
rluD3	77V; 174A; 179R; 201R	IARR	0.0030	17	
rluD3	77V; 174A; 179R; 201R	VARR	0.4337	18	
sirC	54A; 89G	MK	0.0040	-3	0.473
sirC	54A; 89G	AK	0.0060	-2	0.511
sirC	54A; 89G	TR	0.0568	3	
sirC	54A; 89G	AG	0.0449	6	
sirC	54A; 89G	TG	0.8704	11	
yfcA	13V; 211F	VF	0.0060	-2	0.318
yfcA	13V; 211F	AF	0.1366	2	
yfcA	13V; 211F	AI	0.0020	2	
yfcA	13V; 211F	XS	0.0040	3	
yfcA	13V; 211F	TS	0.0169	4	
yfcA	13V; 211F	VS	0.0090	4	
yfcA	13V; 211F	AS	0.8076	8	
yheS	59H; 346R; 352V	RRV	0.0020	1	
yheS	59H; 346R; 352V	NRV	0.0010	2	
yheS	59H; 346R; 352V	RRI	0.0020	2	
yheS	59H; 346R; 352V	QLV	0.0329	7	
yheS	59H; 346R; 352V	RLV	0.0100	7	
yheS	59H; 346R; 352V	QLI	0.1047	8	
yheS	59H; 346R; 352V	RLI	0.4088	8	
yheS	59H; 346R; 352V	HQV	0.0040	9	
yheS	59H; 346R; 352V	HRV	0.1426	9	
yheS	59H; 346R; 352V	HQI	0.0070	10	
yheS	59H; 346R; 352V	HLV	0.2672	15	

Table S3. Effects on protein stability for mutations at sites under selection. ΔΔG was estimated by FoldX.

Gene	Model_8_site	Major Allele	Minor Allele	Charge Relationship	Polarity Relationship	Hydrophobicity Relationship	ΔΔG	ΔΔG Stability
csd	321	T	K	Uncharged → Positive	No change	Neutral → Hydrophilic	-0.0042669	No change
csd	321	T	S	No change	No change	No change	-0.188721	No change
cysG	32	S	V	No change	Polar → Non-polar	Neutral → Hydrophobic	0.342965	No change
cysG	32	S	A	No change	Polar → Non-polar	Neutral → Hydrophobic	0.765748	No change
cysG	242	I	V	No change	No change	No change	0.891478	No change
cysG	242	I	S	No change	Non-polar → Polar	Hydrophobic → Neutral	2.10838	No change
cysG	242	I	T	No change	Non-polar → Polar	Hydrophobic → Neutral	1.08852	No change
cysG	270	K	R	No change	No change	No change	-0.272011	No change
group_10276	107	K	N	Positive → Uncharged	No change	No change	0.75918	No change
group_10276	107	K	R	No change	No change	No change	0.241006	No change
group_10276	167	D	C	Negative → Uncharged	Polar → Non-polar	Hydrophilic → Hydrophobic	0.28072	No change
group_10276	167	D	G	Negative → Uncharged	Polar → Non-polar	Hydrophilic → Neutral	0.269991	No change
group_1172	66	E	G	Negative → Uncharged	Polar → Non-polar	Hydrophilic → Neutral	0.575151	No change
group_1172	66	E	R	Negative → Positive	No change	No change	0.676709	No change
group_1172	160	C	R	Uncharged → Positive	Non-polar → Polar	Hydrophobic → Hydrophilic	-0.77064	Stabilising
group_1172	160	C	H	Uncharged → Positive	Non-polar → Polar	Hydrophobic → Neutral	0.316285	No change
group_1172	202	P	L	No change	No change	Neutral → Hydrophobic	0.477903	No change
group_1172	202	P	S	No change	Non-polar → Polar	No change	1.53576	No change
group_1172	202	P	Q	No change	Non-polar → Polar	Neutral → Hydrophilic	0.117983	No change
group_1349	183	Y	N	No change	No change	Neutral → Hydrophilic	1.52139	No change
group_1349	195	H	N	Positive → Uncharged	No change	Neutral → Hydrophilic	0.842942	No change
group_1349	195	H	Y	Positive → Uncharged	No change	No change	-0.0787561	No change
group_1349	213	G	L	No change	No change	Neutral → Hydrophobic	-0.413236	No change
group_1349	213	G	V	No change	No change	Neutral → Hydrophobic	3.94182	Destabilising
group_1349	218	A	V	No change	No change	No change	4.80603	Destabilising
group_1349	243	H	P	Positive → Uncharged	Polar → Non-polar	No change	2.32648	No change
group_1349	243	H	S	Positive → Uncharged	No change	No change	0.85194	No change
group_2443	151	R	P	Positive → Uncharged	Polar → Non-polar	Hydrophilic → Neutral	0.117905	No change
group_2443	151	R	H	No change	No change	Hydrophilic → Neutral	2.78651	Destabilising
group_2443	156	N	G	No change	Polar → Non-polar	Hydrophilic → Neutral	0.844476	No change
group_2443	156	N	E	Uncharged → Negative	No change	No change	-3.39318	Stabilising
group_2443	214	L	P	No change	No change	Hydrophobic → Neutral	-1.69516	Stabilising
group_2443	214	L	S	No change	Non-polar → Polar	Hydrophobic → Neutral	2.33127	No change
group_2443	214	L	T	No change	Non-polar → Polar	Hydrophobic → Neutral	-2.11046	Stabilising

group_2443	552	I	L	No change	No change	No change	-0.435657	No change
group_2443	584	V	G	No change	No change	Hydrophobic → Neutral	-1.31266	Stabilising
group_2443	584	V	S	No change	Non-polar → Polar	Hydrophobic → Neutral	-0.0857338	No change
group_3177	463	S	A	No change	Polar → Non-polar	Neutral → Hydrophobic	0.162807	No change
hisI	122	A	D	Uncharged → Negative	Non-polar → Polar	Hydrophobic → Hydrophilic	0.550704	No change
hisI	122	A	G	No change	No change	Hydrophobic → Neutral	0.321089	No change
hisI	122	A	S	No change	Non-polar → Polar	Hydrophobic → Neutral	0.280007	No change
ileS	588	V	F	No change	No change	No change	3.73832	Destabilising
nasD	27	Q	P	No change	Polar → Non-polar	Hydrophilic → Neutral	0.00978344	No change
nasD	27	Q	T	No change	No change	Hydrophilic → Neutral	0.541334	No change
nasD	757	V	I	No change	No change	No change	-0.453537	No change
ndhB	158	W	R	Uncharged → Positive	Non-polar → Polar	Hydrophobic → Hydrophilic	-0.0246748	No change
ndhB	441	L	R	Uncharged → Positive	Non-polar → Polar	Hydrophobic → Hydrophilic	0.362183	No change
ndhB	441	L	S	No change	Non-polar → Polar	Hydrophobic → Neutral	0.798534	No change
rluD_3	77	V	I	No change	No change	No change	-0.737796	Stabilising
rluD_3	174	A	E	Uncharged → Negative	Non-polar → Polar	Hydrophobic → Hydrophilic	0.482222	No change
rluD_3	174	A	V	No change	No change	No change	-0.834728	Stabilising
rluD_3	174	A	T	No change	Non-polar → Polar	Hydrophobic → Neutral	0.32487	No change
rluD_3	179	R	L	Positive → Uncharged	Polar → Non-polar	Hydrophilic → Hydrophobic	1.0552	No change
rluD_3	179	R	H	No change	No change	Hydrophilic → Neutral	1.2986	No change
rluD_3	201	R	K	No change	No change	No change	0.796368	No change
sirC	54	T	M	No change	Polar → Non-polar	Neutral → Hydrophobic	-1.27453	Stabilising
sirC	54	T	A	No change	Polar → Non-polar	Neutral → Hydrophobic	-0.891344	Stabilising
sirC	89	G	R	Uncharged → Positive	Non-polar → Polar	Neutral → Hydrophilic	-1.66694	Stabilising
sirC	89	G	K	Uncharged → Positive	Non-polar → Polar	Neutral → Hydrophilic	-1.44826	Stabilising
yfcA	13	A	T	No change	Non-polar → Polar	Hydrophobic → Neutral	-0.0557702	No change
yfcA	13	A	V	No change	No change	No change	0.363747	No change
yfcA	211	S	F	No change	Polar → Non-polar	Neutral → Hydrophobic	-0.731424	Stabilising
yfcA	211	S	I	No change	Polar → Non-polar	Neutral → Hydrophobic	-0.524146	Stabilising
yheS	59	H	R	No change	No change	Neutral → Hydrophilic	-0.921701	Stabilising
yheS	59	H	Q	Positive → Uncharged	No change	Neutral → Hydrophilic	-0.917351	Stabilising
yheS	59	H	N	Positive → Uncharged	No change	Neutral → Hydrophilic	0.107738	No change
yheS	346	L	R	Uncharged → Positive	Non-polar → Polar	Hydrophobic → Hydrophilic	-0.688361	Stabilising
yheS	346	L	Q	No change	Non-polar → Polar	Hydrophobic → Hydrophilic	-0.0813127	No change
yheS	352	I	V	No change	No change	No change	1.17471	No change
